# CRB1 is required for recycling by RAB11A+ vesicles in human retinal organoids

**DOI:** 10.1016/j.stemcr.2023.07.001

**Published:** 2023-08-03

**Authors:** Thilo M. Buck, Peter M.J. Quinn, Lucie P. Pellissier, Aat A. Mulder, Aldo Jongejan, Xuefei Lu, Nanda Boon, Daniëlle Koot, Hind Almushattat, Christiaan H. Arendzen, Rogier M. Vos, Edward J. Bradley, Christian Freund, Harald M.M. Mikkers, Camiel J.F. Boon, Perry D. Moerland, Frank Baas, Abraham J. Koster, Jacques Neefjes, Ilana Berlin, Carolina R. Jost, Jan Wijnholds

**Affiliations:** 1 Department of Ophthalmology, Leiden University Medical Center (LUMC), Leiden 2333 ZA, the Netherlands; 2 Netherlands Institute for Neuroscience, Royal Netherlands Academy of Arts and Sciences (KNAW), Amsterdam 1105 BA, the Netherlands; 3 Department of Cell & Chemical Biology, Leiden University Medical Center (LUMC), Leiden 2300 RC, the Netherlands; 4 Bioinformatics Laboratory, Epidemiology & Data Science, Amsterdam University Medical Centers, Amsterdam 1105 AZ, the Netherlands; 5 Leiden University Medical Center hiPSC Hotel, Leiden 2333 ZA, the Netherlands; 6 Department of Ophthalmology, Amsterdam University Medical Centers, Academic Medical Center, University of Amsterdam, Amsterdam 1000 AE, the Netherlands; 7 Department of Genome Analysis, Amsterdam University Medical Centers, Amsterdam 1105 AZ, the Netherlands; 8 Department of Clinical Genetics/LDGA, Leiden University Medical Center, Leiden 2333 ZA, the Netherlands

**Keywords:** CRB1, NOTCH1, RAB11A, VPS35, retromer, WDFY1, endolysosomal system, autophagy, cell polarity, organoids, retina

## Abstract

*CRB1* gene mutations can cause early- or late-onset retinitis pigmentosa, Leber congenital amaurosis, or maculopathy. Recapitulating human *CRB1* phenotypes in animal models has proven challenging, necessitating the development of alternatives. We generated human induced pluripotent stem cell (iPSC)-derived retinal organoids of patients with retinitis pigmentosa caused by biallelic *CRB1* mutations and evaluated them against autologous gene-corrected hiPSCs and hiPSCs from healthy individuals. Patient organoids show decreased levels of CRB1 and NOTCH1 expression at the retinal outer limiting membrane. Proximity ligation assays show that human CRB1 and NOTCH1 can interact via their extracellular domains. *CRB1* patient organoids feature increased levels of WDFY1+ vesicles, fewer RAB11A+ recycling endosomes, decreased VPS35 retromer complex components, and more degradative endolysosomal compartments relative to isogenic control organoids. Taken together, our data demonstrate that patient-derived retinal organoids enable modeling of retinal degeneration and highlight the importance of CRB1 in early endosome maturation receptor recycling in the retina.

## Highlights

• CRB1 is associated with retinitis pigmentosa
• We made organoids from these patients and studied their biology
• The extracellular domains of CRB1 and NOTCH1 interact
• Our data link to altered endosomal maturation and increased autophagy

## Graphical abstract

**Figure:**
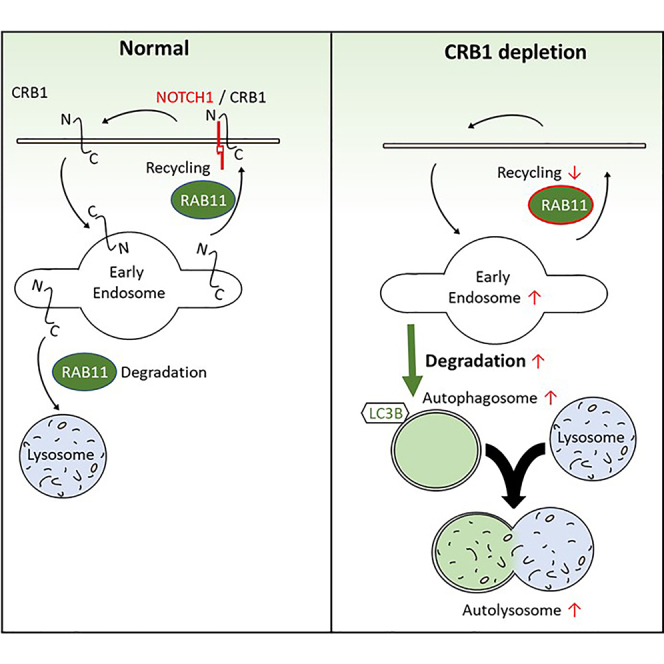

## Teaser

Wijnholds, Buck, and colleagues detect upregulation of the endolysosome-associated gene Wdfy-1 in a Crb1 mouse model. They also show that the extracellular domains of human CRB1 and NOTCH1 interact and levels of WDFY1 increased in human retinal organoids, whereas levels of CRB1, NOTCH1, VPS35, and RAB11A decreased in CRB1-associated retinitis pigmentosa organoids. CRB1 organoids repressed early endosome maturation and increased endosomal degradative compartments.

## Introduction

In mammals, the Crumbs (CRB) protein family consists of CRB1, CRB2, and CRB3A, with the latter lacking a large extracellular domain. Mutations in the *CRB1* gene are responsible for retinal diseases such as retinitis pigmentosa (RP), Leber congenital amaurosis (LCA), and macular dystrophy (Nguyen et al., 2022). Cell adhesion and cell polarity protein complexes at the retinal outer limiting membrane (OLM), such as adherens junctions and CRB complexes, play a critical role in proliferation of retinal progenitor cells (RPCs) (Pellissier et al., 2013) as well as in cell adhesion (Quinn et al., 2018, 2019b; van de Pavert et al., 2007). The CRB complex is located at the subapical region adjacent to the adherens junctions between RPCs in the developing retina and at the subapical regions of Müller glial cells (MGCs), photoreceptor cells (PRCs), PRC-PRCs, and PRC-MGCs.

We have described previously that the levels of CRB-family proteins at the apical membrane in photoreceptors and MGCs are major determinants of the severity of the retinal phenotype in *Crb1*^KO^ (KO, knockout) and *Crb1*^KO^*Crb2*^cKO^ (cKO, conditional KO) mice compared with controls. In contrast to CRB1, the CRB2 protein is present in photoreceptors and MGCs in both species (Quinn et al., 2019b; van Rossum et al., 2006). We showed that RP *CRB1* patient-derived retinal organoids expressing different variants of CRB1 proteins (*CRB1*^M1041T/M1041T^; *CRB1*^Y631C/E995∗^; *CRB1*^M1041T/C948Y^) have little cell polarity and cell adhesion markers on differentiation day 180 (DD180) and significantly lowered levels of variant CRB1 proteins, permitting local displacement of PRC columns (Boon et al., 2023; Quinn et al., 2019a). Loss of apical polarity has been linked previously to dysregulation of the endolysosomal system (Boon et al., 2023; Kraut and Knust, 2019; Lattner et al., 2019; Pocha et al., 2011; Zhou et al., 2011). The membrane vesicle trafficking system has been associated with many diseases but, to our knowledge, has not been studied in detail in human retinal organoids.

Numerous reports have previously demonstrated a link between loss of the CRB complex and activation of the Notch signal pathway (Laprise, 2011; Pellissier et al., 2013; Thompson et al., 2013). A direct interaction between extracellular domains (ECDs) of NOTCH-CRB and CRB-CRB was observed in fruit fly and zebrafish models (Laprise, 2011; Nemetschke and Knust, 2016; Ohata et al., 2011; Zou et al., 2012). The ECD of CRB in the fruit fly or of CRB2 in the zebrafish stabilizes the NOTCH receptor at the apical membrane (Herranz et al., 2006; Lattner et al., 2019; Nemetschke and Knust, 2016; Ohata et al., 2011). Furthermore, the fruit fly CRB mutant protein (*crb*^P13A9^) decreases NOTCH receptor localization at the plasma membrane; increases NOTCH intracellular domain (ICD) concentrations, which activates the NOTCH pathway; and expands the number of lysosomes in rhabdomeres (a cluster or photoreceptors and support cells in flies) compared with controls (Kraut and Knust, 2019).

Patients with the common RP *CRB1*-associated missense mutations (*CRB1*^Met1041Thr^; *CRB1*^Cys948Tyr^) have significantly lowered levels of CRB1 in the retina and a disturbed endolysosomal system, thus benefitting from gene supplementation therapy (Boon et al., 2023; Buck et al., 2021; Pellissier et al., 2015). In the present study, we identified target genes involved in the molecular mechanisms of the *CRB1* LCA-like phenotype in mice. We found that *Wdfy-1* is a gene overexpressed in the *CRB1* LCA-like mouse model. The WD repeat and FYVE domain-containing 1 (WDFY1) protein is found on the early endosome (Ridley et al., 2001). WDFY1 is localized on lysosomes, being part of an E3 ubiquitin ligase complex involved in selective autophagy, termed lysophagy, recognizing and removing damaged lysosomes (Teranishi et al., 2022). To analyze the role of CRB1 in endosome pathways, we studied induced pluripotent stem cell (iPSC)-derived human retinal organoids. We find a strong decrease in the level of apical CRB1 variant and apical NOTCH1 proteins at the OLM on DD180. We then trace back how the decreased levels of CRB1 variant proteins appear by investigating the trafficking and degradation of CRB1 proteins. We find that the significant decrease in CRB1 variant protein coincides with an increase of WDFY1 at the OLM and in the outer nuclear layer (ONL); repression of maturation of early endosomes (EEs), shown by an increase of EEA1 in EEs; a reduction of RAB11A+ recycling endosomes and VPS35/retromer vesicles; and an increase in degradative endolysosomal compartments in patient organoids. This study suggests that CRB1 is involved in EE/recycling endosome maturation.

## Results

### RNA sequencing (RNA-seq) data from developing *Crb1*- and *Crb2*-deficient mouse retina reveals a molecular defect in the endolysosomal system

**Figure 1:**
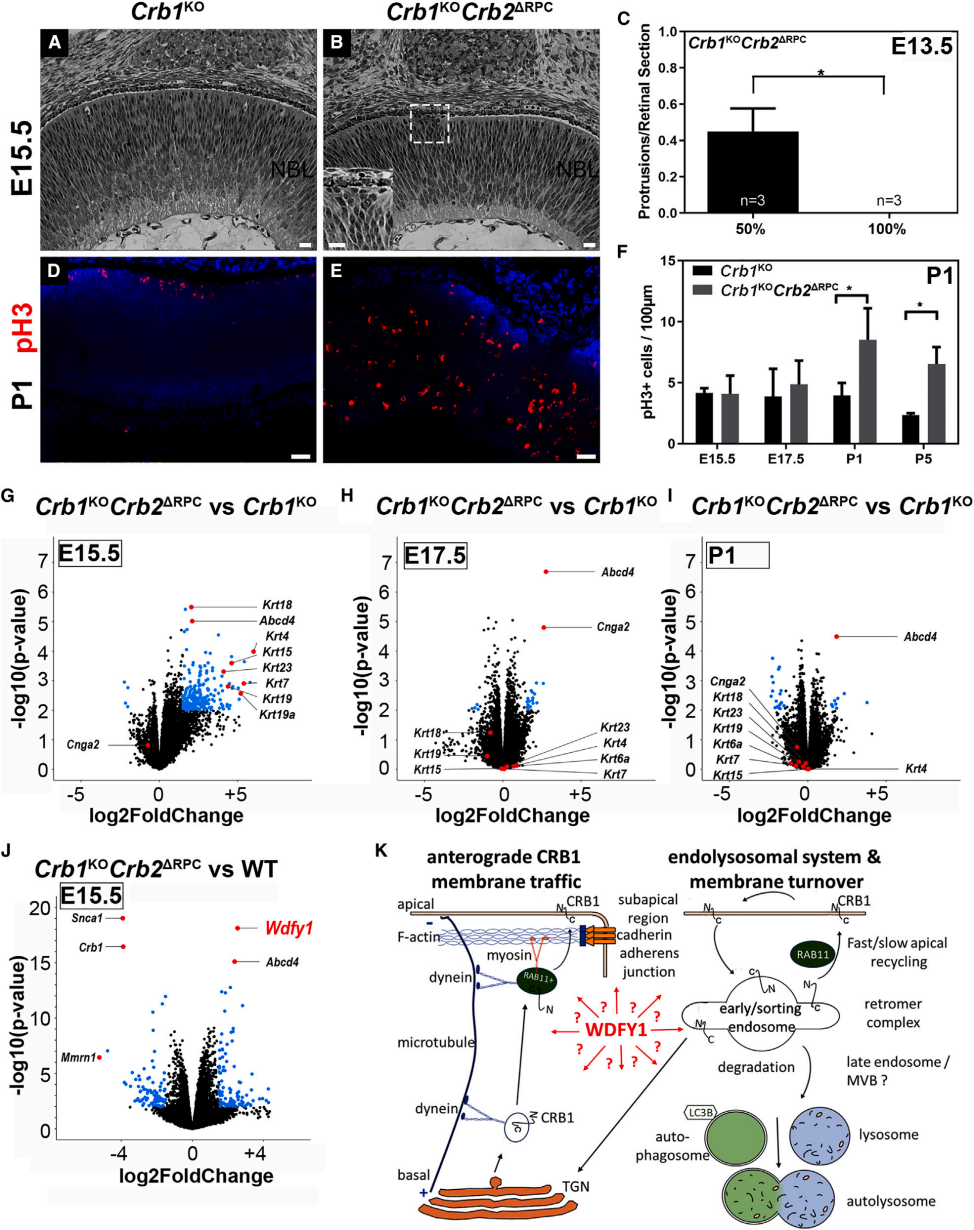
mRNA transcript levels are marginally different in *Crb1*^KO^*Crb2*^ΔRPC^ against *Crb1*^KO^ retina (A and B) Retinal morphology on plastic sections of *Crb1*^KO^ and *Crb1*^KO^*Crb2*^ΔRPC^ mice on a 100% C57/B6 genetic background at embryonic day 15.5 (E15.5). (A) *Crb1*^KO^ retinas appeared to be unaffected, while (B) *Crb1*^KO^*Crb2*^ΔRPC^ retinas had protrusions of neuroblast nuclei in the subretinal space (inset). (C) Protrusions per retinal section in a 50% and 100% C57/B6 genetic background at E13.5. (D–J) 100% C57/B6 genetic background. (D and E) Immunofluorescence labeling of pHH3+ nuclei (late G2 cell cycle and mitosis maker) on postnatal day P1 in (D) *Crb1*^KO^ and (E) and *Crb1*^KO^*Crb2*^ΔRPC^ retina. (F) Quantification of pHH3+ cells on E15.5, E17.5, P1, and P5. ^∗^p < 0.05. (G–J) All changed genes are indicated in blue circles (p < 0.01; log2(Fold Change) > 1.5). (G–I) *Crb1*^KO^*Crb2*^ΔRPC^ against *Crb1*^KO^ retina transcripts (run 1) on E15.5, E17.5, and P1. (J) *Crb1*^KO^*Crb2*^ΔRPC^ compared with WT retina transcripts on E15.5 (run 2). (K) Potential WDFY1 involvement in CRB1 protein trafficking (modified from Aguilar-Aragon et al., 2020). Scale bars, 20 μm. WT, wild type; TGN, *trans*-Golgi network; MVB, multivesicular body. See also Figures S1 and S2.

To identify the molecular mechanisms of developmental degradation in the retina lacking CRB1 and CRB2, we first examined the retina from *Crb1*^KO^ (*Crb1*^KO^*Crb2*^Flox/Flox^) and *Crb1*^KO^*Crb2*^ΔRPC^ cKO mice backcrossed into 100% C57BL/JOlaHsd mice. In agreement with previous findings in a mixed genetic background (Pellissier et al., 2013), our analyses of the retina from *Crb1*^KO^*Crb2*^ΔRPC^ mice show that CRB1 and CRB2 are essential for proper retinal development, preventing disturbed retinal layering and loss of retinal function (Figures 1A, 1B, S1, and S2). Of note, the 100% C57BL6/JOlaHsd genetic background against the mixed C57Bl/6 and 129/Ola 50%/50% background shows a slightly milder retinal phenotype at foci (Figures 1C and S1). Some regions in the retina mimic the previously described milder heterozygous *Crb1* conditional homozygous *Crb2* (*Crb1*^WT/KO^*Crb2*^ΔRPC^) cKO retinal phenotype, whereas other regions mimic the more severely affected double homozygote *Crb1*^KO^*Crb2*^ΔRPC^ retina (Pellissier et al., 2013). The severity of the phenotype was still comparable with the mixed genetic background, considering retinal cell loss measured by retinal thickness (Figure S2H) and the loss of the retinal response to light flashes measured by electroretinography (Figures S2I–S2K) in 1-month-old mice (Pellissier et al., 2013). Previous studies on developing *Crb1*^KO^*Crb2*^ΔRPC^ retinas on a mixed genetic background showed a transiently thicker retina with an increased number and mislocalization of late-born cells (rod photoreceptors, bipolar cells, MGCs, and late-born amacrine cells), increased cell proliferation and apoptosis, and dysregulation of the cell cycle, which severely impairs retinal function in adult mice. It has been speculated that CRB1 and CRB2 suppress RPC proliferation by regulating mitogenic signaling pathways such as NOTCH, YAP, and Sonic Hedgehog (Pellissier et al., 2013). We also found upregulation of phosphohistone H3+ (pHH3+) mitotic cells on post-natal day 1 (P1) and P5 (Figure 1F), indicative of increased cell proliferation in the 100% C57BL/6JOlaHsd background.

We therefore performed RNA-seq on mouse retina to gain insights into these developmental changes at the transcriptional level. A comparison between *Crb1*^KO^*Crb2*^ΔRPC^ and *Crb1*^KO^ retina, on 100% C57BL/6JOlaHsd, yielded only subtle persistent changes at the transcriptional level over time (Figures 1G–1I, respectively), despite significant differences in morphology (Alves et al., 2013a, 2013b). As an internal positive control, we used ATP-binding cassette, subfamily D (ALD), member 4 (*Abcd4*), which is substantially upregulated in mouse retina expressing the *Cre* recombinase fused to the *Chx10* promoter, with the *Abcd4* gene immediately adjacent to the *Chx10Cre* locus in the mouse genome. *Abcd4* expression and other genes were also validated by qPCR on P1 (Figure S2L).

To further examine potential transcriptional changes on embryonic day 15.5, we compared *Crb1*^KO^*Crb2*^ΔRPC^ mice in a 100% C57BL/6JOlaHsd genetic background against wild-type C57BL/6JRccHsd mice. In this setting, we observed 40 differentially expressed genes (DEGs) compared with the previously 0 DEGs (adjusted p value < 0.01, log2(Fold Change) > 1.5). The *Crb1*^KO^*Crb2*^ΔRPC^ mice on the C57BL/6JOlaHsd genetic background, because of mutations in the synuclein alpha (*Snca*), multimerin-1 (*Mmrn1*), and *Crb1* genes, do not express *Snca*, *Mmrn1*, or *Crb1* gene transcripts and express high levels of *Abcd4* because of the adjacent *Chx10Cre* transgene on chromosome 6. We used all 4 genes as negative and positive controls in gene expression profiling because the mice on the C57BL/6JRccHsd genetic background have no mutations in *Snca*, *Mmrn1*, or *Crb1* and express low levels of *Abcd4* (Gajovic et al., 2006). Analysis of the revealed upregulated expression of the WD repeat and FYVE domain-containing protein 1 (*Wdfy1*) gene, also known as FYVE domain-containing protein localized to endosomes (*Fens-1*), which encodes a phosphatidylinositol 3-phosphate binding protein that contains a FYVE zinc-finger domain and multiple WD-40 repeat domains (Figure 1J). The misregulation in EEs by WDFY1 is in line with the data from *Crb* fruit fly genetics (Kraut and Knust, 2019; Lattner et al., 2019; Pocha et al., 2011), which show protein aggregation and expression of immature apical surface markers linked to the endolysosomal system. The *CRB1* RP-like morphologic phenotype in the mouse, the surprisingly subtle differences on transcript levels (*Crb1*^KO^*Crb2*^ΔRPC^ against wild-type mice) with *Wdfy1* being upregulated and potentially localizing on EEs, and single cell RNA sequencing (scRNA-seq) transcription profile studies on *CRB1* RP retinal organoids that suggested involvement of the endosomal system (Boon et al., 2023) prompted us to investigate whether variant CRB1 proteins affect the early endosomal system in *CRB1* RP-like human patient-derived retinal organoids (Figure 1K).

### *CRB1* patient retinal organoids bearing missense variations show reduced levels of CRB1 protein

To investigate the significantly reduced levels of variant CRB1 protein in *CRB1* patient retinal organoids, we generated isogenic controls (Boon et al., 2023). Isogenic controls were generated by homology-directed repair (HDR) of the *CRB1*^M1041T^ variant using CRISPR-Cas9 (Figure S3; Table S1). Wild-type CRB1 and variant CRB1 proteins (encoded by *CRB1*^M1041T/M1041T^, *CRB1*^M1041T/C948Y^, or *CRB1*^Y631C/E995∗^) were monitored by fluorescence over time using two different antibodies recognizing either an epitope on the short intracellular domain (ICD) of CRB1 or the first epidermal growth factor (EGF)-like domains of the extracellular domain (ECD). The CRB1-ECD/CRB1-ICD antibodies have a high CRB1 antigen specificity with little background observed in immuno-electron microscopy (EM) and immunohistochemistry on human donor cadaveric retinas (Pellissier et al., 2014). Both antibodies indicated clear CRB1 expression in the isogenic and control organoids on DD180 (Figures S4A–S4J). No CRB1-ECD/ICD overlapping signal was found on DD90/DD120 (Figures S4F and S4G), which is at the onset of CRB1 expression (DD120) found previously (Quinn et al., 2019a). As reported recently (Boon et al., 2023), a very weak variant CRB1 protein signal was observed at the OLM in the *CRB1* patient lines compared with the isogenic or control retinal organoids on DD180 (Figures S4A, S4B, and S5A–S5H).

The lower levels at the OLM of *CRB1*^M1041T^ (patient LUMC0116iCRB09), *CRB1*^M1041T/C948Y^ (patient LUMC0128iCRB01), or *CRB1*^Y631C/E995∗^ (patient LUMC0117iCRB01) proteins in *CRB1* RP retinal organoids compared with control organoids are not due to changes in the *CRB1* transcripts. scRNA-seq on *CRB1* patients LUMC0116iCRB09 and LUMC0128iCRB01 versus their isogenic control retinal organoids on DD230 showed no statistically significant difference in *CRB1* variant versus *CRB1* wild-type mRNA expression, respectively (Boon et al., 2023). Unfortunately, lysates of wild-type DD180 or DD210 retinal organoids showed no specific CRB1 or CRB2 bands on western blots using anti-CRB1 or anti-CRB2, respectively. The reduction of CRB1 variant protein expression at the OLM suggests that patient-derived missense gene variations may alter the trafficking of CRB1-containing vesicles to the OLM or CRB1 turnover at the OLM.

### CRB1/NOTCH1-ECD interact, and variant CRB1 reduces apical NOTCH1 expression

We found a strong reduction of the adhesion markers β-catenin, p120-catenin, N-cadherin, PAR3, MUPP1, and PALS1 at the OLM on DD180 (Quinn et al., 2019a). NOTCH signaling is involved in cell proliferation and cell adhesion. For example, NOTCH signals from the mouse retinal pigment epithelium contributed to NOTCH activation in adjacent RPCs (Ha et al., 2017). *Notch1* is essential for maintaining the RPC population (cycling cells) in the developing retina. Conditional knockdown of *Notch1* restricts the RPC subpopulation and confines the differentiation capacity of RPCs mainly to cone photoreceptors (Chen and Emerson, 2021). Also, the fruit fly CRB mutant protein (CRB^P13A9^) decreases NOTCH receptor localization (Kraut and Knust, 2019).

**Figure 2:**
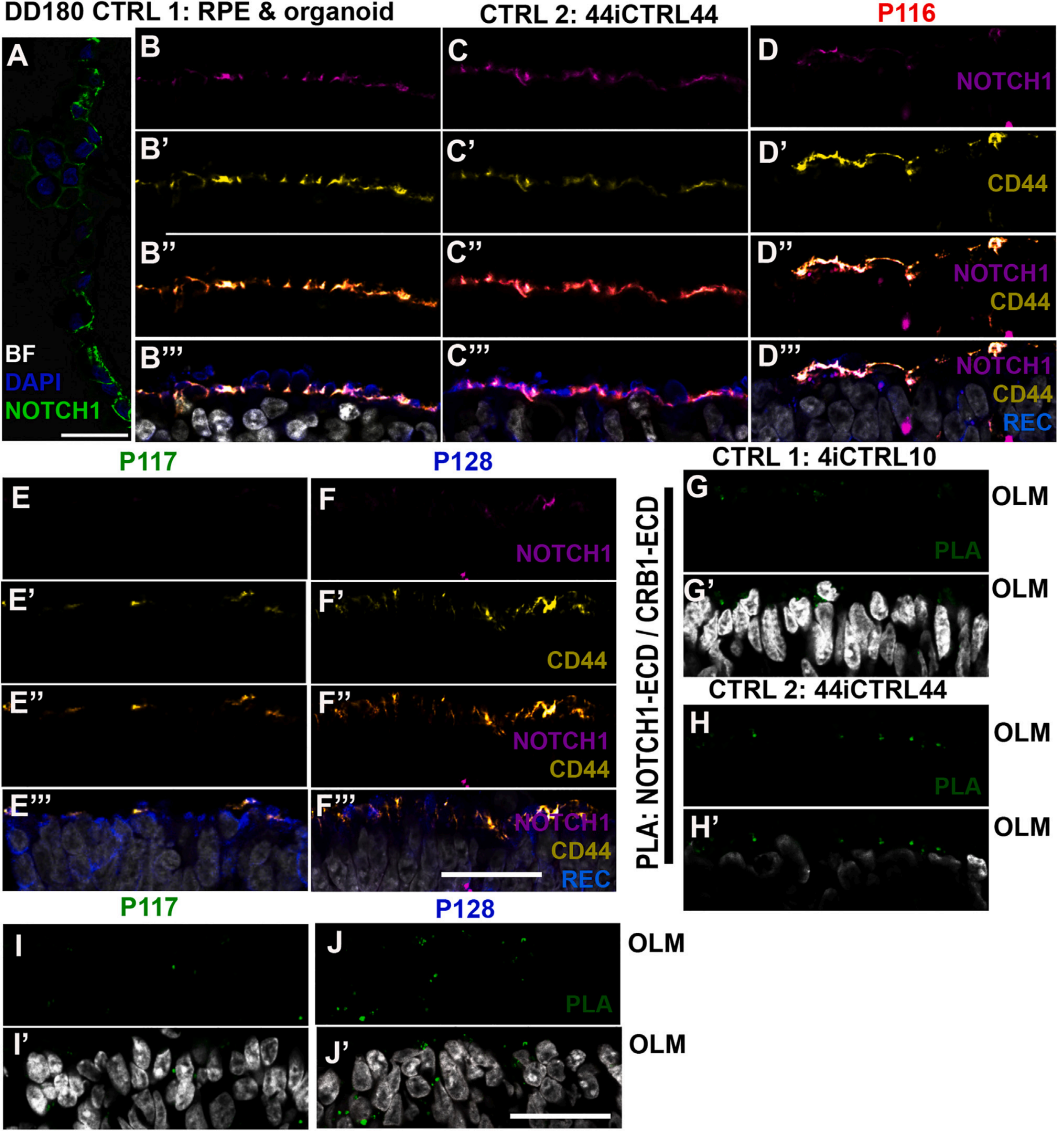
Apical NOTCH1 is lost in *CRB1* patient retinal organoids (A) CTRL1 (LUMC004iCTRL10) RPE expresses NOTCH1 on DD180 (BF, bright field; to indicate pigments in the RPE). (B–F) CTRL1 (B), CTRL2 (LUMC044iCTRL44) (C), patient P116 (LUMC0116iCRB09) (D), patient P117 (LUMC0117iCRB01) (E), and patient P128 (LUMC0128iCRB01) (F). (A–F) NOTCH1 is expressed specifically in DD180 Müller glial apical villi (magenta, NOTCH1; yellow, CD44; blue, recoverin; gray, DAPI). (G–J) PLA of NOTCH1-ECD and CRB1-ECD (green signal) shows interaction at CTRL OLM but reduced interaction at patient OLM with increased localization in patient ONL. Scale bars, 25 μm.

NOTCH1 was expressed in the retinal pigment epithelium (RPE) at all time points, serving as a positive control (Figure 2A). At DD180, NOTCH1 expression was almost exclusively limited to Müller glial-villi at the OLM, co-labeled with CD44-Müller glial-villi marker and not with the photoreceptor/on-bipolar marker recoverin (Figures 2B and 2C). *CRB1* patient retinal organoids expressed lower levels of NOTCH1 at the OLM (Figures 2D–2F) and increased levels of NOTCH1 in the inner retina. Recently, a NOTCH-CRB interaction on the ECD was found by proximity ligation assay (PLA) in the fruit fly (Lattner et al., 2019). We observed an interaction of CRB1/NOTCH1 ECDs by PLA on human retinal organoids in patient and control retinal organoids (Figures 2G–2J). The interaction signal in patient retinal organoids was reduced at the OLM and increased in the ONL (Figures 2G–2J). The close proximity between NOTCH1 and CRB proteins is in accordance with the literature (Laprise, 2011; Nemetschke and Knust, 2016; Ohata et al., 2011; Zou et al., 2012).

### Increased autophagy-lysosomal compartments in *CRB1* retinal organoids

**Figure 3:**
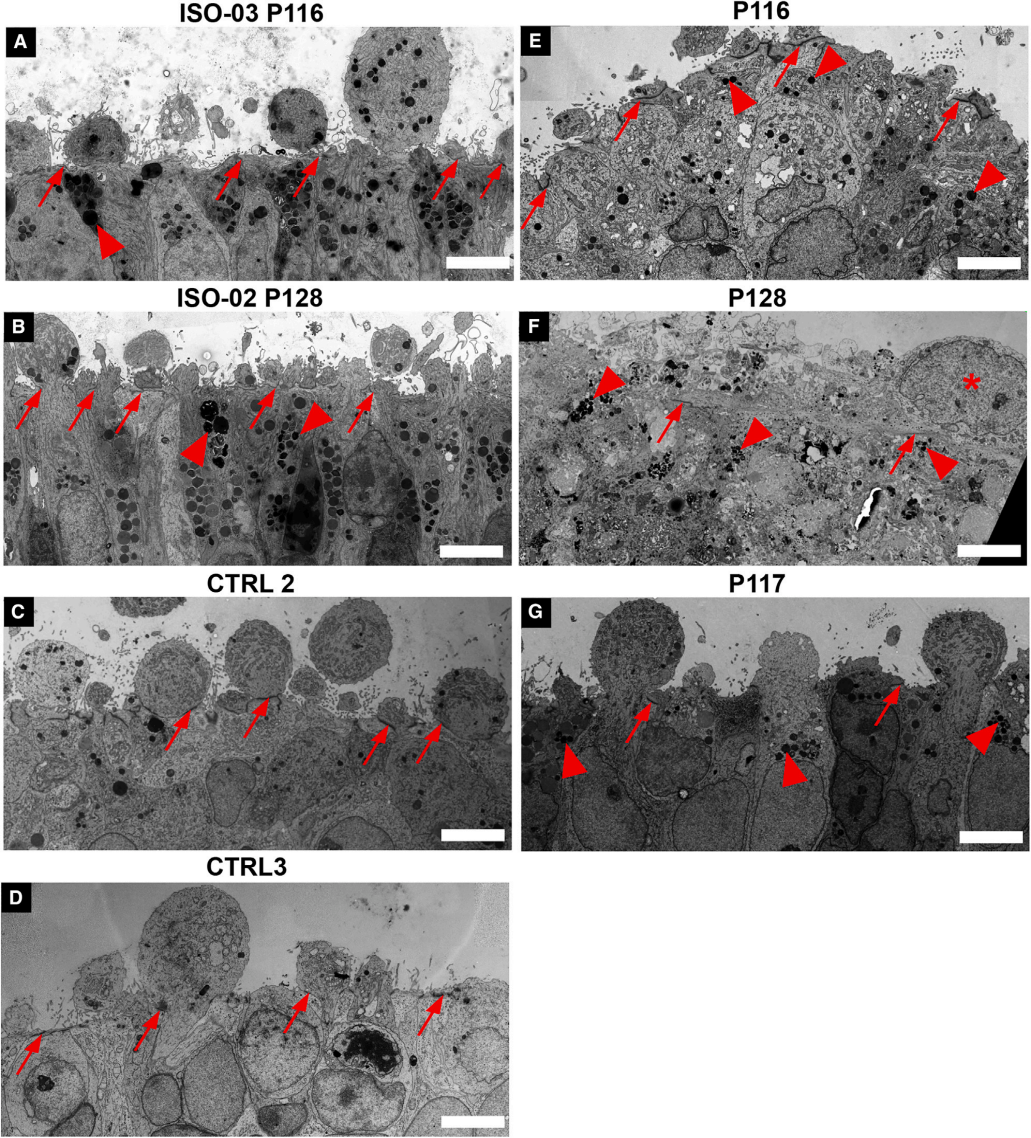
High-resolution TEM imaging of retinal organoids on DD180 Shown are electron-dense OLM (red arrows), electron-dense degradative compartments/vacuoles (arrowheads), and nuclei above the OLM (asterisks). (A and B) Gene-corrected lines (ISO-03 P116, iso3LUMC0116iCRB09; ISO-02-P128, iso2LUMC0128iCRB01). (C and D) CTRL lines (CTRL2, LUMC0044iCTRL44; CTLR3, LUMC080iCTRL12). (E–G) Patient lines P116, P128, and P117 (LUMC0116iCRB09, LUMC0128iCRB01, and LUMC0117iCRB01, respectively). Scale bars, 5 μm. Related to Figure S3.

**Figure 4:**
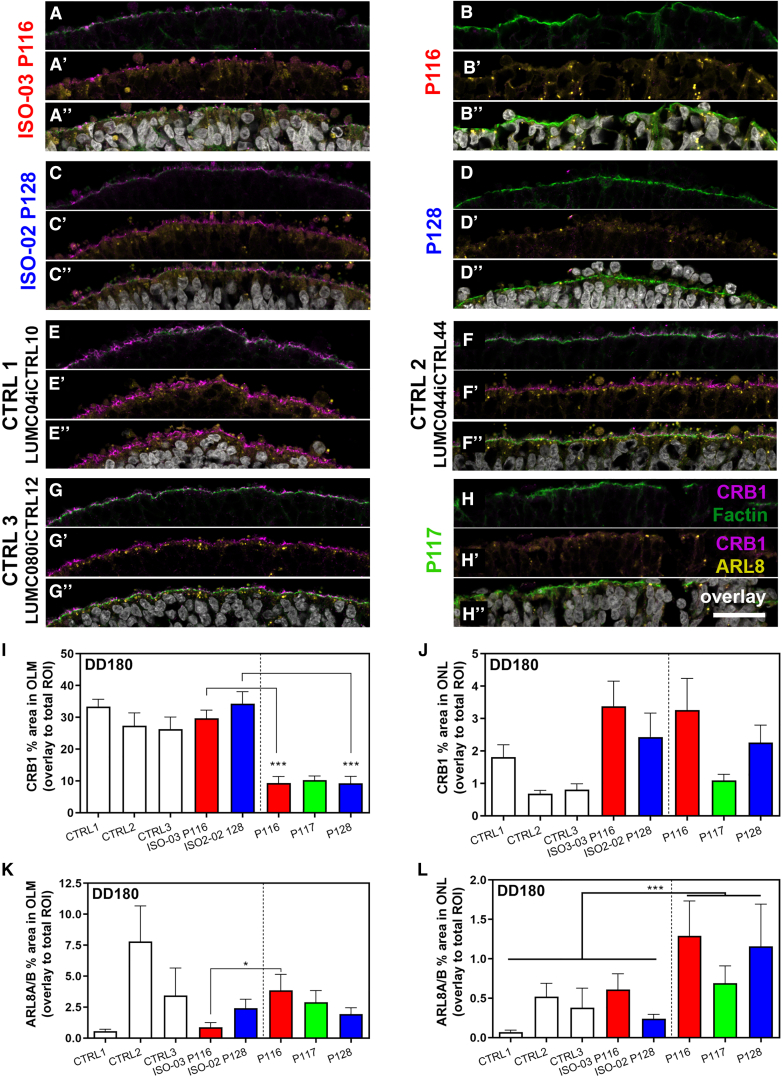
The immunofluorescence signal of CRB1 labeling is reduced in *CRB1* patient retinal organoids at the OLM (A–H) Triple staining of CRB1-ICD (AK2 antibody, magenta), phalloidin (filamentous actin [F-actin], green), and ARL8A/B (yellow). (I and J) CRB1 fluorescence signal measured by puncta in the OLM and ONL. (K and L) CRB1 fluorescence signal measured by puncta in the OLM and ONL. Scale bars, 25 μm. Each data point in the graphs represents individual organoids, of which an average was taken of 3 representative images. The SEM is derived from these averages. The numbers of individual organoids per condition (CTRL1, CTRL2, CTRL3, ISO-03 P116, ISO-02 P128, P116, P117, and P128) in (I) and (J) are 9, 9, 9, 10, 10, 12, 9, and 13, respectively, and for (K) and (L) 7, 8, 3, 8, 8, 12, 6, and 8, respectively (from at least two independent organoid batches). Statistical analysis: ^∗^p < 0.05, ^∗∗∗^p < 0.001. Related to Figures S4 and S5.

Loss of CRB increased NOTCH and DELTA protein endocytosis in the fruit fly (Richardson and Pichaud, 2010), which increases ARL8+ lysosomes in rhabdomeres compared with controls in a pre-disease state (Kraut and Knust, 2019; Lattner et al., 2019). We next sought to determine whether endolysosomal vesicles (size, morphology) are affected by loss of apical CRB1 using transmission electron microscopy (TEM; Figure 3) and then identified and quantified them using light microscopy (Figure 4) on DD180.

Using TEM, we found few electron-dense degradative compartments/vacuoles in control organoids (Figures 3A–3D, few arrowheads) compared with many in patient organoids (Figures 3E–3G, arrowheads) and, surprisingly, some in gene-corrected *CRB1* heterozygous organoids (Figures 3A and 3B, arrowheads). Additionally, the electron-dense adherens junction/subapical region appeared to be elongated in some patient organoids (Figures 3E–3G, red arrows indicate adherens junction/subapical regions). Furthermore, some cell disruptions were detected in *CRB1*^M1041T/C948Y^ organoids (Figure 3F, asterisks indicate nuclei above adherens junctions). Interestingly, we did not find definitive multivesicular bodies in any of the these specimens, suggesting that photoreceptors and MGCs may not utilize them during endosomal maturation.

The increase in electron-dense degradative compartments/vacuoles, seen using TEM, in patient retinal organoids was then quantified in DD180 retinal organoids using immunofluorescence microscopy. We hypothesized that the CRB1 variant is less efficiently shuttled to the OLM or, alternatively, that the turnover of CRB1 variant protein at the OLM is higher. However, we found no increase in the CRB1 variant in the inner/ONL, indicating little overall aggregation. Also, we observed minimal co-localization of the CRB1 variant with the general endolysosomal marker LAMP1 in the ONL/OLM (Figures S5A′–S5H′). We found more lysosomal LAMP1+ vesicles in the ONL, but not close to the OLM, in patient retinal organoids (Figures S5I and S5J). Then, we assessed the co-labeling and expression of the small GTPase lysosome kinesin adaptor ARL8A/B with CRB1 (Figure 4). ARL8A/B is found primarily on late lysosomes (Rosa-Ferreira and Munro, 2011). Also, ARL8A/B takes part in anterograde transport of mature lysosomes to the cell and co-mediates transport of endocytic cargo to lysosomes (Farfel-Becker et al., 2019; Rosa-Ferreira and Munro, 2011). ARL8+ lysosomes have been found previously to accumulate CRB variant proteins in the fruit fly (Kraut and Knust, 2019). We, however, also found little co-labeling of variant CRB1 with ARL8A/B (Figures 4A–4H). Similar to LAMP1, the area of total ARL8A/B (lysosome/late endosome) puncta was increased in the ONL of patient organoids (Figures 4K and 4L), implying accumulation of degradative compartments but not accumulation of CRB1 variant protein.

**Figure 5:**
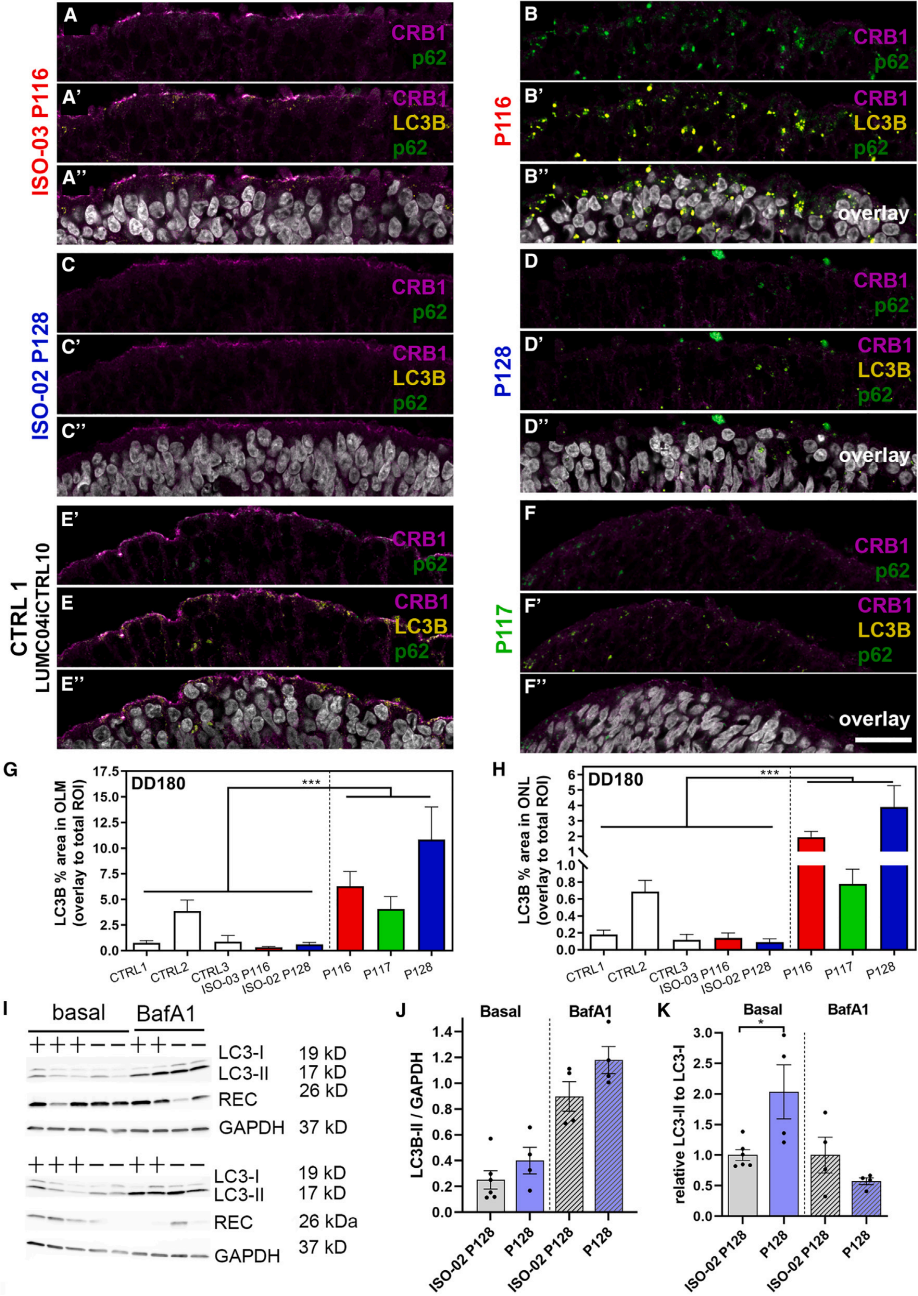
More degradative vesicles/compartments are present in *CRB1* patient retinal organoids (A–F) Immunofluorescence triple staining of CRB1-ICD (AK2 antibody, magenta), p62 (green), and LC3B (yellow). (G and H) LC3B localized more in the ONL and OLM layers in *CRB1* RP retinal organoids. (I–K) Western blots of individual organoid lysates (plus symbol, isogenic 2 line (iso02-128iCRB01); minus symbol, patient 3 (line LUMC0128CRB01) stained for LC3B (LC3-I and LC3-II; 19/17 kD; recoverin for photoreceptors (26 kD), and GAPDH (housekeeping CTRL, 37 kD). Autophagic flux was decreased in *CRB1* patient retinal organoids. Scale bars, 25 μm. Each data point in the graphs in (J) and (K) represents individual organoids, of which an average was taken. The SEM is derived from these averages. The numbers of individual organoids in (G) and (H) per condition (CTRL1, CTRL2, CTRL3, ISO-03 P116, ISO-02 P128, P116, P117, and P128) are 7, 6, 9, 8, 9, 10, 11, and 10, respectively (from at least two independent organoid batches). Statistical analysis: ^∗^p < 0.05, ^∗∗∗^p < 0.001. Related to Figures S5, S6, and S8.

We also examined CRB1 variant protein being co-labeled with classic autophagy markers, such as the ubiquitin-binding protein adapter p62 and the microtubule-associated proteins 1A/1B light chain 3B (LC3B) in the ONL/OLM. The autophagy cargo adapter p62 can transport substrates to the lysosome that have been tagged; e.g., by LIR (LC3-interacting motif for degradation) (Overhoff et al., 2021). p62 has an important role in selective autophagy of organelles and ubiquitinated misfolded/damaged proteins (here potentially the CRB1 variant protein). Further, p62 has been linked to selective stress-response-induced autophagy and proteostasis and is extensively implicated in selective autophagy (Overhoff et al., 2021; Pradhan et al., 2019; Wooten et al., 2008). We found a strong increase in LC3B and p62 presence in *CRB1* patient retinal organoids in the ONL and at the OLM (Figures 5 and S6). The increase may be related to changes in autophagic flux. We added BafA1 to inhibit acidification of endolysosomes by blocking the vacuole-ATPase (H^+^/Ca^2+^ antiporter), which is required for fusion of lysosomes with autophagosomes to form auto-lysosomes and, hence, for degradation of proteins targeted to lysosomes. We incubated retinal organoids with BafA1 (500 nM) for 6 h and extracted the lysates. BafA1 induced a robust block of lysosome-autophagosome fusion on individual organoids (Figures 5I–5K, S6C, and S6D). We found more autophagosome markers (LC3-II) in *CRB1* patient retinal organoids and decreased autophagic flux (Figures 5J, 5K, S6G, and S6H), pointing toward a blockage of autophagic vesicle removal. We also found little variant CRB1 expression and co-labeling of CRB1 with p62, LC3B, or ARL8A/B. Accordingly, CRB1 variant protein might be either expressed at low levels (Figure S4), unstable, degraded in the inner/ONL with little variant CRB1 reaching the OLM, or reach the OLM but show high turnover because of abnormal vesicle sorting. Taken together, we show by immunohistochemistry that CRB1 variant proteins are expressed at more than 4-fold lower levels than CRB1 protein in isogenic controls (Figure 4I). The PLA between CRB1 and NOTCH1 shows some remaining CRB1 variant signal at the OLM and mislocalization within the ONL (Figures 2I and 2J), suggesting that the CRB1 variant protein is translated and transported by the endosomal system to the OLM.

### Decreased levels of CRB1 reduce the pool of recycling endosomes

**Figure 6:**
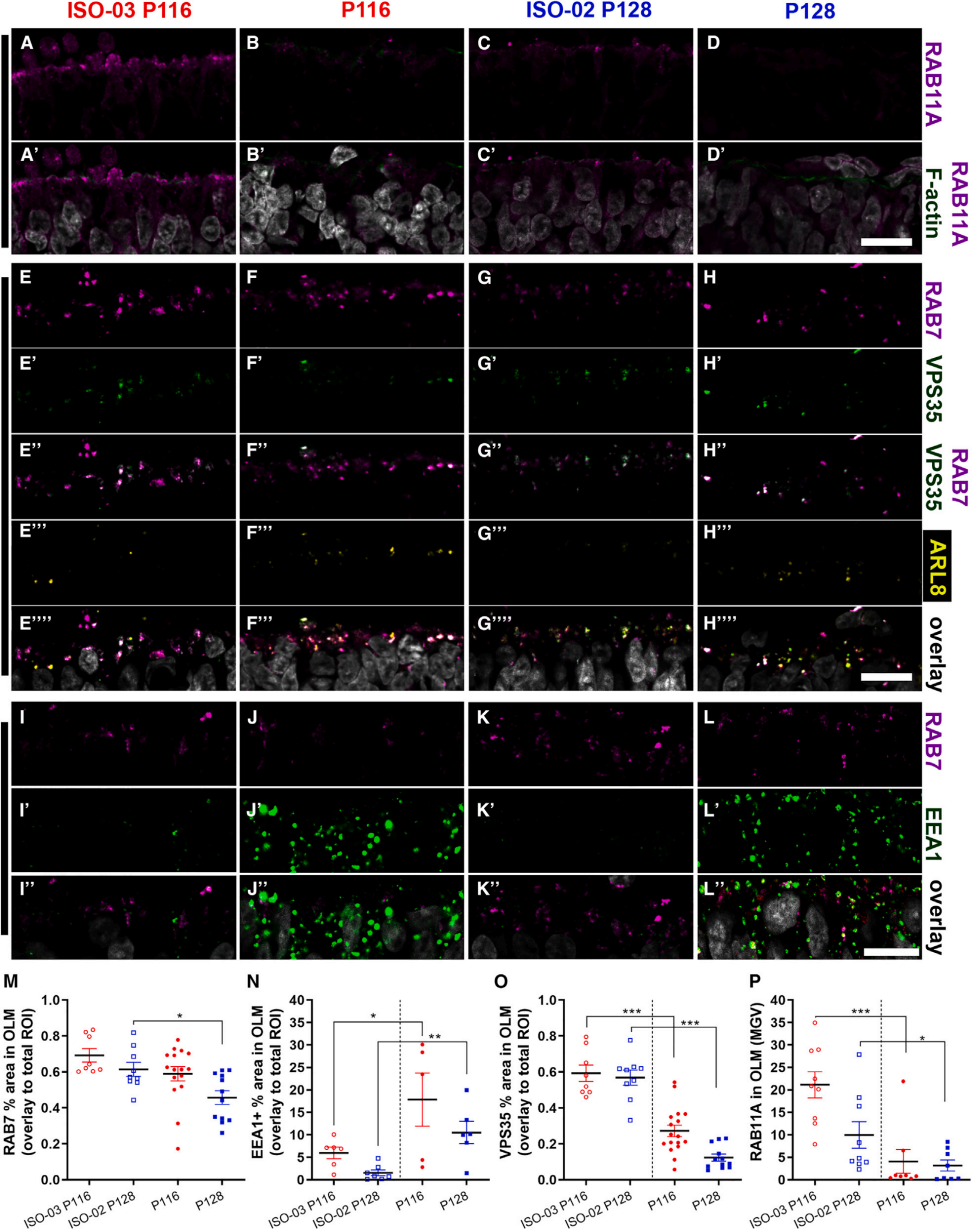
Dysregulation of the endolysosomal system in *CRB1* patient organoids (A–L) Immunofluorescence stainings, with the antibodies indicated on the right sides of the panels, on isogenic CTRL organoid lines 1 and 2 (A, C, E, G, I, and K) related to patient organoid lines 1 (LUMC0116iCRB09) and 3 (LUMC0128iCRB01) and lines 1 and 3 (B, D, F, H, and J–L). (A–D) Recycling endosomes (RAB11A, magenta) and phalloidin (F-actin, OLM, green). The OLM in retinal organoids is stained by F-actin. (E–H) Late endosomes (RAB7, magenta), retromer complex (VPS35, green), and endolysosomes (ARL8A/B, yellow). (I–L) Late endosomes (RAB7, magenta) and EEs (EEA1, green). (M–P) Quantification of the fluorescence signal of EEs (EEA1), late endosomes (RAB7), retromer (VSP35), and recycling endosomes (RAB11A) in the OLM. (M) RAB7 in the OLM. (N) EEA1 in the OLM. (O) VPS35 in the OLM. (P) RAB11A in the OLM. Scale bars, 10 μm. Each data point in the graphs represents individual organoids, of which an average was taken of 3 representative images. The SEM is derived from these averages. The numbers of individual organoids per condition (ISO-03 P116, ISO-02 P128, P116, and P117) for (M) and (O) are 8, 9, 16, and 12, respectively; for (N) 6, 7, 5, and 6, respectively; and for (P) 9, 9, 8, and 8, respectively (from at least two independent organoid batches). Statistical analysis: ^∗^p < 0.05, ^∗∗^p < 0.01, and ^∗∗∗^p < 0.001. Related to Figure S7.

**Figure 7:**
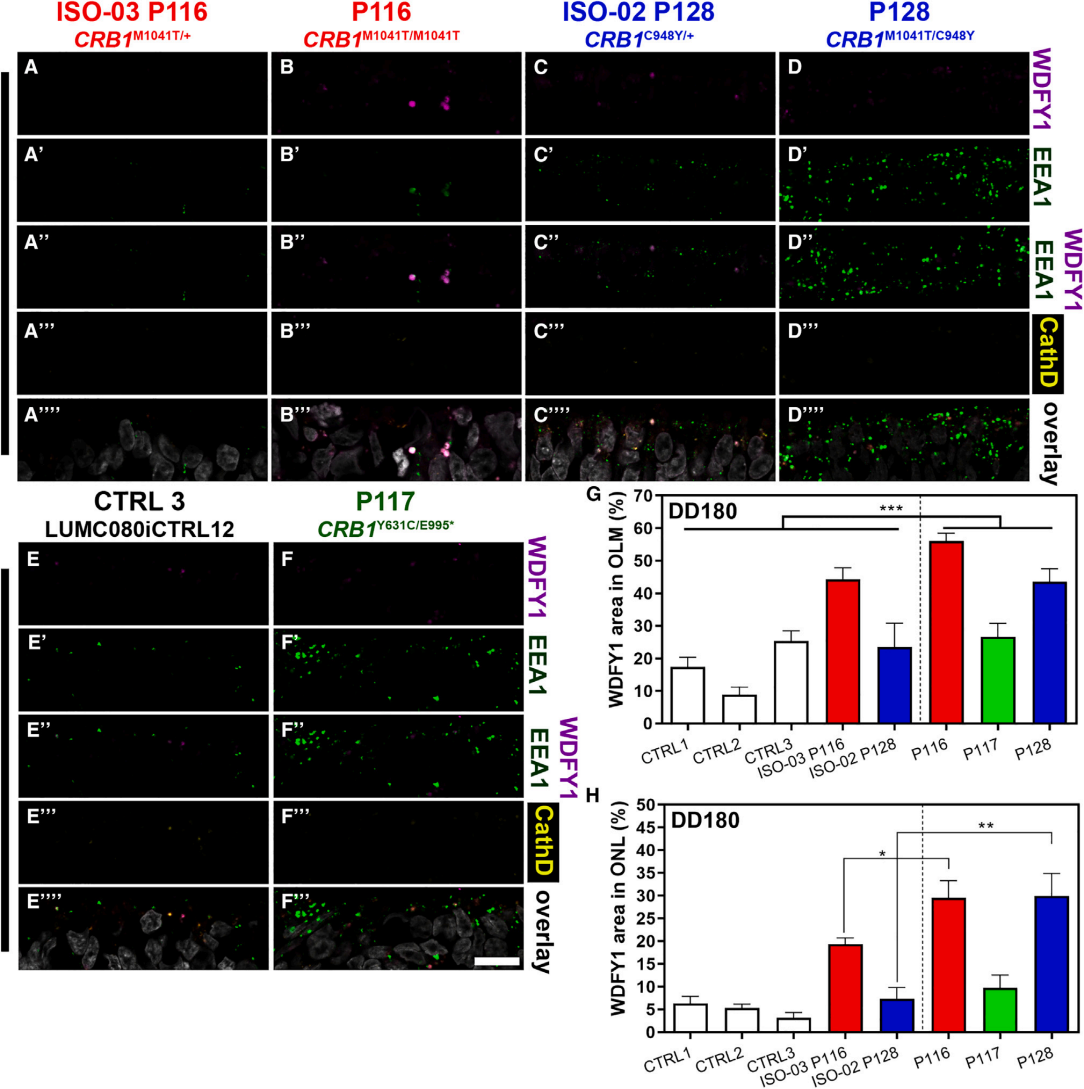
Increased levels of WDFY1 in EE s of patient *CRB1* patient retinal organoidsAll organoids are shown at DD180 (A–F) Immunofluorescence labeling of WDFY1 (magenta), EEA1 (green), and Cathepsin D (yellow). The overlay is shown in white (A‴–F‴). (G and H) Semiquantification of the fluorescence signal of WDFY1. (G) WDFY1 particle area/total OLM area in percent. (H) WDFY1 particle area/total ONL area in percent. Scale bars, 10 μm. Each data point in the graph represents individual organoids, of which an average was taken of 3 representative images. The SEM is derived from these averages. The numbers of individual organoids per condition (CTRL1, CTRL2, CTRL3, ISO-03 P116, ISO-02 P128, P116, P117, and P128) in (G) and (H) are 8, 9, 9, 10, 8, 10, 11, and 14, respectively (from at least two independent organoid batches). Statistical analysis: ^∗^p < 0.05, ^∗∗^p < 0.01, and ^∗∗∗^p < 0.001.

To determine where the decrease in autophagic flux originates, we investigated the expression of RAB11A. RAB11A is a small GTPase regulating formation of transport vesicles, recycling endosomes, and overall intracellular membrane trafficking (Kakar-Bhanot et al., 2019). The turnover of CRB and CRB membrane integration in the fruit fly is also determined by RAB11 (Aguilar-Aragon et al., 2020; Figure 1K). We found a strong signal of RAB11A co-labeling with F-actin in control organoids at the OLM (Figures 6A, 6C, and S7A–S7C). RAB11A/F-actin co-labeling was strongly reduced in *CRB1* RP patient retinal organoids (Figures 6B, 6D, and S7D), with RAB11A yielding 50% less signal at the OLM (Figure 6P). We further investigated the early endosomal population (EEA1+), late endolysosomal population (RAB7+), retromer-positive recycling compartments (RAB7+, VPS35+), and late endolysosomal population (RAB7+, ARL8A/B+). We found little difference in RAB7 expression between patient and control retinal organoids (Figures 6E–6H, 6I–6L, 6M, and S7E–S7H). However, less RAB7 co-labeled with VPS35 (Figures 6E‴–6H‴, and S7E‴–S7H‴), while co-labeling with ARL8A/B increased in patient retinal organoids (Figures 6 E″″–6H″″, and S7E″″–S7H″″). Furthermore, the EE population was increased in patient retinal organoids (Figures 6I′–6L′, 6N, and S7I). Finally, we investigated WDFY1 protein expression in retinal organoids, which we found to be upregulated at the transcript level in *Crb1*^KO^*Crb2*^ΔRPC^ compared with wild-type mouse retina (Figure 1J). Recently, WDFY1 was identified as part of the CUL4A-DDB1-WDFY1 E3 ubiquitin ligase complex involved in selective autophagy, called lysophagy (Teranishi et al., 2022). We found little co-labeling with EEs in control organoids (Figure 7). Moreover, WDFY1 does not necessarily have to reside on EEs but could instead be located in the cytosol, from where it is most likely scrapped by the phospholipid PtdIns(3)P, which is actively recruited by EEA1 (Gaullier et al., 1998; Ridley et al., 2001). We noted that EEs in patient *CRB1* organoids expressed more EEA1 (Figures 7B, 7D, and 7F) and partially co-localized with WDFY1/EEA1 (Figures 7B″, 7D″, and 7F″), whereas some of the WDFY1 co-localized with the lysosomal marker Cathepsin D (Figures 7B‴, 7D‴, and 7F‴), indicating that dysregulated endosomes may recruit more WDFY1. An increase in WDFY1 fluorescence signal was found in the ONL and OLM (Figures 7G and 7H). The statistical analysis of WDFY1 protein levels suggested no statistical significance when comparing the two *CRB1* patient retinal organoids combined with their corresponding isogenic controls. Interestingly, when comparing all of the controls (control-1, control-2, control-3, ISO-03-P116, and ISO-02-P128) with all *CRB1* retinal organoids (P116, P117, and P128), we measured a statistically significant difference (p < 0.001), suggesting an increase in levels of WDFY1 protein at the OLM and ONL.

## Discussion

It is still challenging to predict the progression of the spectrum of *CRB1*-associated retinal dystrophy phenotypes in *CRB1* patients based on the genotype variants. A thorough knowledge of the clinical spectrum, acquired through detailed phenotyping and natural history studies (Nguyen et al., 2022), is important for optimal counseling and patient selection for interventional studies. Here we address three common RP *CRB1*-associated variants on (1) their protein expression profile in the retina (significantly lowered levels of variant CRB1) and (2) the effects of the three CRB1 variants on apical EE maturation. Both results have implications for treatment (e.g., supporting EE maturation or *CRB* gene supplementation) and provide information for patients with one of the *CRB1* variants, resulting in a better understanding of the etiology.

We do find strong upregulation of *Wdfy1* in *Crb1*^*KO*^*Crb2*^*ΔRPC*^ compared with wild-type mice. The function of *Wdfy1* is still elusive in many cell types but has been linked to EEs (Ridley et al., 2001) involved in selective autophagy, termed lysophagy, recognizing and removing damaged lysosomes (Teranishi et al., 2022). Of interest is that the previously described *Crb1* KO mice as well as the *CRB1* patient retinal organoids described here have significantly reduced levels of but do not completely lack CRB proteins in MGCs. *Crb1* KO mice still have substantial levels of CRB2 in MGCs and photoreceptors, whereas *CRB1* patient retinal organoids still have substantial levels of CRB1 variant proteins plus CRB2 in MGCs and photoreceptors. In the scRNA-seq dataset on *CRB1* patient retinal organoids, *WDFY1* was not among the DEGs in MGCs or photoreceptors on DD230. However, significant changes in the endolysosomal system were detected that could be restored to isogenic control levels by overexpressing recombinant human CRB1 or CRB2 protein (Boon et al., 2023). Interestingly, we do detect increased levels of the early endosomal WDFY1 protein in *CRB1* patient retinal organoids compared with five independent control organoids. Future studies are needed to show the relevance of changed intracellular localization of WDFY1 in *CRB1* retinal organoids. Because WDFY1 has similar protein domains as those present in the endosomal protein EEA1, it is of interest to further study the potential link between decreased levels of variant CRB1 at the OLM and reductions in the levels of RAB11A and VPS35 as well as the increase in early endosomal WDFY1.

Retinal organoids express CRB1 and CRB2 protein at the OLM (Quinn et al., 2019a). The at least 4-fold reduced levels of CRB1 variant proteins in *CRB1* patient retinal organoids carrying missense mutations are not associated with reduced levels of *CRB1* mRNA transcript (Boon et al., 2023). In the scRNA-seq analysis, we find no differences in the level of expression of *CRB1* patient mRNA transcripts in MGCs or photoreceptors compared with the levels of *CRB1* mRNA transcript in corresponding isogenic controls. However, levels of *CRB2* transcripts in isogenic controls were lower in MGCs than in photoreceptors. Interestingly, scRNA-seq studies on cadaver human retina also reported a significantly (∼20-fold) lower level of *CRB2* mRNA transcripts in MGCs than in photoreceptors (https://www.proteinatlas.org/ENSG00000148204-CRB2/single+cell+type). In several mouse studies, it has been shown that CRB2 and CRB1 proteins have overlapping physiological functions in MGCs and that their loss in the retina results in similar phenotypes (Alves et al., 2013a; van de Pavert et al., 2004, 2007). We hypothesize that low levels of CRB2 in combination with low levels of variant CRB1 at the OLM of MGCs cause disruption of the endolysosomal system. There is an urgent need for a better understanding of the retinal phenotype of human retina with mutations in the *CRB1* gene, especially because variations in *CRB1* cause variable retinal phenotypes, including RP in young children as well as LCA in newborns. Interestingly, on diagnostic spectral-domain optical coherence tomography (SD-OCT) imaging of retinas of *CRB1* patients with LCA, the retina appears to be thickened. Such thickened retina is not observed in *CRB1* patients with late onset of RP or in our RP *CRB1* patient retinal organoids. Surprisingly, in patients, the same set of mutations can result in RP or LCA. In mice, onset of LCA or RP is dependent on the levels of CRB1 or CRB2 proteins in MGCs and photoreceptors (Boon et al., 2020; Buck et al., 2021). In mice, the levels of CRB1 and CRB2 proteins in MGCs were determined to be of near-equal functional relevance (Buck et al., 2021; Quinn et al., 2019b). The situation in the adult human retina might be different because the levels of *CRB2* mRNA transcripts in MGCs are ∼20-fold lower than in photoreceptors. In analogy to our findings in *Crb* mice, we hypothesize that the levels of human CRB2 functional protein units in MGCs might be substantially lower than the CRB1 functional protein units in MGCs and therefore cannot sufficiently compensate when CRB1 variant protein levels are reduced in *CRB1* retina. Lowering the levels of CRB1 variant proteins in MGCs of *CRB1* patients might cause a shortage of CRB proteins at the OLM in MGCs that cannot be compensated for by the relatively low levels of CRB2 proteins in MGCs. One result of lowered CRB proteins might be disturbances in the RAB11A recycling of several apical plasma membrane proteins close to the OLM, here exemplified by lower levels of NOTCH1 concomitant with the lower levels of variant CRB1.

It remains to be determined what controls CRB1 protein expression, localization, and turnover rate at the OLM to determine the progression of retinal degeneration in *CRB1*-associated retinal dystrophy phenotypes in *CRB1* patients. In the present study, we focused on the endolysosomal system because we found an increase in endosome-associated *Wdfy1* gene expression in *Crb1*^*KO*^*Crb2*^*ΔRPC*^ mice and degradation-associated proteins on endolysosomes (ARL8/LAMP1/p62/LC3 puncta) in patient retinal organoids. Besides western blotting for LC3-I and LC3-II, we also tried western blotting for anti-CRB1 and anti-CRB2 and against endosomal proteins. Unfortunately, whereas the total retinal organoid lysates showed LC3B and recoverin protein bands on western blots, we could not purify MGCs or photoreceptors from the limited number of isogenic and *CRB1* retinal organoids to detect CRB1, CRB2, NOTCH1, or endosomal proteins on western blots with the available antibodies.

We paid particular attention to the retromer-positive and early recycling compartments. The retromer complex (SNX1/2, SNX5/6, VPS26, VPS29, and VPS35) on early/late endosomes and the budded off vesicles/recycling endosomes (e.g., RAB11A, TfR1) work in synergy for efficient protein recycling in cells (Trousdale and Kim, 2015) and are thus potentially essential parameters controlling CRB1 protein turnover. What is more, the retromer proteins VPS26/VPS35 localize on early and late endosomes in fruit fly photoreceptors, and loss of VPS26 or VPS35 considerably increases degradative compartments (Wang et al., 2014). Recycling of CRB2 at the apical membrane is determined by binding of its ICD to VPS35 of the retromer complex on EEs (Pocha et al., 2011). CRB protein recycling also depends on RAB11 (Aguilar-Aragon et al., 2020). The ICD of CRB2A on RAB11A+ recycling endosomes takes part in regulating the cell cycle exit of RPCs and maintaining NOTCH1 at the OLM (Clark et al., 2020). RAB11 is known to mediate release of the early endosomal cargo (e.g., NOTCH1, CRB1) to the endocytic recycling compartment when peripheral EEs experience a temporary peak in PtdIns(3)P integration on their vesicular membrane (Campa et al., 2018; Gaullier et al., 1998). Potentially poor RAB11A-initiated release in *CRB1* patient retinal organoids may also amass WDFY1 mediated by its FYVE domain binding to PtdIns(3)P (Ridley et al., 2001). The presence of PtdIns(3)P in the membrane is vital for shifting WDFY1 from a soluble and cytosolic form to an EE or vesicle membrane-bound form. The actual function and normal equilibrium of cytosolic versus membrane-bound WDFY1 are not known. In line with this, our data on *CRB1* variant patient retinal organoids show reduced retromer-associated late endosomes (VSP35+/RAB7+), an increase in EEs destined for degradation (EEA1+/WDFY1+), a decrease in recycling endosomes (RAB11A+), and concomitant expansion of degradative endolysosomal compartments (ARL8/LAMP1/LC3-II/p62+). We propose that, in *CRB1* patient retinal organoids, disturbed EEs are trafficked to degradative compartments, lowering CRB1 protein expression at the OLM.

## Experimental procedures

### Resource availability

#### Corresponding author

Further information and requests for resources and reagents should be directed to and will be fulfilled by the corresponding author, Jan Wijnholds (j.wijnholds@lumc.nl).

#### Materials availability

Materials and additional details can be made available by the corresponding author upon reasonable request.

#### Data and code availability

RNA-seq data are available at the NCBI Gene Expression Omnibus database (GEO: GSE239456).

### Animals

Procedures concerning animals were performed with permission of the ethics committee of Leiden University Medical Center and the animal experimentation committee of the Royal Netherlands Academy of Arts and Sciences (KNAW) under permit NIN 12.105.

### RNA-seq

Sequencing was performed using Life Technologies SOLiD5500 with single-end, 50-bp reads. Two separate runs were performed. Run 1 contained 30 mice; n = 5 *Crb1*^KO^*Crb2*^F/F^ (*Crb1*^KO^) and n = 5 *Crb1*^KO^*Crb2*^ΔRPC^ (Tg) per development time point of the mouse embryo at embryonic day 15.5 (E15.5), E17.5, and P1. Run 2 contained 9 mice; n = 4 *Crb1*^KO^*Crb2*^ΔRPC^ mice (C57BL/6JOlaHsd) and n = 5 healthy mice (C57BL/6JRccHsd = wild type [WT]) at E15.5. Reads were aligned against mm10 using the “whole.transcriptome.frag” workflow with the bamgen.mqv.threshold set to 20 (Lifescope v.2.5). Counts were obtained using the gene transfer format (GTF) output format as supplied by Lifescope (transformation of refGene.txt downloaded from the University of California Santa Cruz [UCSC] Table Browser on June 25, 2014) within this workflow.

### Differential expression analysis

The analysis was performed as described previously (Pellissier et al., 2013).

### Cell culture

We previously described the three male *CRB1* RP-patient human iPSC (hiPSC) lines (patient lines 1–3: LUMC0116iCRB09 or P116, LUMC0117iCRB01 or P117, LUMC0128iCRB01 or P128) and three control lines (CTRL 1–3: LUMC0004iCTRL10 (hPSC^reg^ name: LUMCi029-B), LUMC0044iCTRL44, LUMC0080iCTRL12; Table S1; (Quinn et al., 2019a). Testing for pluripotency of hiPSC lines was performed for at least 3 clones per mutated line or isogenic CTRLs (Figures S3C, S3F, and S3G; see Table S2 for antibody concentrations).

### CRISPR-Cas9-based gene repair of the hiPSC lines LUMC0116iCRB09 and LUMC0128iCRB01

The *CRB1* variant c.3122T>C (exon 9) was repaired by CRISPR-Cas9 ribonucleoprotein (RNP)-mediated homologous recombination. Sequences of the single guide RNA (sgRNA) and the repair template single-stranded oligodeoxynucleotides (ssODN) are provided in Figures S3D and S3E.

### Retinal organoid differentiation

The differentiation of the hiPSCs is described in the supplemental information.

### Fluorescence quantification in regions of interest (ROIs)

All organoids (7 μm sectioned) imaged for fluorescence semiquantification were stained (Table S2) with the same antibody mix at the same time, imaged in one confocal microscopy session, and included a negative CTRL (no primary antibody added; see supplemental information).

### Conjugation of NOTCH1 and CRB1 antibody to plus and minus oligonucleotide probes

We used the Duolink Probemaker Set (Sigma-Aldrich) to conjugate two same-species antibodies (mouse anti-NOTCH1 and mouse anti-CRB1 ECD antibodies) to the plus or minus oligonucleotide probes as described in the protocol.

### PLA

For the PLA, the Duolink *In Situ* Detection Reagents Kit Green (Sigma-Aldrich) was used. Slides with sliced organoids were washed, and the tissue slices were circled with a hydrophobic pen. Slides were blocked with one drop of blocking solution from the Duolink PLA probe set per tissue slice and incubated for 1 h at 37°C in a humidity chamber. Blocking buffer was tapped of, and the conjugated antibodies diluted in PLA probe diluent from the PLA probe kit were added for overnight incubation at 4°C and processed as described in the supplemental information.

### Statistical analysis

The organoids were acquired from two or more differentiations, and we aimed to obtain 3 images per organoid. Each image was analyzed for retinal length, thickness, total cells, and total positive retinal cell population markers with ImageJ. Data were normalized per 100 μm of retinal length. For statistical analysis, GraphPad Prism v.8 was used. Shown values are expressed as mean ± standard error of the mean (SEM). Quantifications were tested for normality, and when distributed normally, unpaired t tests assuming equal variance were used to compare patient and CTRL lines. Measurements that did not show a normal distribution were tested with a Mann-Whitney test. Significance is indicated in graphs as ^∗^p < 0.05, ^∗∗^p < 0.01, and ^∗∗∗^p < 0.001.

## Author contributions

Conceptualization, T.M.B., P.M.J.Q., L.P.P., I.B. and J.W.; methodology, retinal organoids and mouse morphology, T.M.B., P.M.J.Q., L.P.P., A.A.M., and D.K.; patient hiPSC line repair (isogenic controls) and characterization, C.H.A., C.F., H.M.M.M., N.B., and X.L.; formal analysis/data curation of the RNA-seq dataset, A.J., A.J.K., F.B., P.M.J.Q., and J.W.; formal analysis, retinal organoids and mouse morphology, T.M.B., P.M.J.Q., L.P.P., X.L., and N.B.; investigation, T.M.B., P.M.J.Q., L.P.P., X.L., N.B., and J.W.; writing – original draft, T.M.B. and J.W.; writing – review & editing, all authors; visualization, T.M.B., P.M.J.Q., L.P.P., and J.W.; resources, I.B. and J.N.; supervision, J.W.; funding acquisition, T.M.B. and J.W.

## Supplemental information

Document S1. Figures S1–S7, Tables S1–S6, and Supplemental experimental procedures

Document S2. Article plus supplemental information

## References

[bib1] Aguilar-Aragon M., Fletcher G., Thompson B.J. (2020). The cytoskeletal motor proteins Dynein and MyoV direct apical transport of Crumbs. Dev. Biol..

[bib42] Alves C.H., Sanz A.S., Park B., Pellissier L.P., Tanimoto N., Beck S.C., Huber G., Murtaza M., Richard F., Sridevi Gurubaran I. (2013). Loss of CRB2 in the mouse retina mimics human retinitis pigmentosa due to mutations in the CRB1 gene. Hum. Mol. Genet..

[bib2] Alves C.H., Bossers K., Vos R.M., Essing A.H.W., Swagemakers S., van der Spek P.J., Verhaagen J., Wijnholds J. (2013). Microarray and morphological analysis of early postnatal CRB2 mutant retinas on a pure C57BL/6J genetic background. PLoS One.

[bib3] Boon N., Lu X., Andriessen C.A., Moustakas I., Buck T.M., Freund C., Arendzen C.H., Böhringer S., Mei H., Wijnholds J. (2023). AAV-mediated gene augmentation therapy of CRB1 patient-derived retinal organoids restores the histological and transcriptional retinal phenotype. Stem Cell Rep..

[bib44] Boon N., Wijnholds J., Pellissier L.P. (2020). Research models and gene augmentation therapy for CRB1 retinal dystrophies. Front. Neurosci..

[bib4] Buck T.M., Vos R.M., Alves C.H., Wijnholds J. (2021). AAV-CRB2 protects against vision loss in an inducible CRB1 retinitis pigmentosa mouse model. Mol. Ther. Methods Clin. Dev..

[bib5] Campa C.C., Margaria J.P., Derle A., Del Giudice M., De Santis M.C., Gozzelino L., Copperi F., Bosia C., Hirsch E. (2018). Rab11 activity and PtdIns(3)P turnover removes recycling cargo from endosomes. Nat. Chem. Biol..

[bib6] Chen X., Emerson M.M. (2021). Notch signaling represses cone photoreceptor formation through the regulation of retinal progenitor cell states. Sci. Rep..

[bib7] Clark B.S., Miesfeld J.B., Flinn M.A., Collery R.F., Link B.A. (2020). Dynamic polarization of Rab11a modulates Crb2a localization and impacts signaling to regulate retinal neurogenesis. Front. Cell Dev. Biol..

[bib8] Farfel-Becker T., Roney J.C., Cheng X.T., Li S., Cuddy S.R., Sheng Z.H. (2019). Neuronal soma-derived degradative lysosomes are continuously delivered to distal axons to maintain local degradation capacity. Cell Rep..

[bib9] Gajovic S., Mitrecic D., Augustincic L., Iaconcig A., Muro A.F. (2006). Unexpected rescue of alpha-synuclein and multimerin1 deletion in C57BL/6JOlaHsd mice by beta-adducin knockout. Transgenic Res..

[bib10] Gaullier J.M., Simonsen A., D'Arrigo A., Bremnes B., Stenmark H., Aasland R. (1998). FYVE fingers bind PtdIns(3)P. Nature.

[bib11] Ha T., Moon K.H., Dai L., Hatakeyama J., Yoon K., Park H.S., Kong Y.Y., Shimamura K., Kim J.W. (2017). The retinal pigment epithelium is a notch signaling niche in the mouse retina. Cell Rep..

[bib12] Herranz H., Stamataki E., Feiguin F., Milán M. (2006). Self-refinement of Notch activity through the transmembrane protein Crumbs: modulation of gamma-secretase activity. EMBO Rep..

[bib13] Kakar-Bhanot R., Brahmbhatt K., Chauhan B., Katkam R.R., Bashir T., Gawde H., Mayadeo N., Chaudhari U.K., Sachdeva G. (2019). Rab11a drives adhesion molecules to the surface of endometrial epithelial cells. Hum. Reprod..

[bib14] Kraut R.S., Knust E. (2019). Changes in endolysosomal organization define a pre-degenerative state in the crumbs mutant Drosophila retina. PLoS One.

[bib15] Laprise P. (2011). Emerging role for epithelial polarity proteins of the Crumbs family as potential tumor suppressors. J. Biomed. Biotechnol..

[bib16] Lattner J., Leng W., Knust E., Brankatschk M., Flores-Benitez D. (2019). Crumbs organizes the transport machinery by regulating apical levels of PI(4,5)P(2) in Drosophila. Elife.

[bib17] Nemetschke L., Knust E. (2016). Drosophila Crumbs prevents ectopic Notch activation in developing wings by inhibiting ligand-independent endocytosis. Development.

[bib18] Nguyen X.T.A., Talib M., van Schooneveld M.J., Wijnholds J., van Genderen M.M., Schalij-Delfos N.E., Klaver C.C.W., Talsma H.E., Fiocco M., Florijn R.J. (2022). CRB1-associated retinal dystrophies: a prospective natural history study in anticipation of future clinical trials. Am. J. Ophthalmol..

[bib19] Ohata S., Aoki R., Kinoshita S., Yamaguchi M., Tsuruoka-Kinoshita S., Tanaka H., Wada H., Watabe S., Tsuboi T., Masai I., Okamoto H. (2011). Dual roles of Notch in regulation of apically restricted mitosis and apicobasal polarity of neuroepithelial cells. Neuron.

[bib20] Overhoff M., De Bruyckere E., Kononenko N.L. (2021). Mechanisms of neuronal survival safeguarded by endocytosis and autophagy. J. Neurochem..

[bib21] Pellissier L.P., Alves C.H., Quinn P.M., Vos R.M., Tanimoto N., Lundvig D.M.S., Dudok J.J., Hooibrink B., Richard F., Beck S.C. (2013). Targeted ablation of CRB1 and CRB2 in retinal progenitor cells mimics Leber congenital amaurosis. PLoS Genet..

[bib22] Pellissier L.P., Lundvig D.M.S., Tanimoto N., Klooster J., Vos R.M., Richard F., Sothilingam V., Garcia Garrido M., Le Bivic A., Seeliger M.W., Wijnholds J. (2014). CRB2 acts as a modifying factor of CRB1-related retinal dystrophies in mice. Hum. Mol. Genet..

[bib41] Pellissier L.P., Quinn P.M., Alves C.H., Vos R.M., Klooster J., Flannery J.G., Heimel J.A., Wijnholds J. (2015). Gene therapy into photoreceptors and Müller glial cells restores retinal structure and function in CRB1 retinitis pigmentosa mouse models. Hum. Mol. Genet..

[bib23] Pocha S.M., Wassmer T., Niehage C., Hoflack B., Knust E. (2011). Retromer controls epithelial cell polarity by trafficking the apical determinant Crumbs. Curr. Biol..

[bib24] Pradhan J., Noakes P.G., Bellingham M.C. (2019). The Role of altered BDNF/TrkB signaling in amyotrophic lateral sclerosis. Front. Cell. Neurosci..

[bib25] Quinn P.M., Alves C.H., Klooster J., Wijnholds J. (2018). CRB2 in immature photoreceptors determines the superior-inferior symmetry of the developing retina to maintain retinal structure and function. Hum. Mol. Genet..

[bib26] Quinn P.M., Buck T.M., Mulder A.A., Ohonin C., Alves C.H., Vos R.M., Bialecka M., van Herwaarden T., van Dijk E.H.C., Talib M. (2019). Human iPSC-derived retinas recapitulate the fetal CRB1 CRB2 complex formation and demonstrate that photoreceptors and muller glia are targets of AAV5. Stem Cell Rep..

[bib27] Quinn P.M., Mulder A.A., Henrique Alves C., Desrosiers M., de Vries S.I., Klooster J., Dalkara D., Koster A.J., Jost C.R., Wijnholds J. (2019). Loss of CRB2 in Muller glial cells modifies a CRB1-associated retinitis pigmentosa phenotype into a Leber congenital amaurosis phenotype. Hum. Mol. Genet..

[bib28] Richardson E.C.N., Pichaud F. (2010). Crumbs is required to achieve proper organ size control during Drosophila head development. Development.

[bib29] Ridley S.H., Ktistakis N., Davidson K., Anderson K.E., Manifava M., Ellson C.D., Lipp P., Bootman M., Coadwell J., Nazarian A. (2001). FENS-1 and DFCP1 are FYVE domain-containing proteins with distinct functions in the endosomal and Golgi compartments. J. Cell Sci..

[bib30] Rosa-Ferreira C., Munro S. (2011). Arl8 and SKIP act together to link lysosomes to kinesin-1. Dev. Cell.

[bib31] Teranishi H., Tabata K., Saeki M., Umemoto T., Hatta T., Otomo T., Yamamoto K., Natsume T., Yoshimori T., Hamasaki M. (2022). Identification of CUL4A-DDB1-WDFY1 as an E3 ubiquitin ligase complex involved in initiation of lysophagy. Cell Rep..

[bib32] Thompson B.J., Pichaud F., Röper K. (2013). Sticking together the Crumbs - an unexpected function for an old friend. Nat. Rev. Mol. Cell Biol..

[bib33] Trousdale C., Kim K. (2015). Retromer: structure, function, and roles in mammalian disease. Eur. J. Cell Biol..

[bib43] van de Pavert S.A., Kantardzhieva A., Malysheva A., Meuleman J., Versteeg I., Levelt C., Klooster J., Geiger S., Seeliger M.W., Rashbass P. (2004). Crumbs homologue 1 is required for maintenance of photoreceptor cell polarization and adhesion during light exposure. J. Cell Sci..

[bib34] van de Pavert S.A., Sanz A.S., Aartsen W.M., Vos R.M., Versteeg I., Beck S.C., Klooster J., Seeliger M.W., Wijnholds J. (2007). Crb1 is a determinant of retinal apical Muller glia cell features. Glia.

[bib39] van Rossum A.G., Aartsen W.M., Meuleman J., Klooster J., Malysheva A., Versteeg I., Arsanto J.P., Le Bivic A., Wijnholds J. (2006). Pals1/Mpp5 is required for correct localization of Crb1 at the subapical region in polarized Mueller glia cells. Hum. Mol. Genet..

[bib35] Wang S., Tan K.L., Agosto M.A., Xiong B., Yamamoto S., Sandoval H., Jaiswal M., Bayat V., Zhang K., Charng W.L. (2014). The retromer complex is required for rhodopsin recycling and its loss leads to photoreceptor degeneration. PLoS Biol..

[bib36] Wooten M.W., Geetha T., Babu J.R., Seibenhener M.L., Peng J., Cox N., Diaz-Meco M.T., Moscat J. (2008). Essential role of sequestosome 1/p62 in regulating accumulation of Lys63-ubiquitinated proteins. J. Biol. Chem..

[bib37] Zhou B., Wu Y., Lin X. (2011). Retromer regulates apical-basal polarity through recycling Crumbs. Dev. Biol..

[bib38] Zou J., Wang X., Wei X. (2012). Crb apical polarity proteins maintain zebrafish retinal cone mosaics via intercellular binding of their extracellular domains. Dev. Cell.

