# Supplementary material for: CRB1 is required for recycling by RAB11A+ vesicles in human retinal organoids
Source: Stem Cell Reports. 2023 Aug 3;18(9):1793–810. doi: 10.1016/j.stemcr.2023.07.001 (PMC10545476; doi:10.1016/j.stemcr.2023.07.001)
Supplement: Document S1. Figures S1–S7, Tables S1–S6, and Supplemental experimental procedures [file mmc1.pdf]

Stem Cell Reports, Volume *18*

Supplemental Information

CRB1 is required for recycling by RAB11A+ vesicles in human retinal organoids

Thilo M. Buck, Peter M.J. Quinn, Lucie P. Pellissier, Aat A. Mulder, Aldo Jongejan, Xuefei Lu, Nanda Boon, Daniëlle Koot, Hind Almushattat, Christiaan H. Arendzen, Rogier M. Vos, Edward J. Bradley, Christian Freund, Harald M.M. Mikkers, Camiel J.F. Boon, Perry D. Moerland, Frank Baas, Abraham J. Koster, Jacques Neefjes, Ilana Berlin, Carolina R. Jost, and Jan Wijnholds

Supplementary data related to manuscript:
CRB1 is required for recycling by RAB11A+ vesicles in human retinal organoids.
Buck TM *et al.*, 2023

Overview

Supplemental Figures and Legends

Figure S1. The retinal morphological phenotype is milder in *Crb1*^{KO}*Crb2*$^{\Delta RPC}$ on 100% C57/B6 genetic background than on 50% mixed genetic background. Related to Figure 1.
Figure S2. The *Crb1*^{KO}*Crb2*$^{\Delta RPC}$ mouse displays an LCA-like *CRB1* phenotype. Related to Figure 1.
Figure S3. Validation of the isogenic control iPS cell lines. Related to Figure 3-7.
Figure S4. The onset of CRB1 expression in control organoids. Related to Figure 4.
Figure S5. More lysosomes are present in *CRB1* patient retinal organoids. Related to Figure 4-5.
Figure S6. Increased number of degradative vesicles/compartments in *CRB1* patient retinal organoids. Related to Figure 5.
Figure S7. Dysregulation of the endolysosomal system in *CRB1* patient retinal organoids. Related to Figure 6.
Table S1. Information on hiPSC lines. Related to Figure 2-6.
Table S2: Antibody list and dilution used for immunohistochemistry and western blots. Related to all figures.
Table S3. ISO-02-P116 top 10 off-target candidates. Related to figures 3-7.
Table S4. ISO-03-P116 top 10 off-target candidates. Related to figures 3-7.
Table S5. ISO-02 P128 top 10 off-target candidates. Related to figures 3-7.
Table S6. Karyotyping of hiPS cell lines. Related to figures 2-7.

Supplemental Experimental Procedures

Supplemental Information

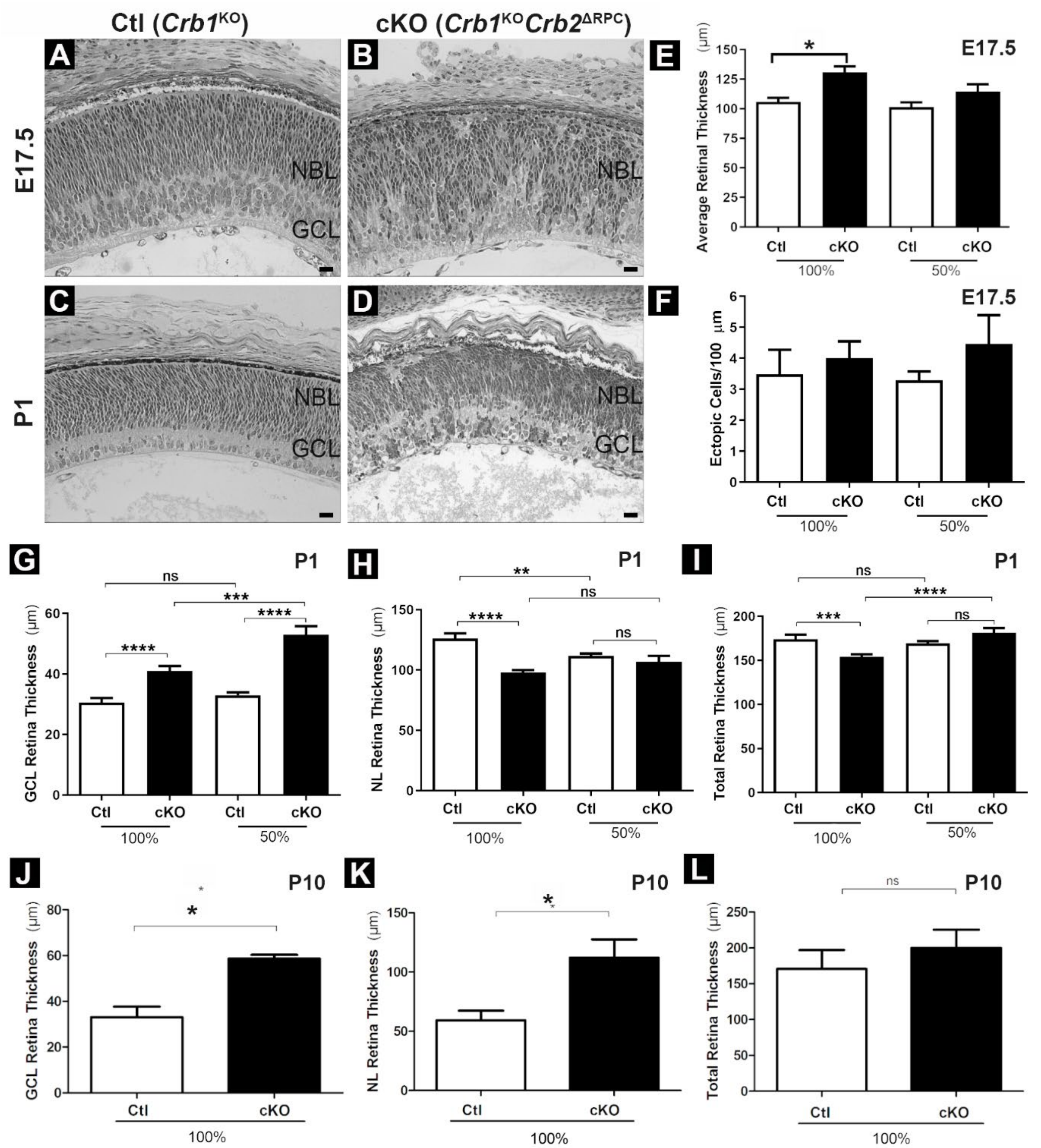

Figure S1. The retinal morphological phenotype is milder in *Crb1*^{KO}*Crb2*$^{\Delta RPC}$ on 100% C57/B6 genetic background than on 50% mixed genetic background. (A-D) Retinal morphology on plastic sections of Ctl (*Crb1*^{KO}) and cKO (*Crb1*^{KO}*Crb2*$^{\Delta RPC}$) mice on 100% C57/B6 genetic background at E17.5 and P1. (E-F) Ctl (*Crb1*^{KO}) and cKO (*Crb1*^{KO}*Crb2*$^{\Delta RPC}$) mice on 50% and 100% C57/B6 genetic background. (E-F) Average retinal thickness and ectopic nuclei per 100 µm retinal length at E17.5. (G-I) Ganglion cell layer (GCL) thickness, neuroretina layer (NL) thickness, and retinal thickness (OLM-ILM) at P1. (J-L) GCL thickness, NL thickness, and retinal thickness in 100% C57/B6 genetic background at P10. Scale bar, 20 µm. $p<0.05$ (*), $p<0.01$ (**), and $p<0.001$ (***). Related to Figure 1.

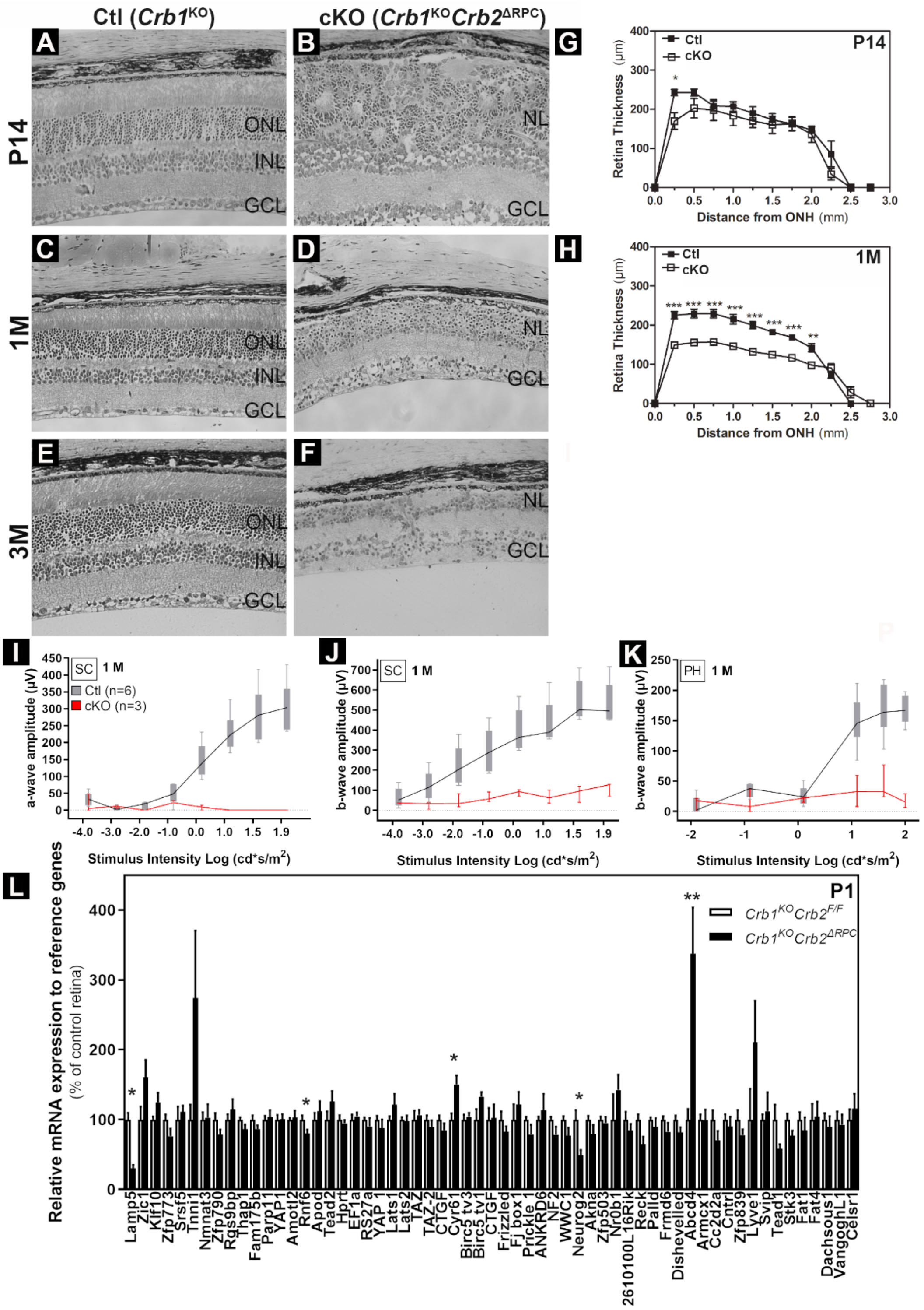

Figure S2. The *Crb1*^{KO}*Crb2*$^{\Delta RPC}$ mouse displays an LCA-like *CRB1* phenotype. (A-F) Retinal morphology on plastic sections of Ctl (*Crb1*^{KO}) and cKO (*Crb1*^{KO}*Crb2*$^{\Delta RPC}$) mice on 100% C57/B6 genetic background at P14, 1M, and 3M. (G-H) Retinal thickness (OLM-ILM) at P14, 1M, and 3M. (I-K) Electroretinographic (ERG) analysis of the retinal function at 1M. Conditional KOs are in red and Ctl age-matched littermates in gray: (L-M) Scotopic (SC) single-flash intensity series (-4, -3 , -2, -1, 0, 1, 1.5, 1.9 log cd s/m² light intensity). (I) scotopic a-wave amplitudes. (J) scotopic b-wave amplitudes. (K) Photopic (PH) single-flash ERG at different light intensities (-2, -1, 0, 1, 1.5, 1.9 log cd s/m² light intensity at 30 cd/m² background light). Boxes indicate the 25 and 75% quantile range, whiskers indicate the 5 and 95% quantiles, and the intersection of line and error bar indicates the median of the data. (L) *Crb1*^{KO}*Crb2*$^{\Delta RPC}$ vs *Crb1*^{KO} retina qPCR validation (n=5 retinas / group) of transcript changes at P1. Statistical significance calculated by a two-sided T-Test (p<0.05; 0.01; 0.001 shown as *, **, and ***). Scale bar, 20 µm. Related to Figure 1.

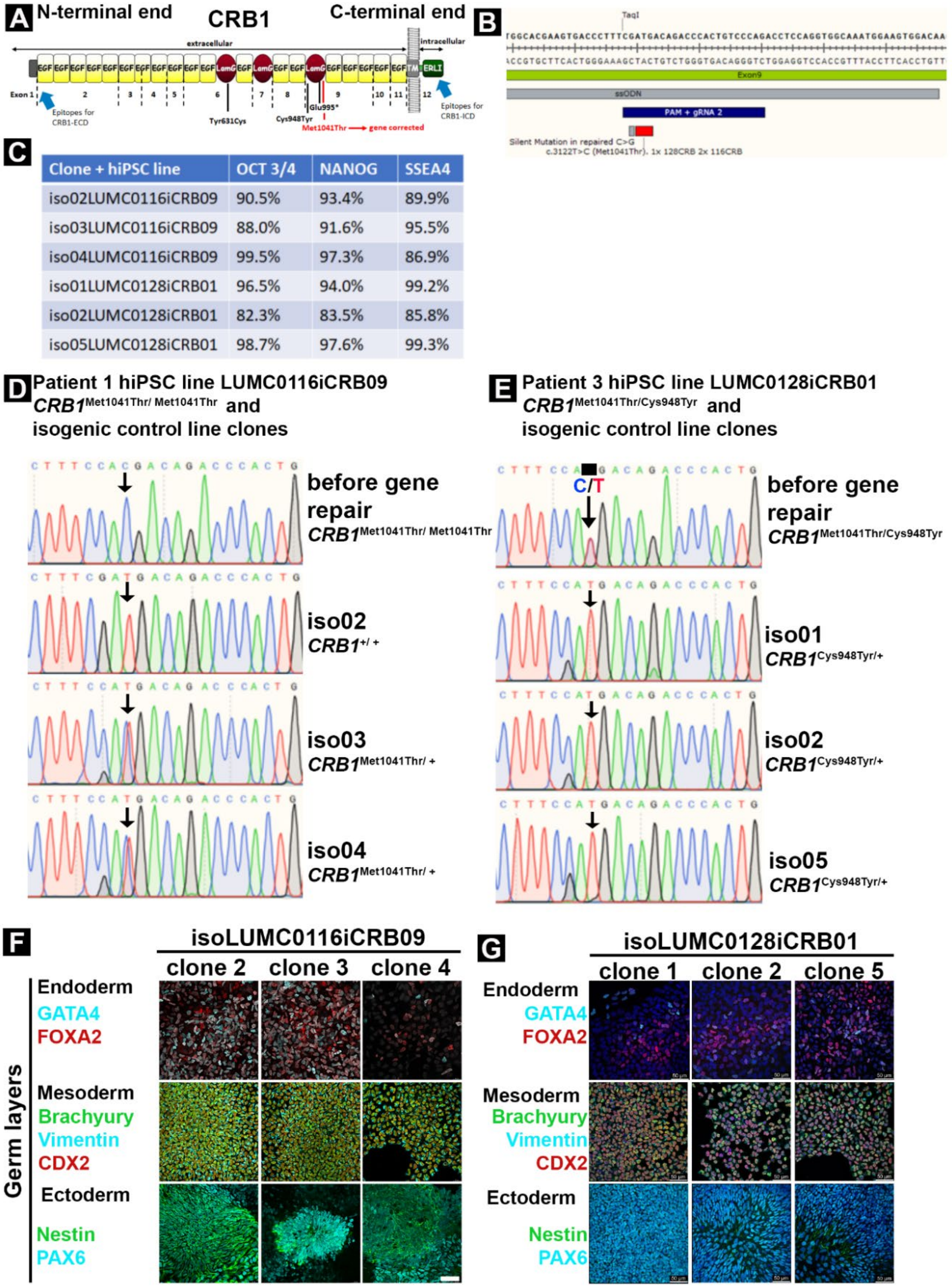

Clone + hiPSC line	OCT 3/4	NANOG	SSEA4
iso02LUMC0116iCRB09	90.5%	93.4%	89.9%
iso03LUMC0116iCRB09	88.0%	91.6%	95.5%
iso04LUMC0116iCRB09	99.5%	97.3%	86.9%
iso01LUMC0128iCRB01	96.5%	94.0%	99.2%
iso02LUMC0128iCRB01	82.3%	83.5%	85.8%
iso05LUMC0128iCRB01	98.7%	97.6%	99.3%

Figure S3. Validation of the isogenic control iPS cell lines. (A) CRB1 protein domains and the corresponding exons, CRB1 patient variants, and the epitope of the CRB1-ICD and CRB1-ECD antibodies. (B) Gene correction by single-stranded oligodeoxynucleotides (ssODN), gRNA, and nuclease Cas9 on locus c.3122T>C. (C) Flow cytometry sorted human iPSC clones indicating expression of pluripotency markers SSEA4, OCT3/4, and NANOG. (D-E) Sanger sequence validation of 3 clones per patient line. (F-G) human iPSC clones can be differentiated in to the three germ layers. Scale bar, 50 µm. Related to Figure 3-7.

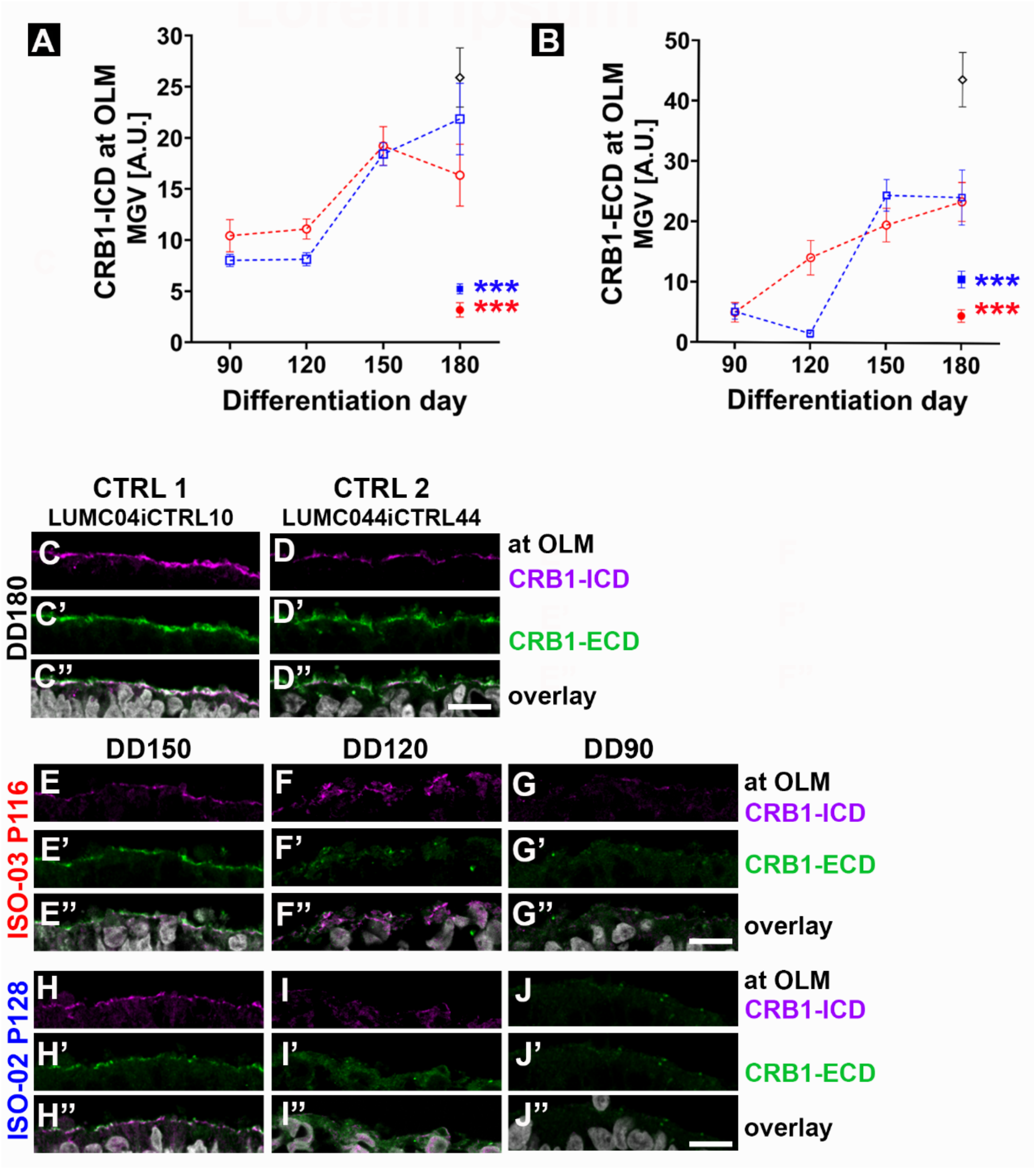

Figure S4. The onset of CRB1 expression in control organoids. (A-B) CRB1-Alexa conjugated fluorescence signal detected over time on ICD-(A) and ECD-(B) CRB1 antibodies (empty circles/dashed lines, isogenic controls; filled circles, patient lines; black circle, CTRL's; DD180, n=8; DD90-150, n=4-6 organoids per line). (C-G) CRB1 stained with an (C-J) intracellular (ICD) or (C'-G') extracellular domain (ECD) epitope antibodies at the OLM, and (C''-G'') overlay (scale bar, 10 µm) at DD180 (C-D; CTRL 1+2), DD150 (E+H; isogenic ISO-03 P116 and ISO-P128), DD120 (F+I; isogenic ISO-03 P116 and ISO-P128), and DD90 (G+J; isogenic ISO-03 P116 and ISO-P128). OLM, outer limiting membrane; ICD, intracellular domain; ECD, extracellular domain; DD, differentiation day. Each datapoint in the graphs A and B represents individual organoids, of which an average has been taken of 3 representative images. The standard error of mean (SEM) is derived from these averages. The number of individual organoids per time point (DD90; DD120; DD150; DD180) for condition ISO-03 P116 is n=6; 5; 5; 8; for ISO-02 P128 is n= 6; 6; 6; 6; for P116 (DD180) n=8; for P128 (DD180) n=8; and for CTRL1 (DD180) n=8. From at least two independent organoid batches. Statistical analysis: $p < 0.001$(***). Related to Figure 4.

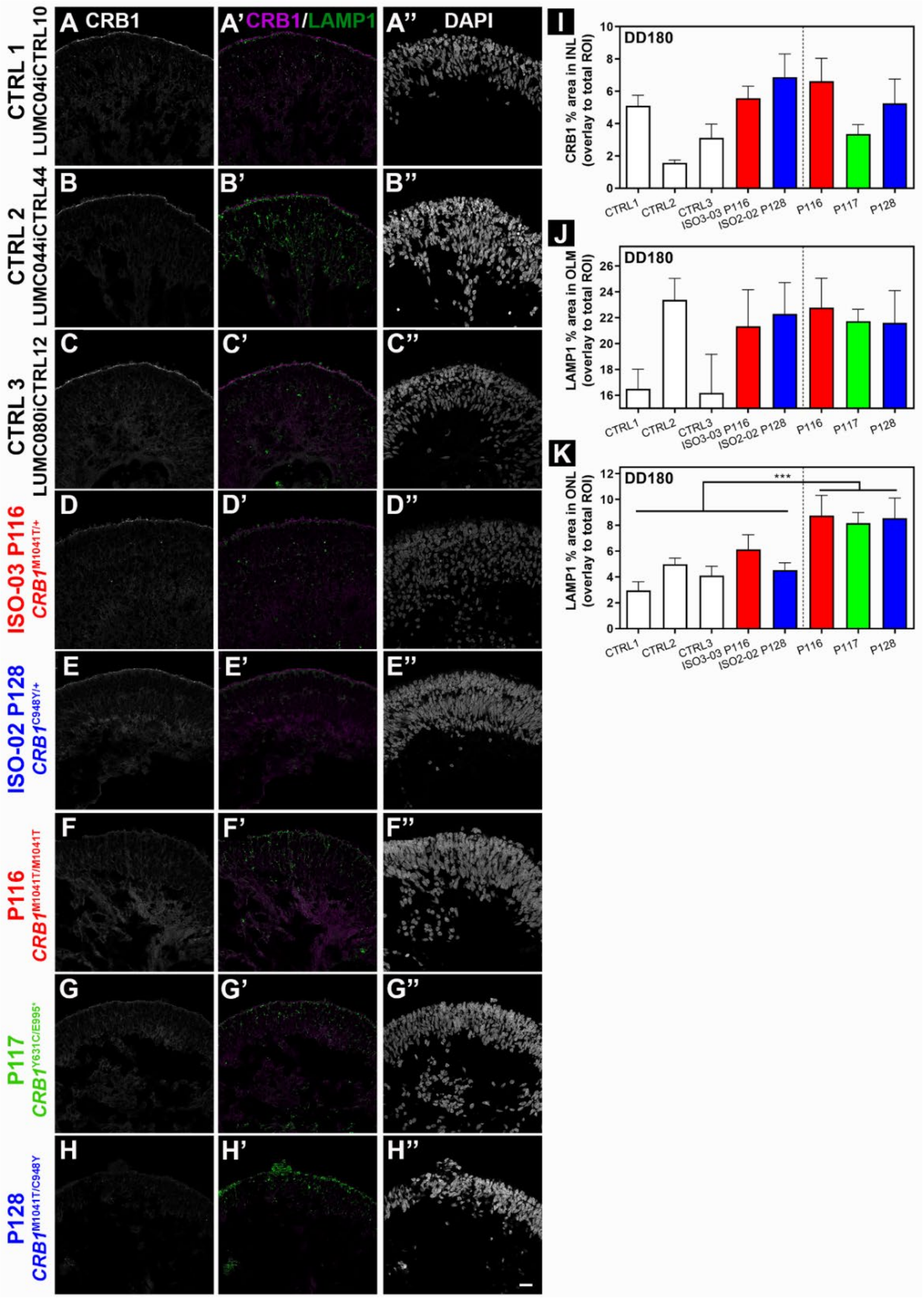

Figure S5. More lysosomes are present in CRB1 patient retinal organoids. Immunofluorescence CRB1 puncta (A-H; grayscale), co-labelling CRB1/LAMP1 (green/magenta in A'-H'), and nuclei (grayscale in A''-H'') in *CRB1* patient retinal organoids at DD180. (A-H) patient CRB1 retinal organoids express little CRB1 protein throughout the retina. (A'-H') Little co-labelling of CRB1 (magenta) and lysosomes (LAMP1+, green) were found. (A'-H') Patient CRB1 retinal organoids have more lysosomes at the ONL. (A''-H'') Nuclei staining (DAPI, grayscale) showing overall morphology. (I) CRB1-fluorescence signal measured by puncta in the INL. (J-K) LAMP1+ puncta measured in the OLM and ONL. Scale bar, 50 µm. Each datapoint in the graph represents individual organoids, of which an average has been taken of 3 representative images. The standard error of mean (SEM) is derived from these averages. The number of individual organoids per condition (CTRL1; CTRL2; CTRL3; ISO-03 P116; ISO-02 P128; P116; P117; P128) in (I-K) is n=9; 9; 9; 10; 10; 12; 9; 13. From at least two independent organoid batches. Statistical analysis: $p < 0.001$(***). Related to Figure 4-5.

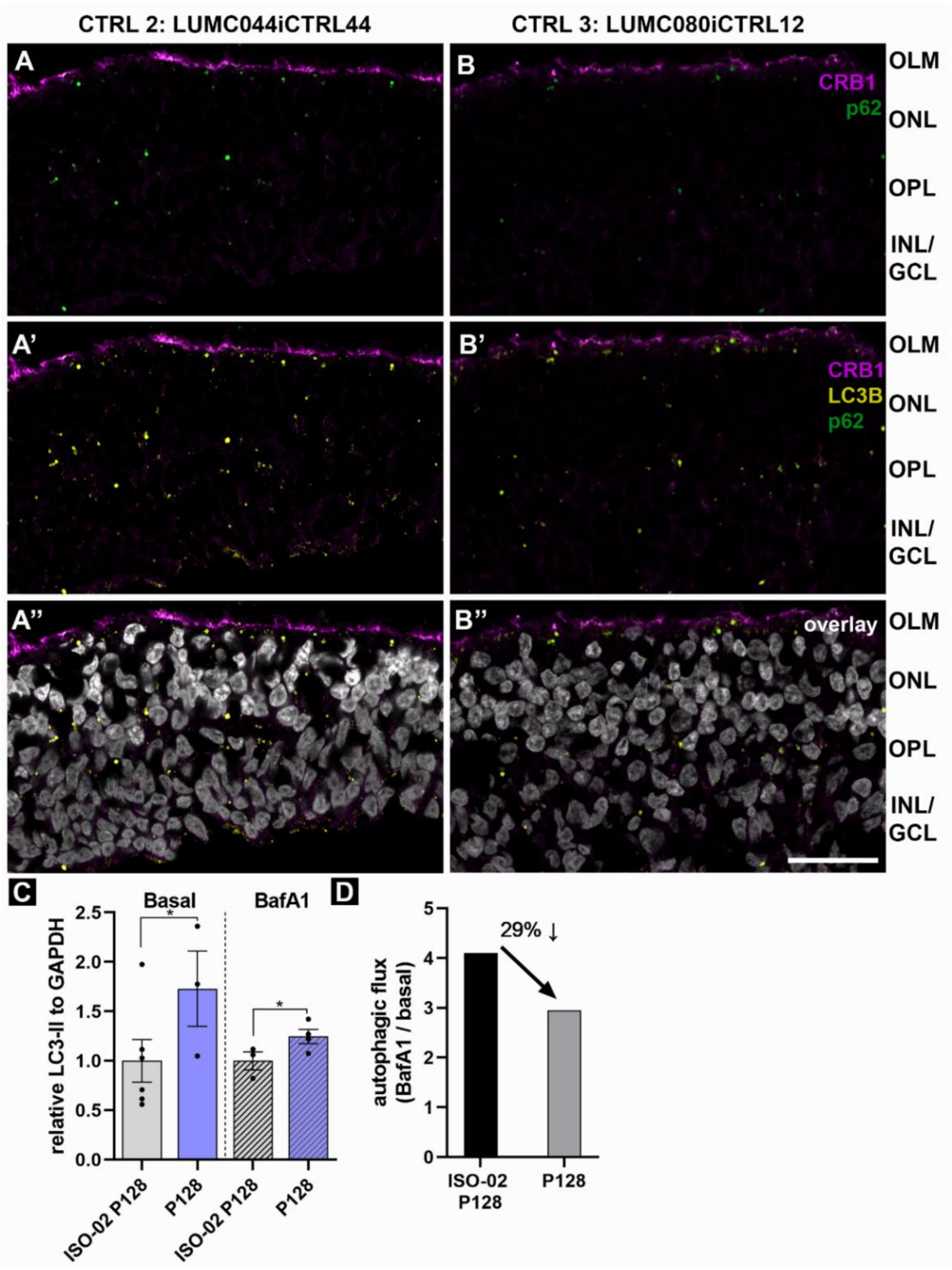

Figure S6. Increased number of degradative vesicles/compartments in *CRB1* patient retinal organoids. (A-B) Immunofluorescence triple staining CRB1 (magenta), p62 (green), and LC3B (yellow). (A-B) LC3B and p62 localized overall more in OLM layer, OPL layer and INL layer in control retinal organoids. Scale bar, 50 µm. (C-D) westernblot related to Figure 5 and Figure S6. (C) Analysis of the protein band intensities of the western blot shown in Figure 5I of individual lysed organoids (depicted as dots) stained for LC3B (LC3-I and LC3-II; 19/17 kD), recoverin for photoreceptors (26 kD), and GAPDH (housekeeping control, 37 kD) but organoids lacking recoverin expression were removed from the analysis. (D) Western blot protein band intensities of the BafA1 condition divided by the basal condition showing a decrease in the autophagic flux in *CRB1* patient retinal organoids. Each datapoint in the graphs J and K represents individual organoids, of which an average has been taken. The standard error of mean (SEM) is derived from these averages. Number of individual organoids per condition and differentiation round is n=3-6 organoids per line from two independent organoid batches. Statistical analysis: $p<0.05$ (*), and $p<0.001$ (***). Related to Figure 5.

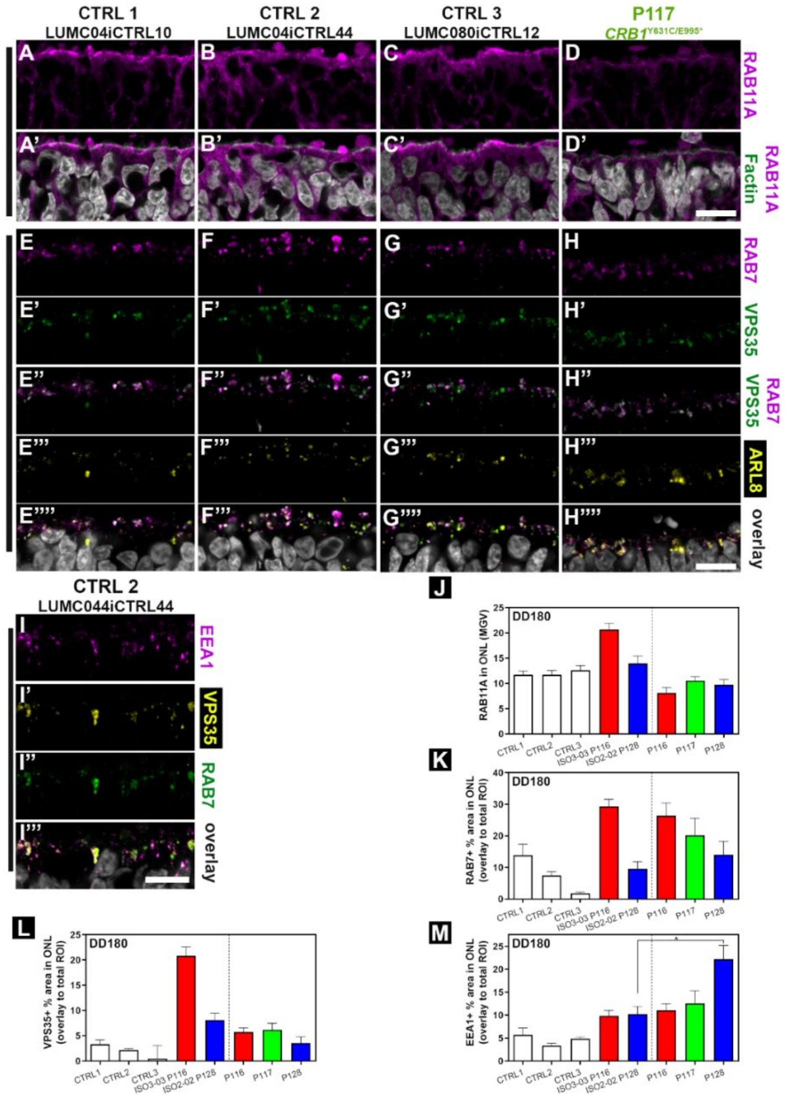

Figure S7. Dysregulation of the endolysosomal system in patient *CRB1* patient retinal organoids. (A+E) CTRL 1 (LUMC04CTRL10) retinal organoids, (B+F+I) CTRL 2 (LUMC044iCTRL44) retinal organoids, (C+G) CTRL3 (LUMC080iCTRL12) retinal organoids, and (D+H) patient 2 (LUMC0117iCRB01) retinal organoids. (A-D) Immunofluorescence labelling of RAB11A (magenta), and phalloidin (F-actin; green). (E-H) Immunofluorescence labeling of RAB7 (magenta), VPS35 (green), and ARL8A/B (yellow). (E-H) Immunofluorescence labeling of EEA1 (magenta), RAB7 (green), and ARL8A/B (yellow). (I) Immunofluorescence labeling of EEA1 (magenta), RAB7 (green), and VPS35 (yellow). Overlay shown in white (E-I). (J-M) Semi-quantification of fluorescence signal. (J) RAB11A mean gray value (MVG) in ONL. (K) Total RAB7 particle area / total ONL area in %. (L) RAB11A mean gray value (MVG) in ONL. (K) Total VPS35 particle area / total ONL area in %. (M) RAB11A mean gray value (MVG) in ONL. (K) Total EEA1 particle area / total ONL area in %. All retinal organoids at DD180 collected. Scale bar, 10 µm. Each datapoint in the graph represents individual organoids, of which an average has been taken of 3 representative images. The standard error of mean (SEM) is derived from these averages. Number of individual organoids per condition and differentiation round is n=6-14 organoids per line from two independent organoid batches; Statistical analysis: *$p < 0.05$. Related to Figure 6.

Table S1. Information on human iPSC lines. Related to Figure S3 and Figure 2-6.

**Line code**	**Description**	**Gender**
Control-derived and gene corrected (isogenic, iso) human iPSC line names		
LUMC0004iCTRL10	Control line 1 (CTRL 1)	male
LUMC0044iCTRL44	Control line 2 (CTRL 2)	female
LUMC0080iCTRL12	Control line 3 (CTRL 3)	male
iso01LUMC0116iCRB09	isogenic: Allele 1: c.3122T>C gene corrected to c.3120C>G. Allele 2: c.3122T>C. p.(Met1041Thr)	male
iso02LUMC0116iCRB09	isogenic: Allele 1: c.3122T>C gene corrected to c.3120C>G. Allele 2: c.3122T>C. p.(Met1041Thr)	male
iso03LUMC0116iCRB09	isogenic: Allele 1 and 2: Homozygous c.3122T>C gene corrected to c.3120C>G	male
iso01LUMC0128iCRB01	isogenic: Allele 1: c.2843G>A --> p.(Cys948Tyr). Allele 2: c.3122T>C gene corrected to c.3120C>G	male
iso02LUMC0128iCRB01	Isogenic: Allele 1: c.2843G>A. p.(Cys948Tyr). Allele 2: c.3122T>C gene corrected to c.3120C>G	male
iso03LUMC0128iCRB01	isogenic: Allele 1: c.2843G>A. p.(Cys948Tyr). Allele 2: c.3122T>C gene corrected to c.3120C>G	male
iso05LUMC0128iCRB01	isogenic: Allele 1: c.2843G>A. p.(Cys948Tyr). Allele 2: c.3122T>C gene corrected to c.3120C>G	male
Patient *CRB1* Retinitis pigmentosa-derived human iPSC lines		
LUMC0116iCRB09	Patient 1. Allele 1 and 2: homozygous c.3122T>C--> p.(Met1041Thr)	male
LUMC0117iCRB01	Patient 2. Allele 1: c.1892A>G (p.Tyr631Cys)). Allele 2: c.2911G>T (p.(Glu995*))	male
LUMC0128iCRB01	Patient 3. Allele 1: c.2843G>A --> p.(Cys948Tyr). Allele 2: c.3122T>C --> p.(Met1041Thr)	male

Table S2: Antibody list and dilution used for immunohistochemistry and western blots. Related to all figures.

Host; antibody	Dilution + procedure	Catalogue number
**Primary antibodies**		
Mouse anti-CRB1 (extracellular)	1/200 IF, 1/300 PLA	H00023418-A01
Rabbit anti-CRB1 (AK2; intracellular)	1/100 IF	Home-made
Phalloidin-TRITC (*aka* F-actin)	1/250 IF	r-415
Rabbit anti-OTX2	1/200 IF	13497-1-AP
Mouse anti-BRN3A	1/100 IF	sc-8429
Mouse anti-LC3B	1/100 IF + WB 1/300	0231-100/LC-3-5F10
Rabbit anti-FENS1 (*aka* WDFY1)	1/200 IF	AB125329
Mouse anti-EEA1	1/100 IF	BD-610457
Mouse anti-LAMP1 (aka CD107a)	1/100 IF	sc-20011
Rabbit anti-Cathepsin D (ab-2 clone)	1/200 IF	IM16-100UG
Mouse anti-ARL8A/B	1/100 IF	sc-398635
Mouse anti-NOTCH1 (extracellular)	1/300 IF + PLA	MA5-11961
Mouse anti-PIP2 (*aka* PtdIns(4,5)P_2)	1/200 IF	MA3-500
Rabbit anti-(pan-)cytokeratin	1/200 IF	AB9377
Rabbit anti-RCVRN	1/600 IF, 1/5000 WB	AB5585
Mouse anti-GAPDH	1/5000 WB	MAB374
Rabbit anti-RAB7	1/100 IF	CST9367S
Goat anti-VPS35 (retromer)	1/100 IF	Ab10099
Rabbit anti-VPS26 (retromer)	1/250 IF	Ab181352
Rabbit anti-RAB11a	1/100 IF	71-5300
Rat anti-CD44	1/100 IF	553132
Mouse anti-Oct3/4-BV421	1/25 FACS	565644
Mouse anti-NANOG-PE	1/5 FACS	560483
anti-SSEA4-FITC	1/25 FACS	130-098-371
Mouse anti-Nestin-Alexa488 CC	1/200 IF	CST; clone 10C2
Rabbit PAX6-Alexa647 c CC	1/200 IF	CST; clone D3A9V
Rabbit FOXA2-Alexa555 CC	1/500 IF	CST; clone D56D6
Rabbit GATA4-Alexa647 CC	1/200 IF	CST; clone D3A3M
Rabbit Vimentin-Alexa647 CC	1/400 IF	CST; clone D21H3
Rabbit CDX2-Alexa555 CC	1/500 IF	CST; clone D11D10
Rabbit Brachyury-Alexa488 CC	1/200 IF	CST; clone D2Z3J
**Secondary antibodies**		
Anti-rabbit-IgG-HRP	1/5000 WB	sc-2357
Anti-mouse-IgGκ BP-HRP	1/5000 WB	sc-516102
Anti-rabbit Alexa647	1/1000 IF	Ab150083
Anti-mouse Alexa647	1/1000 IF	Ab150119
Anti-mouse Alexa488	1/1000 IF	Ab150113
Anti-chicken Alexa555	1/1000 IF	Ab150169
Anti-rabbit Alexa555	1/1000 IF	Ab150086
Anti-rat cy3 (50% glycerol added)	1/500 IF	712-165-153
Anti-rabbit cy3 (50% glycerol added)	1/500 IF	111-165-045

*IF, immunofluorescence; WB, western blot; PLA, Proximity ligation assay; CC, custom made conjugated

Table S3: ISO-02-P116 top 10 off-target candidates. Related to figures 3-7.

**Gene Locus**	FW primer sequence (5' to 3')<br>RV primer sequence (5' to 3')	Target Sequence (5' to 3')	Off target mutation
Intron:RP11-132N15.3<br>**chr3:187689636-187689658:+**	FW 5'- TTGGGGCATACAAAGGAGGG - 3'<br>RV 5'- TTTCTGAATAGGCTGCCCGT - 3'	AGCCCGTGTT**CTGGAACAGAGAATCTGTCA**GGGGTGTG	None
Intron:RP1-91J24.3<br>**chr6:144636458-144636480:+**	FW 5' - ACATAAGCCAGCCTGCAGAA - 3'<br>RV 5' - GGCCCTGTCCAACTAGTGTC - 3'	GGATGGCTGG**CAGGGGCAGTAGGTCAGTCA**GGGCACTG	None
Intergenic: NARS-RP11-35G9.3<br>**chr18:55296831-55296853:-**	FW 5'- TCCTAAGGACGCCCTACTCT - 3'<br>RV 5' – TCCCAAACTCAGAGGCGTAAC - 3'	CTCAGCTTCC**CTGGACCAGTGGGTCAGTCA**GGGCGTGT	None
Intergenic: HNRNPA 1P57-AC010739.1<br>**chr2:41,415,579-41,415,601:-**	FW 5'- TGGAGCCAAACTCTCTGGGTT - 3'<br>RV 5'- AATTAGGTTCCTTCCTGGCCT - 3'	TGCCATATTC**CTAGGACACTGGATCAGTCA**AGGCCTAG	None
Intron: LINC01088<br>**chr4:79946400-79946422:+**	FW 5'- CACAGCAGCAACAGTCATGG - 3'<br>RV 5'- TTGTGGAGCACTCACCGTAG - 3'	GAGCTCTCAC**CTGGGAGAGAGGATCAGTCA**AGGTCTCT	None
Intergenic: SRSF1-RP11-159D12.10<br>**chr17:56132133-56132155:+**	FW 5' - AGACCCTGTTCTTCAGCCAC - 3'<br>RV 5'- GGAATCTGGTCAGTGCTCCC - 3'	TGTGGAAGTC**CTGGGGCACTGGATCAGTCA**AGGTCCTG	None
Intergenic:<br>RP1-124C6.1-AL513123.1<br>**chr6:113798294-113798316:-**	FW 5' - CTTCTGCCCACAAGGACCAT - 3'<br>RV 5'- AGTTGTAATGGCAGCCCACA 3'	ATGGACTCAT**CTAGGCCAGTGAGTCAGTCA**AGGCATTT	None
Intron: LHX6<br>**chr9:124983136-124983158:-**	FW 5'- TGGGGAGAGGTGTTACTGCT -3'<br>RV 5'- CCTTGTTCGCCTCACAGGAT -3'	GCATAGTAGG**CTGGGAAAGTGAGTCTGACT**TGGTCAAG	None
Intergenic: C10orf91-RP11-432J24.5<br>**chr10:134274249-134274271:+**	FW 5' - CTGCTCCTTGAGGGACAGTG - 3'<br>RV 5'- CACTGGCCTGGGGAAAGTAG - 3'	GTGGGATCCC**CTGGGGAAGAGGGTCTGTCT**GGGCTCGG	None
Intergenic: AL512655.1-MIR5007<br>**chr13:55536216-55536238:+**	FW 5'- GAGCAGGGCTTCCATCTTGA - 3'<br>RV 5'- TGGAAGCACTTAATGTAAACAGTG - 3'	TGTACTGCTG**TTGAGTCAGTGTGTCTGTCA**GGGAAGAA	None

**Table S4: ISO-03-P116 top 10 off-target candidates. Related to figures 3-7.**			
**Gene Locus**	FW primer sequence (5' to 3')<br>RV primer sequence (5' to 3')	Target Sequence (5' to 3')	Off target mutation
Intron:RP11-132N15.3<br>**chr3:187689636-187689658:+**	FW 5'- TTGGGGCATACAAAGGAGGG - 3'<br>RV 5'- TTTCTGAATAGGCTGCCCGT - 3'	AGCCCGTGTT**CTGGAACAGAGAATCTGTCA**GGGGTGTG	None
Intron:RP1-91J24.3<br>**chr6:144636458-144636480:+**	FW 5' - ACATAAGCCAGCCTGCAGAA - 3'<br>RV 5' - GGCCCTGTCCAACTAGTGTC - 3'	GGATGGCTGG**CAGGGGCAGTAGGTCAGTCA**GGGCACTG	None
Intergenic: NARS-RP11-35G9.3<br>**chr18:55296831-55296853:-**	FW 5'- TCCTAAGGACGCCCTACTCT - 3'<br>RV 5' – TCCCAAACTCAGAGGCGTAAC - 3'	CTCAGCTTCC**CTGGACCAGTGGGTCAGTCA**GGGCGTGT	None
Intergenic: HNRNPA 1P57-AC010739.1<br>**chr2:41,415,579-41,415,601:-**	FW 5'- TGGAGCCAAACTCTCTGGGTT - 3'<br>RV 5'- AATTAGGTTCCTTCCTGGCCT - 3'	TGCCATATTC**CTAGGACACTGGATCAGTCA**AGGCCTAG	None
Intron: LINC01088<br>**chr4:79946400-79946422:+**	FW 5'- CACAGCAGCAACAGTCATGG - 3'<br>RV 5'- TTGTGGAGCACTCACCGTAG - 3'	GAGCTCTCAC**CTGGGAGAGAGGATCAGTCA**AGGTCTCT	None
Intergenic: SRSF1-RP11-159D12.10<br>**chr17:56132133-56132155:+**	FW 5' - AGACCCTGTTCTTCAGCCAC - 3'<br>RV 5'- GGAATCTGGTCAGTGCTCCC - 3'	TGTGGAAGTC**CTGGGGCACTGGATCAGTCA**AGGTCCTG	None
Intergenic:<br>RP1-124C6.1-AL513123.1<br>**chr6:113798294-113798316:-**	FW 5' - CTTCTGCCCACAAGGACCAT - 3'<br>RV 5'- AGTTGTAATGGCAGCCCACA 3'	ATGGACTCAT**CTAGGCCAGTGAGTCAGTCA**AGGCATTT	None
Intron: LHX6<br>**chr9:124983136-124983158:-**	FW 5'- TGGGGAGAGGTGTTACTGCT -3'<br>RV 5'- CCTTGTTCGCCTCACAGGAT -3'	GCATAGTAGG**CTGGGAAAGTGAGTCTGACT**TGGTCAAG	None
Intergenic: C10orf91-RP11-432J24.5<br>**chr10:134274249-134274271:+**	FW 5' - CTGCTCCTTGAGGGACAGTG - 3'<br>RV 5'- CACTGGCCTGGGGAAAGTAG - 3'	GTGGGATCCC**CTGGGGAAGAGGGTCTGTCT**GGGCTCGG	None
Intergenic: AL512655.1-MIR5007<br>**chr13:55536216-55536238:+**	FW 5'- GAGCAGGGCTTCCATCTTGA - 3'<br>RV 5'- TGGAAGCACTTAATGTAAACAGTG - 3'	TGTACTGCTG**TTGAGTCAGTGTGTCTGTCA**GGGAAGAA	None

Table S5: ISO-02 P128 top 10 off-target candidates. Related to figures 3-7.

**Gene Locus**	FW primer sequence (5’ to 3’)<br>RV primer sequence (5’ to 3’)	Target Sequence (5’ to 3’)	Off target mutation
Intron:RP11-132N15.3<br>**chr3:187689636-187689658:+**	FW 5’- TTGGGGCATACAAAGGAGGG - 3’<br>RV 5’- TTTCTGAATAGGCTGCCCGT - 3’	AGCCCGTGTT**CTGGAACAGAGAATCTGTCA**GGGGTGTG	None
Intron:RP1-91J24.3<br>**chr6:144636458-144636480:+**	FW 5’ - ACATAAGCCAGCCTGCAGAA - 3’<br>RV 5’ - GGCCCTGTCCAACTAGTGTC - 3’	GGATGGCTGG**CAGGGGCAGTAGGTCAGTCA**GGGCACTG	None
Intergenic: NARS-RP11-35G9.3<br>**chr18:55296831-55296853:-**	FW 5’- TCCTAAGGACGCCCTACTCT - 3’<br>RV 5’ – TCCCAAACTCAGAGGCGTAAC - 3’	CTCAGCTTCC**CTGGACCAGTGGGTCAGTCA**GGGCGTGT	None
Intergenic: HNRNPA 1P57-AC010739.1<br>**chr2:41,415,579-41,415,601:-**	FW 5’- AGTCTGGAGCCAAACTCTCTG - 3’<br>RV 5’- TTCCTTCCTGGCCTTTGCTTA - 3’	TGCCATATTC**CTAGGACACTGGATCAGTCA**AGGCCTAG	None
Intron: LINC01088<br>**chr4:79946400-79946422:+**	FW 5’- CACAGCAGCAACAGTCATGG - 3’<br>RV 5’- TTGTGGAGCACTCACCGTAG - 3’	GAGCTCTCAC**CTGGGAGAGAGGATCAGTCA**AGGTCTCT	None
Intergenic: SRSF1-RP11-159D12.10<br>**chr17:56132133-56132155:+**	FW 5’ - AGACCCTGTTCTTCAGCCAC - 3’<br>RV 5’- GGAATCTGGTCAGTGCTCCC - 3’	TGTGGAAGTC**CTGGGGCACTGGATCAGTCA**AGGTCCTG	None
Intergenic:<br>RP1-124C6.1-AL513123.1<br>**chr6:113798294-113798316:-**	FW 5’ - CTTCTGCCCACAAGGACCAT - 3’<br>RV 5’- AGTTGTAATGGCAGCCCACA 3’	ATGGACTCAT**CTAGGCCAGTGAGTCAGTCA**AGGCATTT	None
Intron: LHX6<br>**chr9:124983136-124983158:-**	FW 5’- TGGGGAGAGGTGTTACTGCT -3’<br>RV 5’- CCTTGTTCGCCTCACAGGAT -3’	GCATAGTAGG**CTGGGAAAGTGAGTCTGACT**TGGTCAAG	None
Intergenic: C10orf91-RP11-432J24.5<br>**chr10:134274249-134274271:+**	FW 5’ - CTGCTCCTTGAGGGACAGTG - 3’<br>RV 5’- CACTGGCCTGGGGAAAGTAG - 3’	GTGGGATCCC**CTGGGGAAGAGGGTCTGTCT**GGGCTCGG	None
Intergenic: AL512655.1-MIR5007<br>**chr13:55536216-55536238:+**	FW 5’- GAGCAGGGCTTCCATCTTGA - 3’<br>RV 5’- TGGAAGCACTTAATGTAAACAGTG - 3’	TGTACTGCTG**TTGAGTCAGTGTGTCTGTCA**GGGAAGAA	None

<table>
<tr><td colspan="5">Table S6: Karyotyping of hiPS cell lines. Related to figures 2-7.</td></tr>
<tr><td>CTRL 1 LUMC04iCTRL10</td><td>male</td><td>P20</td><td>46,XY[20]</td><td>18 normal metaphases; 2 non-clonal aberration metaphases</td></tr>
<tr><td>CTRL 2 LUMC044iCTRL44</td><td>female</td><td>P18</td><td>46,XX,t(1;9)(p34;p13)[15]/46,XX[5]</td><td>5 normal metaphases; 15 metaphases with translocation of which 3 out of 15 in addition non-clonal aberration metaphases
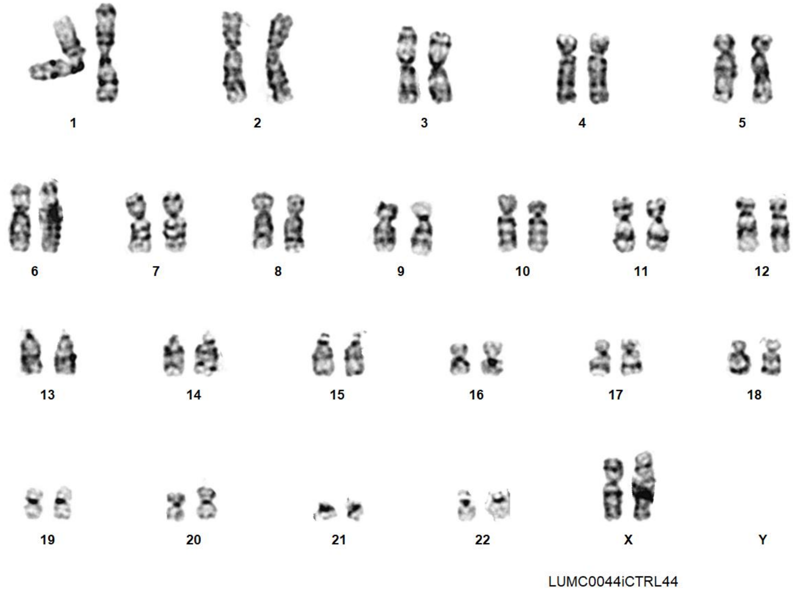
</td></tr>
<tr><td>CTRL 3 LUMC080iCTRL12</td><td>male</td><td>P25</td><td>46,XY[20]</td><td>20 normal metaphases</td></tr>
</table>

				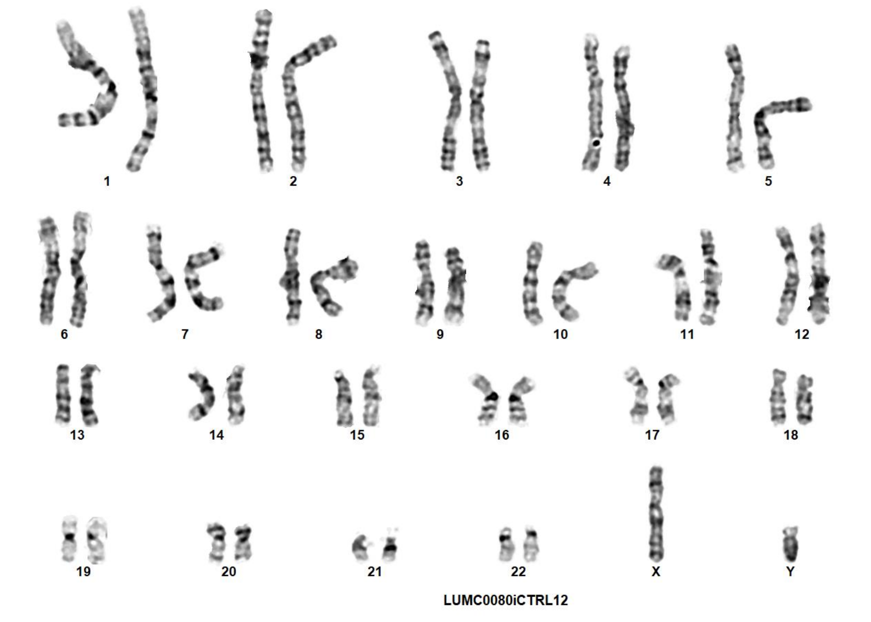
iso02LUMC0116iCRB09	male	P28	46,XY[20]	20 normal metaphases
iso03LUMC0116iCRB09	male	P27	46,XY[20]	20 normal metaphases
iso01LUMC0128iCRB01	male	P25	46,XY[20]	18 normal metaphases; 2 non-clonal aberration metaphases
iso02LUMC0128iCRB01	male	P25	46,XY[20]	19 normal metaphases; 1 non-clonal aberration metaphases
LUMC0116iCRB09	male	P13	46,XY[20]	20 normal metaphases
LUMC0117iCRB01	male	P9	46,XY[20]	19 normal metaphases; 1 non-clonal aberration metaphases
LUMC0128iCRB01	male	P10	46,XY[20]	19 normal metaphases; 1 non-clonal aberration metaphases

Supplemental EXPERIMENTAL PROCEDURES

Animals

Procedures concerning animals were performed with permission of the ethical committee of the Leiden University Medical Center and the animal experimentation committee (DEC) of the Royal Netherlands Academy of Arts and Sciences (KNAW) under permit number NIN 12.105. All mice used were maintained on a 99.9% C57BL/6JOlaHsd genetic background with a 12 h day-night cycle and supplied with food and water *ad libitum*. $Crb1^{KO}$ ($Crb1^{-/-}$) mice (van de Pavert et al., 2004) were crossed with a retinal $Crb2^{\Delta RPC}$ ($Crb2^{F/F}$Chx10$Cre^{Tg/+}$ clone P1E9) mouse strain (Alves et al., 2013b; Rowan and Cepko, 2004) to ablate both *Crb1* and *Crb2* during retinal development from retinal progenitor cells. *Cre* starts to be expressed around E9.5 in the retina (Rowan and Cepko, 2004). The previously made Chx10*Cre* mice were generated by knocking in *GFP*-*Cre*-IRES-*AP* cDNA in exon-1 of the *Chx10* gene on a Bacterial Artificial Chromosomes (BAC; -55 kb *Chx10* of ATG to +22 kb off of the polyadenylation sequence) (Rowan and Cepko, 2004). Interestingly, the last exon of the *Abcd4* gene is only ~7000 bp from the last exon of the *Chx10* gene thus was on the BAC. Others have shown that large BACS containing several genes can lead to overexpression of flanking genes (Kolisnyk et al., 2013; Ting and Feng, 2014). We believe the integration of the cDNA in close proximity to the *Abcd4* gene and being both on the BAC may have led to the overexpression of the *Abcd4* gene in $Crb1^{KO}Crb2^{\Delta RPC}$ mice. $Crb1^{KO}Crb2^{\Delta RPC}$ mice were compared to littermate $Crb1^{KO}Crb2^{F/F}$ mice not expressing *Cre*. C57BL/6JOlaHsd mice have a 365 kb deletion ablating the *Mmrn1* and *Snca* and express low levels of *Abcd4* (Gajovic et al., 2006; Specht and Schoepfer, 2004). The C57BL/6J substrain (from the Jackson Laboratory) carries a 5-exon-spanning deletion in the *Nnt* gene but C57BL/6JOlaHsd have a wildtype *Nnt* gene (Huang et al., 2006). Analysis of the $Crb1^{KO}Crb2^{\Delta RPC}$ and $Crb1^{KO}Crb2^{F/F}$ mice was carried out on males and females. Chromosomal DNA isolation and genotyping were performed as previously described (Alves et al., 2013a; van de Pavert *et al.*, 2004).

Morphology and retinal thickness measurements on plastic mouse eyes

Eyes were collected of double-knockout and control littermate mice. After enucleation, the eyes were fixed in 4% paraformaldehyde in PBS (25 min) and dehydrated in an ethanol series (30 min each step; 30 % EtOH , 50 % EtOH, 70 % EtOH, 2x 90 % EtOH, 2x 100% EtOH). Then, the eyes were hardened in Technovit, sectioned (3 µm), stained (0.5% Toluidine blue), and mounted (Entellan) as previously described [(Alves and Wijnholds, 2018)]. Consecutive

brightfield images were taken. We generated spidergrams of the retinal thickness (ILM-to-OLM) for P14, 1 M and 3M-old-mice by measuring at 0.25, 0.5, 0.75, 1.0, 1.25, 1.5, 1.75, 2.0, 2.25, 2.5, and 2.75 mm distance to the optic nerve head (ONH) as previously described (Alves and Wijnholds, 2018).

Electroretinography

Electroretinography's (ERGs) were done in dim light on an Espion E2 (Diagnosys, LLC, MA). 1-month-old (1M) mice were dark-adapted (>12 hours). The mice were anesthetized (100 mg/kg ketamine, 10 mg/kg xylazine, intraperitoneal injection) and the pupils were dilated (atropine drops, 5 mg/mL). Scotopic flash series: -4, -3 , -2, -1, 0, 1, 1.5, 1.9 log cd s/m² light intensity. Photopic flash series at 30 cd/m² background light: -2, -1, 0, 1, 1.5, 1.9 log cd s/m² light intensity. We first recorded the scotopic ERG, followed by a 10 min light exposure (30 cd s/m² light intensity) and then the photopic ERG.

RNA sequencing

Sequencing was performed using Life Technologies SOLiD5500 with single-end, 50 bp reads. Two separate runs were performed. Run1 contained n=30 mice; n=5 *Crb1*^{KO}*Crb2*$^{F/F}$ (*Crb1*^{KO}) and n=5 *Crb1*^{KO}*Crb2*$^{\Delta RPC}$ (Tg) per development timepoint of the mouse embryo at E15.5, E17.5 and P1. Run2 contained n=9 mice; n=4 *Crb1*^{KO}*Crb2*$^{\Delta RPC}$ mice (C57BL/6JOlaHsd) and n=5 healthy mice (C57BL/6JRccHsd = wildtype, WT) at E15.5. Reads were aligned against mm10, using the 'whole.transcriptome.frag' workflow with bamgen.mqv.threshold set to 20 (Lifescope v2.5). Counts were obtained using the GTF as supplied by Lifescope (transformation of refGene.txt downloaded from UCSC at 25-06-2014) within this workflow.

Differential expression analysis

The analysis was performed as previously (Pellissier et al., 2013). The two runs were analysed separately. Statistical analyses were performed using the edgeR (Robinson and Oshlack, 2010) and limma/voom (Ritchie et al., 2015) R/Bioconductor packages. One of the *Crb1*^{KO}*Crb2*$^{\Delta RPCC}$ E15.5 samples in the first run was identified as an outlier and removed from the analysis. Genes with more than one count in five or more samples were retained. Count data were transformed to log2-counts per million (logCPM), normalized by applying the trimmed mean of M-values method (Robinson and Oshlack, 2010) and precision weighted using voom (Law et al., 2014). Differential expression was assessed using an empirical Bayes moderated t-test within limma's linear model framework

including the precision weights estimated by voom. Resulting p-values were corrected for multiple testing using the Benjamini-Hochberg false discovery rate. Additional gene annotation was retrieved from Ensembl (release 90) using the biomaRt R/Bioconductor package. Analyses were performed using R v3.4.1 and Bioconductor v3.5.

Real-time quantitative PCR

RNA was isolated from 5 controls (*Crb1*^{KO}) and *Crb1*^{KO}*Crb2*$^{\Delta RPC}$ retinas using TRIZOL reagent (Gibco life technologies), according to the manufacturer manual, and after the final precipitation dissolved in RNase-free water. After genomic DNA degradation with RNase- free DNase I (New England Biolabs), 1 µg of total RNA was reverse transcribed into first-strand cDNA with Superscript III Plus RNase H-Reverse Transcriptase (Invitrogen) and 50 ng random hexamer primers, during 50 min at 50°C in a total volume of 20 µl. To the resulting cDNA sample, 14 µL of 10 mM Tris, 1 mM EDTA was added. From all samples, a 1:20 dilution was made and used for qPCR analysis. For this analysis, primer pairs were designed with a melting temperature of 60–62°C, giving rise to an amplicon of 80–110 bp. Real-time qPCR was based on the real-time monitoring of SYBR Green I dye fluorescence on a ABI Prism 7300 Sequence Detection System (Applied Biosystems, Nieuwekerk a/d IJssel, The Netherlands). The PCR conditions were as follows: 12.5 µL SYBR Green PCR 26mastermix (Applied Biosystems), 20 pmol of primers, and 2 µl of the diluted cDNA (ca 3 ng total RNA input). An initial step of 50°C for 2 min was used for AmpErase incubation followed by 15 min at 95°C to inactivate AmpErase and to activate the AmpliTaq. Cycling conditions were as follows: melting step at 95°C for 1 min, annealing at 58°C for 1 min and elongation at 72°C, for 40 cycles. At the end of the PCR run, a dissociation curve was determined by ramping the temperature of the sample from 60 to 95°C while continuously collecting fluorescence data. Non template controls were included for each primer pair to check for any significant levels of contaminants. Values were normalized by the geometric mean of the 3 reference genes hypoxanthine-guanine phosphoribosyltransferase, elongation factor 1-alpha and ribosomal protein S27a.

Cell culture

We previously described the three male *CRB1* RP-patient human induced pluripotent stem cell (hiPSC) lines (Patient line 1-3: LUMC0116iCRB09, LUMC0117iCRB01, LUMC0128iCRB01) and three control lines (CTRL 1-3: LUMC0004iCTRL10 (hPSC^{reg} name: LUMCi029-B), LUMC0044iCTRL44,

LUMC0080iCTRL12; see also Table S1; (Quinn et al., 2019). Human iPSCs were maintained on mTeSR1 or mTeSR Plus on matrigel-coated plates and stored in mFreSR (STEMCELL Techologies) or CryoStor CS10 in liquid nitrogen for long-term storage. The pluripotency status and functional pluripotency was performed for at least 3 clones per mutated line or isogenic controls (**Figure S3 C**). hiPSC clones were characterized on pluripotency by triple-staining dissociated single cells with OCT3/4, NANOG, and SSEA4 ($1x10^5$ cells/sample; 1 hr at RT) and analyzed by FACS (Flow cytometer, Miltenyi-Vyb). hiPSCs were also differentiated into the three germlayers (STEMdiff Trilineage differentiation kit, Stem Cell Technologies) and stained on coverslips with the conjugated antibodies Nestin (ectoderm), PAX6 (ectoderm), FOXA2 (endoderm), GATA4 (endoderm), vimentin (mesoderm), CDX2 (mesoderm), Brachyury (mesoderm; blocking solution: 4% NSS/PBS with 0.1% Triton X-100; cells incubated with primary antibodies in 4%NSS/PBS for 1 hr at RT). **Figure S3 F-G** and see **Table S2** antibody concentrations).

CRISPR/Cas9-based gene-repair of the hiPSC lines LUMC0116iCRB09 and LUMC0128iCRB01

The *CRB1* variant c.3122T>C (exon 9) was repaired by CRISPR/Cas9 ribonucleoprotein (RNP) mediated homologous recombination. Sequences of the sgRNA and the repair template (ssODN) are provided below. $1x10^5$ single cells were transfected using electroporation (Neon System). The CRISPR/Cas9 RNP complex (crRNA:tracrRNA duplex (IDT); 10 pmol of crRNA/tracrRNA pre-annealed (IDT)) and the Cas9 V3 (1 µg; IDT) was transfected. Additionally, the homology directed (HRD) repair template (ssODN; 40 pmol) was added to correct the mutation. For screening of targeted clones, a restriction site is introduced via a silent mutation to avoid re-cutting after HDR (Figure S3 B). $3.6x10^5$ cells in 27 µL were mixed with 6 µL ssODN (13.3 µM) and 3 µL Cas9/RNP complex (3.6 µg Cas9, 36 pmol pre-annealed crRNA:tracrRNA). Then, three times 10 µL ($1.0x10^5$ cells per tube. 30 µL and $3.0x10^5$ cells in total per line) were electroporated (Neon System; Thermo Fisher Scientific; 1100V, 20 ms, 2 pulses), then plated on mTeSR-E8 + CloneR supplement (STEMCELL Technologies) into 12-well plates coated with Synthemax® II-SC substrate (Corning) and placed into an incubator (37°C, 5% CO_2, 20% O_2). Human iPSCs were seeded as single cells 5-7 days post-electroporation (1000 cells / 10-cm plate). Colonies were picked and gDNA isolated (QuickExtract) 8 days after seeding. Genomic DNA covering the variant region was amplified and targeting was analyzed using Taq1 (NEB; R0149) restriction of the PCR fragment. No off-target integrations were observed (Tables S3-5). All but control clone LUMC0044iCTRL44 showed a normal karyotype (Table S6). Successful targeting was confirmed by Sanger sequencing (*CRB1* gene exon 9. **Figure S3 D-E**).

Cas9 nuclease V3 (IDT #1081058); tracrRNA (IDT #1072532); crRNA (IDT, custom-made)

ssODN: ACAAGTTTGCAGTCAGTGAATGATGGCACATGGCACGAAGTGACCCTTTCGATGACAGACCCACTGTCCCAGACCTCCAGGTGGCAAATGGAAGTGGACA

Sequence of crRNA(s) used: CTGGGACAGTGGGTCTGTCG

Sanger sequencing primers:

Fwd: ACCAGAGAACTCACCAATATCAC

Rev: CAATAGCTCTGTCTCCCACATAA

Retinal organoid differentiation

The differentiation of the hiPSCs started at differentiation day (DD) 0 with floating single-cell hiPSCs that were self-aggregated to embryoid bodies in 10 µM (±)blebbistatin in mTeSR1 or mTeSR Plus medium (STEMCELL Technologies) on 1% agarose-coated (non-adhesive) plates overnight. The media was gradually changed from mTeSR to the Neural Induction Medium (NIM-1) over three days (mTeSR1/NIM-1: day 0, 1:0, day 1, 3:1; day 2, 1:1, day 3, 0:1). NIM-1 consists of DMEM/F12 supplemented with 1xN2 supplement, 1x Minimum Essential Media Non-Essential Amino Acids (MEM NEAAs) and 2 µg/mL Heparin (Sigma. See also (Quinn *et al.*, 2019)). The medium was changed every day until DD7. The embryoid bodies were plated at an approximate density of 20 aggregates per cm^2 onto Matrigel-coated wells on DD7 and lifted on DD22/28. NIM-1 was replaced every other day from DD7 to DD16, from DD16 medium was replaced each day with Neural induction medium 2 (NIM-2). NIM-2: 1x B27 or NeuroCult SM1 without Vitamin A, 1x NEAA and 1x antibiotic-antimycotic in DMEM/F12(3:1). Subsequently, the lifted organoids were dislodged and kept in floating culture with NIM-2 on agarose-coated plates. At DD33 forebrain organoids were removed with a truncated P200 pipet tip. Medium was replaced three times a week from DD41 onwards. The medium was changed to Retinal Lamination Medium 1 (RLM-1) at DD42. RLM-1: 10% FBS, 1x B27 without Vitamin A (Gibco) or NeuroCult™ SM1 without Vitamin A (Stem Cell Technologies), 1x NEAA, 1x antibiotic-antimycotic, and 100 µM taurine in DMEM/F12(3:1). The RLM-1 was supplemented with 1 µM retinoic acid from DD49 to DD98. The long-term culture medium from DD98 onwards was Retinal Lamination Medium-2 (RLM-2) with low amounts of growth factors (N2 supplement instead of B27). RLM-2: 10% FBS, 1x N2, 1x NEAA, 1x antibiotic-antimycotic, and 100 µM taurine in DMEM/F12(3:1) 0.5 µM retinoic acid.

Fixation, sectioning and immunohistochemical staining

Retinal organoids were collected at the time points of differentiation day(DD)90, 120, 150, and 180. Prior to fixation, the organoids were washed shortly in PBS and then incubated for 30 minutes in 4% paraformaldehyde in PBS. Subsequently, the organoids were dehydrated in 15% sucrose in PBS (30 min) and 30% sucrose in PBS (30 min). After that, the retinal organoids were orientated and embedded in OCT Tissue-Tek cryo-embedding media. Finally, they were frozen on dry-ice and stored at -20°C. The retinal organoids were sectioned at 7 µm using a Leica CM1900 cryostat (Leica Microsystems) and after 1 hour of airdrying stored at -20°C. The slides were rehydrated in PBS, blocked (1 hour. 0.1% BSA 0.04% Triton-X in PBS), stained with the primary antibody (in 0.1% BSA 0.04% Triton-X in PBS) overnight at 4°C or 3 hours at room temperature, washed three times in PBS (3x10 min), stained with the secondary antibody (1 hour), washed three times in PBS (3x10 min), and mounted with Vectashield® Vibrance® HardSet antifade mounting media containing DAPI (Vector Laboratories). For antibodies and dilutions see Table S2. A Leica TCS SP8 confocal microscope was used for image acquisition. Image analysis was done in Leica Application Suite X (Leica, LAS X), Fiji ImageJ, and Adobe Photoshop CC2018.

Fluorescence quantification in ROIs

All organoids (7-µm sectioned) imaged for fluorescence semi-quantification were stained (see Table S2) with the same antibody mix at the same time, imaged in one confocal microscopy session, and included a negative control (no primary antibody added). At least six different organoids (generally 10-12) per line on generally three glass slides were analyzed per immunofluorescence staining. Several experiments were stained twice and analyzed twice showing overall high robustness of outcome parameters. The semi-quantification of CRB1 protein expression was done in triplicates with different secondary antibodies and incubation times (overnight at +4°C or 3 hours at RT) showing a strong reduction in patient organoids in all conditions. The analysis was done blinded. A 8-bit gray-scale raw image file was loaded into ImageJ. We manually defined three ROIs in the retina: ROI1, OLM (±2.5 µm of OLM); ROI2, outer nuclear layer (dense bright DAPI+ nuclei layer or OTX2+ nuclei layer below the ROI 1), ROI3, OPL/INL (nuclei layer below the ROI2 of spanning the retina maximally 50 µm below ROI2). First, the mean gray value (MGV) per ROI was analysed. Then, we measured on each ROI the average particle size (area), the number of particles (counts), and the average intensity per spot (value between 0-255) using the ImageJ function "Analyze Particles". We normalized the values to: counts per 100 $µm^2$ and the area of all spot sizes per ROI area (%).

Isolation of protein lysates and immunoblotting

Retinal organoids were collected at DD180. The RPE (dark parts) were dissected off with surgical scissors. Single retinas were washed in cold 1xPBS and then lysed in cold RIPA buffer (#R0278, Sigma) including the protease inhibitor cocktail (cOmplete, #11836153001, Roche). The single retinas were mechanical disrupted by pipetting and incubated on ice for 2 hours. Then, the retinas were vortexed and then sonicated in a pre-cooled +4^{0}C water bath (Diagenode Biorupter Pico; Program: 15 sec ON, 30 sec OFF. 4 cycles; vortexed between 1st and 2nd cycle). The samples were then spun down (16000xg, 10 min, +4°C) and the supernatant was collected in a new pre-cooled tube. The protein concentration was measured by the Bradford assay (BCA Protein Assay Kit; ThermoFisher, #23227). The protein was then diluted to 1x Laemmli buffer with DTT (Bio-Rad, #1610737. Buffer: 0.12 M Tris-HCl, pH 6.8, 4% SDS, 20% glycerol, 10 mM DTT), boiled for 5 min (+95°C), and stored at -80°C. The protein lysates (20 µg per organoid per lane) were separated on a 4-20% SDS gel (Bio-Rad, #4561094) and transferred on PVDF (Bio-Rad Turbo transfer system. Protocol: mixed-molecular weight). The blots were blocked in 5% non-fatty milk in 1xPBS 0.1% Tween-20 (PBST; RT, 1 hour), incubated with the primary antibody (see Table S2 for concentrations; 5% milk in PBST), washed, incubated with the HPR-conjugated-antibody (RT, 1 hour 5% milk in PBST), and washed again. The signal was generated in ECL substrate (Bio-Rad, #1705061) and the chemiluminescence was imaged at three different exposure times (ChemiDoc MP Imaging System, Bio-Rad). The band intensity was measured (ImageJ, Gel analyzer) and averaged from three exposure times. Quantification was relative to GAPDH.

Transmission electron microscopy

Samples were washed in 1xPBS and then fixed in 1.5% glutaraldehyde in 0.1M cacodylate buffer at room temperature for 1 hour, stored in 0.5% PFA in 0.1M PHEM storage buffer until further processing. Samples were rinsed three times in 0.1 M cacodylate buffer and post-fixed in 1%OsO_4/1.5% potassium ferricyanide in 0.1M cacodylate buffer (1 hour on ice). Samples were rinsed again in 0.1M cacodylate buffer three times, and dehydrated in a series of ethanol, followed by a series of propylene oxide with EPON (LX112, Ladd research industries) and finally a step in 100% EPON. Organoids were put in a mould, those were filled up with EPON and polymerized at 70°C over two days. Ultrathin sections (90 nm) were created on a Reichert Ultracut S (Leica Microsystems) and after staining with uranylacetate and lead citrate, examined on an electron microscope (Microscope: FEI Tecnai T12 Twin Fei Company, Eindhoven, The

Netherlands; Camera: OneView, Gatan) operating at 120 kV. Overlapping images were collected and stitched together into separate images as previously described (Faas et al., 2012).

Conjugation of NOTCH1 and CRB1 antibody to plus and minus oligonucleotide probes

We used the Duolink probemaker set (Sigma-Aldrich) to conjugate two same-species antibodies (mouse anti-NOTCH1 and mouse anti-CRB1 extracellular domain antibodies) to the plus or minus oligonucleotide probes as described in the protocol. In short: 20 µL of the primary antibody was mixed with 2 µL of the conjugation buffer. Subsequently, the antibodies were added to the Duolink In Situ Probemaker plus and minus vials for overnight incubation at room temperature. The next day, the stop reagent was added (incubation: 30 minutes, at RT), the storage solution was added and stored at +4°C.

Proximity ligation assay

For the proximity ligation assay the Duolink In Situ Detection Reagents kit green (Sigma-Aldrich) was used. Slides with sliced organoids were washed, and the tissue slices were circled with a hydrophobic pen. Slides were blocked with one drop of blocking solution from the Duolink PLA probe set per tissue slice and incubated for 1 hour at 37 °C in a humidity chamber. Blocking buffer was tapped of and the conjugated antibodies diluted in PLA probe diluent from the PLA probe kit were added for an overnight incubation at 4°C. After that the slides were washed twice in PLA wash buffer A (Sigma-Aldrich) for five minutes. For the ligation step the slides were incubated for 30 minutes at 37°C with ligase enzyme diluted (1:40) in 1x ligation buffer and were washed twice in wash buffer A again. Finally, the polymerase enzyme was diluted (1:80) in 1x amplification buffer and added to the slides and the slides were incubated in a humidity chamber (37 °C, 100 minutes). To one of the slides diluted amplification buffer was added without the polymerase, serving as a negative control. Subsequently, the slides were washed twice for 10 minutes in PLA wash buffer B and after that once for 1 minute in 0.01x wash buffer B. Wash buffer was tapped off and slides were mounted by adding 5 µL of Duolink In Situ Mounting Medium with DAPI with a 20µl pipet with truncated tip. Images were obtained using a Leica TCS SP8 confocal microscope.

REFERENCES

Alves, C.H., Bossers, K., Vos, R.M., Essing, A.H., Swagemakers, S., van der Spek, P.J., Verhaagen, J., and Wijnholds, J. (2013a). Microarray and morphological analysis of early postnatal CRB2 mutant retinas on a pure C57BL/6J genetic background. PLoS One *8*, e82532. 10.1371/journal.pone.0082532.

Alves, C.H., Sanz, A.S., Park, B., Pellissier, L.P., Tanimoto, N., Beck, S.C., Huber, G., Murtaza, M., Richard, F., Sridevi Gurubaran, I., et al. (2013b). Loss of CRB2 in the mouse retina mimics human retinitis pigmentosa due to mutations in the CRB1 gene. Hum Mol Genet *22*, 35-50. 10.1093/hmg/dds398.

Alves, C.H., and Wijnholds, J. (2018). AAV Gene Augmentation Therapy for CRB1-Associated Retinitis Pigmentosa. Methods Mol Biol *1715*, 135-151. 10.1007/978-1-4939-7522-8_10.

Faas, F.G., Avramut, M.C., van den Berg, B.M., Mommaas, A.M., Koster, A.J., and Ravelli, R.B. (2012). Virtual nanoscopy: generation of ultra-large high resolution electron microscopy maps. J Cell Biol *198*, 457-469. 10.1083/jcb.201201140.

Gajovic, S., Mitrecic, D., Augustincic, L., Iaconcig, A., and Muro, A.F. (2006). Unexpected rescue of alpha-synuclein and multimerin1 deletion in C57BL/6JOlaHsd mice by beta-adducin knockout. Transgenic Res *15*, 255-259. 10.1007/s11248-006-0003-6.

Huang, T.T., Naeemuddin, M., Elchuri, S., Yamaguchi, M., Kozy, H.M., Carlson, E.J., and Epstein, C.J. (2006). Genetic modifiers of the phenotype of mice deficient in mitochondrial superoxide dismutase. Hum Mol Genet *15*, 1187-1194. 10.1093/hmg/ddl034.

Kolisnyk, B., Guzman, M.S., Raulic, S., Fan, J., Magalhaes, A.C., Feng, G., Gros, R., Prado, V.F., and Prado, M.A. (2013). ChAT-ChR2-EYFP mice have enhanced motor endurance but show deficits in attention and several additional cognitive domains. J Neurosci *33*, 10427-10438. 10.1523/JNEUROSCI.0395-13.2013.

Law, C.W., Chen, Y., Shi, W., and Smyth, G.K. (2014). voom: Precision weights unlock linear model analysis tools for RNA-seq read counts. Genome Biol *15*, R29. 10.1186/gb-2014-15-2-r29.

Pellissier, L.P., Alves, C.H., Quinn, P.M., Vos, R.M., Tanimoto, N., Lundvig, D.M., Dudok, J.J., Hooibrink, B., Richard, F., Beck, S.C., et al. (2013). Targeted ablation of CRB1 and CRB2 in retinal progenitor cells mimics Leber congenital amaurosis. PLoS Genet *9*, e1003976. 10.1371/journal.pgen.1003976.

Quinn, P.M., Buck, T.M., Mulder, A.A., Ohonin, C., Alves, C.H., Vos, R.M., Bialecka, M., van Herwaarden, T., van Dijk, E.H.C., Talib, M., et al. (2019). Human iPSC-Derived Retinas Recapitulate the Fetal CRB1 CRB2 Complex Formation and Demonstrate that Photoreceptors and Muller Glia Are Targets of AAV5. Stem Cell Reports *12*, 906-919. 10.1016/j.stemcr.2019.03.002.

Ritchie, M.E., Phipson, B., Wu, D., Hu, Y., Law, C.W., Shi, W., and Smyth, G.K. (2015). limma powers differential expression analyses for RNA-sequencing and microarray studies. Nucleic Acids Res *43*, e47. 10.1093/nar/gkv007.

Robinson, M.D., and Oshlack, A. (2010). A scaling normalization method for differential expression analysis of RNA-seq data. Genome Biol *11*, R25. 10.1186/gb-2010-11-3-r25.

Rowan, S., and Cepko, C.L. (2004). Genetic analysis of the homeodomain transcription factor Chx10 in the retina using a novel multifunctional BAC transgenic mouse reporter. Dev Biol *271*, 388-402. 10.1016/j.ydbio.2004.03.039.

Specht, C.G., and Schoepfer, R. (2004). Deletion of multimerin-1 in alpha-synuclein-deficient mice. Genomics *83*, 1176-1178. 10.1016/j.ygeno.2003.12.014.

Ting, J.T., and Feng, G. (2014). Recombineering strategies for developing next generation BAC transgenic tools for optogenetics and beyond. Front Behav Neurosci *8*, 111. 10.3389/fnbeh.2014.00111.

van de Pavert, S.A., Kantardzhieva, A., Malysheva, A., Meuleman, J., Versteeg, I., Levelt, C., Klooster, J., Geiger, S., Seeliger, M.W., Rashbass, P., et al. (2004). Crumbs homologue 1 is required for maintenance of photoreceptor cell polarization and adhesion during light exposure. J Cell Sci *117*, 4169-4177. 10.1242/jcs.01301.
