# Supplementary material for: CRB1 is required for recycling by RAB11A+ vesicles in human retinal organoids
Source: Stem Cell Reports. 2023 Aug 3;18(9):1793–810. doi: 10.1016/j.stemcr.2023.07.001 (PMC10545476; doi:10.1016/j.stemcr.2023.07.001)
Supplement: Document S2. Article plus supplemental information [file mmc2.pdf]

Stem Cell Reports
Article
OPEN ACCESS

CRB1 is required for recycling by RAB11A+ vesicles in human retinal organoids

Thilo M. Buck,[1] Peter M.J. Quinn,[1,9] Lucie P. Pellissier,[2,10] Aat A. Mulder,[3] Aldo Jongejan,[4] Xuefei Lu,[1] Nanda Boon,[1] Daniëlle Koot,[1,11] Hind Almushattat,[1] Christiaan H. Arendzen,[5] Rogier M. Vos,[2] Edward J. Bradley,[7] Christian Freund,[5] Harald M.M. Mikkers,[3,5] Camiel J.F. Boon,[1,6] Perry D. Moerland,[4] Frank Baas,[7,8] Abraham J. Koster,[3] Jacques Neefjes,[3] Ilana Berlin,[3] Carolina R. Jost,[3] and Jan Wijnholds[1,2,*]

[1]Department of Ophthalmology, Leiden University Medical Center (LUMC), Leiden 2333 ZA, the Netherlands
[2]Netherlands Institute for Neuroscience, Royal Netherlands Academy of Arts and Sciences (KNAW), Amsterdam 1105 BA, the Netherlands
[3]Department of Cell & Chemical Biology, Leiden University Medical Center (LUMC), Leiden 2300 RC, the Netherlands
[4]Bioinformatics Laboratory, Epidemiology & Data Science, Amsterdam University Medical Centers, Amsterdam 1105 AZ, the Netherlands
[5]Leiden University Medical Center hiPSC Hotel, Leiden 2333 ZA, the Netherlands
[6]Department of Ophthalmology, Amsterdam University Medical Centers, Academic Medical Center, University of Amsterdam, Amsterdam 1000 AE, the Netherlands
[7]Department of Genome Analysis, Amsterdam University Medical Centers, Amsterdam 1105 AZ, the Netherlands
[8]Department of Clinical Genetics/LDGA, Leiden University Medical Center, Leiden 2333 ZA, the Netherlands
[9]Present address: Edward S. Harkness Eye Institute, Department of Ophthalmology, Columbia University Irving Medical Center/New York-Presbyterian Hospital, New York, NY, USA
[10]Present address: Team Biology of GPCR Signaling Systems (BIOS), CNRS, IFCE, INRAE, Université de Tours, PRC, 37380, Nouzilly, France
[11]Present address: Netherlands Cancer Institute, 1066 CX Amsterdam, the Netherlands
*Correspondence: j.wijnholds@lumc.nl
https://doi.org/10.1016/j.stemcr.2023.07.001

SUMMARY

CRB1 gene mutations can cause early- or late-onset retinitis pigmentosa, Leber congenital amaurosis, or maculopathy. Recapitulating human *CRB1* phenotypes in animal models has proven challenging, necessitating the development of alternatives. We generated human induced pluripotent stem cell (iPSC)-derived retinal organoids of patients with retinitis pigmentosa caused by biallelic *CRB1* mutations and evaluated them against autologous gene-corrected hiPSCs and hiPSCs from healthy individuals. Patient organoids show decreased levels of CRB1 and NOTCH1 expression at the retinal outer limiting membrane. Proximity ligation assays show that human CRB1 and NOTCH1 can interact via their extracellular domains. *CRB1* patient organoids feature increased levels of WDFY1+ vesicles, fewer RAB11A+ recycling endosomes, decreased VPS35 retromer complex components, and more degradative endolysosomal compartments relative to isogenic control organoids. Taken together, our data demonstrate that patient-derived retinal organoids enable modeling of retinal degeneration and highlight the importance of CRB1 in early endosome maturation receptor recycling in the retina.

INTRODUCTION

In mammals, the Crumbs (CRB) protein family consists of CRB1, CRB2, and CRB3A, with the latter lacking a large extracellular domain. Mutations in the *CRB1* gene are responsible for retinal diseases such as retinitis pigmentosa (RP), Leber congenital amaurosis (LCA), and macular dystrophy (Nguyen et al., 2022). Cell adhesion and cell polarity protein complexes at the retinal outer limiting membrane (OLM), such as adherens junctions and CRB complexes, play a critical role in proliferation of retinal progenitor cells (RPCs) (Pellissier et al., 2013) as well as in cell adhesion (Quinn et al., 2018, 2019b; van de Pavert et al., 2007). The CRB complex is located at the subapical region adjacent to the adherens junctions between RPCs in the developing retina and at the subapical regions of Müller glial cells (MGCs), photoreceptor cells (PRCs), PRC-PRCs, and PRC-MGCs.

We have described previously that the levels of CRB-family proteins at the apical membrane in photoreceptors and MGCs are major determinants of the severity of the retinal phenotype in *Crb1*^{KO} (KO, knockout) and *Crb1*^{KO}*Crb2*^{cKO} (cKO, conditional KO) mice compared with controls. In contrast to CRB1, the CRB2 protein is present in photoreceptors and MGCs in both species (Quinn et al., 2019b; van Rossum et al., 2006). We showed that RP *CRB1* patient-derived retinal organoids expressing different variants of CRB1 proteins (*CRB1*$^{M1041T/M1041T}$; *CRB1*$^{Y631C/E995*}$; *CRB1*$^{M1041T/C948Y}$) have little cell polarity and cell adhesion markers on differentiation day 180 (DD180) and significantly lowered levels of variant CRB1 proteins, permitting local displacement of PRC columns (Boon et al., 2023; Quinn et al., 2019a). Loss of apical polarity has been linked previously to dysregulation of the endolysosomal system (Boon et al., 2023; Kraut and Knust, 2019; Lattner et al., 2019; Pocha et al., 2011; Zhou et al., 2011). The membrane vesicle trafficking system has been associated with many diseases but, to our knowledge, has not been studied in detail in human retinal organoids.

Numerous reports have previously demonstrated a link between loss of the CRB complex and activation of the Notch signal pathway (Laprise, 2011; Pellissier et al., 2013; Thompson et al., 2013). A direct interaction between

This is an open access article under the CC BY license (http://creativecommons.org/licenses/by/4.0/).

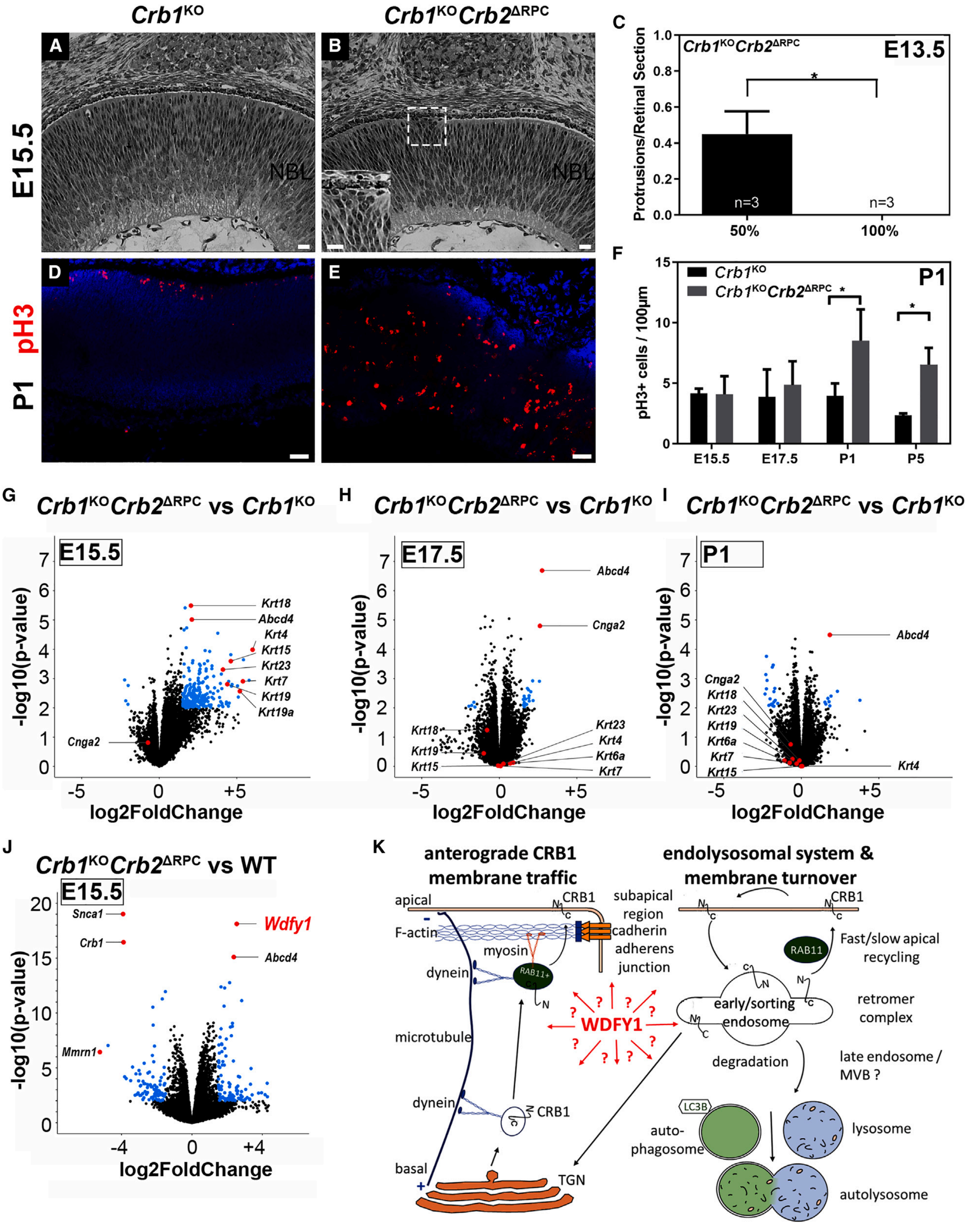

(legend on next page)

extracellular domains (ECDs) of NOTCH-CRB and CRB-CRB was observed in fruit fly and zebrafish models (Laprise, 2011; Nemetschke and Knust, 2016; Ohata et al., 2011; Zou et al., 2012). The ECD of CRB in the fruit fly or of CRB2 in the zebrafish stabilizes the NOTCH receptor at the apical membrane (Herranz et al., 2006; Lattner et al., 2019; Nemetschke and Knust, 2016; Ohata et al., 2011). Furthermore, the fruit fly CRB mutant protein (crb^{P13A9}) decreases NOTCH receptor localization at the plasma membrane; increases NOTCH intracellular domain (ICD) concentrations, which activates the NOTCH pathway; and expands the number of lysosomes in rhabdomeres (a cluster or photoreceptors and support cells in flies) compared with controls (Kraut and Knust, 2019).

Patients with the common RP *CRB1*-associated missense mutations ($CRB1^{Met1041Thr}$; $CRB1^{Cys948Tyr}$) have significantly lowered levels of CRB1 in the retina and a disturbed endolysosomal system, thus benefitting from gene supplementation therapy (Boon et al., 2023; Buck et al., 2021; Pellissier et al., 2015). In the present study, we identified target genes involved in the molecular mechanisms of the *CRB1* LCA-like phenotype in mice. We found that *Wdfy-1* is a gene overexpressed in the *CRB1* LCA-like mouse model. The WD repeat and FYVE domain-containing 1 (WDFY1) protein is found on the early endosome (Ridley et al., 2001). WDFY1 is localized on lysosomes, being part of an E3 ubiquitin ligase complex involved in selective autophagy, termed lysophagy, recognizing and removing damaged lysosomes (Teranishi et al., 2022). To analyze the role of CRB1 in endosome pathways, we studied induced pluripotent stem cell (iPSC)-derived human retinal organoids. We find a strong decrease in the level of apical CRB1 variant and apical NOTCH1 proteins at the OLM on DD180. We then trace back how the decreased levels of CRB1 variant proteins appear by investigating the trafficking and degradation of CRB1 proteins. We find that the significant decrease in CRB1 variant protein coincides with an increase of WDFY1 at the OLM and in the outer nuclear layer (ONL); repression of maturation of early endosomes (EEs), shown by an increase of EEA1 in EEs; a reduction of RAB11A+ recycling endosomes and VPS35/retromer vesicles; and an increase in degradative endolysosomal compartments in patient organoids. This study suggests that CRB1 is involved in EE/recycling endosome maturation.

RESULTS

RNA sequencing (RNA-seq) data from developing *Crb1*- and *Crb2*-deficient mouse retina reveals a molecular defect in the endolysosomal system

To identify the molecular mechanisms of developmental degradation in the retina lacking CRB1 and CRB2, we first examined the retina from $Crb1^{KO}$ ($Crb1^{KO}Crb2^{Flox/Flox}$) and $Crb1^{KO}Crb2^{\Delta RPC}$ cKO mice backcrossed into 100% C57BL/JOlaHsd mice. In agreement with previous findings in a mixed genetic background (Pellissier et al., 2013), our analyses of the retina from $Crb1^{KO}Crb2^{\Delta RPC}$ mice show that CRB1 and CRB2 are essential for proper retinal development, preventing disturbed retinal layering and loss of retinal function (Figures 1A, 1B, S1, and S2). Of note, the 100% C57BL6/JOlaHsd genetic background against the mixed C57Bl/6 and 129/Ola 50%/50% background shows a slightly milder retinal phenotype at foci (Figures 1C and S1). Some regions in the retina mimic the previously described milder heterozygous *Crb1* conditional homozygous *Crb2* ($Crb1^{WT/KO}Crb2^{\Delta RPC}$) cKO retinal phenotype, whereas other regions mimic the more severely affected double homozygote $Crb1^{KO}Crb2^{\Delta RPC}$ retina (Pellissier et al., 2013). The severity of the phenotype was still comparable with the mixed genetic background, considering retinal cell loss measured by retinal thickness (Figure S2H) and the loss of the retinal response to light flashes measured by electroretinography (Figures S2I–S2K) in 1-month-old mice (Pellissier et al., 2013). Previous studies on developing $Crb1^{KO}Crb2^{\Delta RPC}$ retinas on a mixed genetic background showed a transiently thicker retina with an increased number and mislocalization of late-born cells (rod photoreceptors, bipolar cells, MGCs, and late-born amacrine cells), increased cell proliferation and apoptosis, and dysregulation of the cell cycle, which severely impairs

Figure 1. mRNA transcript levels are marginally different in $Crb1^{KO}Crb2^{\Delta RPC}$ against $Crb1^{KO}$ retina

(A and B) Retinal morphology on plastic sections of $Crb1^{KO}$ and $Crb1^{KO}Crb2^{\Delta RPC}$ mice on a 100% C57/B6 genetic background at embryonic day 15.5 (E15.5). (A) $Crb1^{KO}$ retinas appeared to be unaffected, while (B) $Crb1^{KO}Crb2^{\Delta RPC}$ retinas had protrusions of neuroblast nuclei in the subretinal space (inset).

(C) Protrusions per retinal section in a 50% and 100% C57/B6 genetic background at E13.5.

(D–J) 100% C57/B6 genetic background. (D and E) Immunofluorescence labeling of pHH3+ nuclei (late G2 cell cycle and mitosis maker) on postnatal day P1 in (D) $Crb1^{KO}$ and (E) and $Crb1^{KO}Crb2^{\Delta RPC}$ retina. (F) Quantification of pHH3+ cells on E15.5, E17.5, P1, and P5. $^{*}p < 0.05$.

(G–J) All changed genes are indicated in blue circles ($p < 0.01$; log2(Fold Change) > 1.5). (G–I) $Crb1^{KO}Crb2^{\Delta RPC}$ against $Crb1^{KO}$ retina transcripts (run 1) on E15.5, E17.5, and P1. (J) $Crb1^{KO}Crb2^{\Delta RPC}$ compared with WT retina transcripts on E15.5 (run 2).

(K) Potential WDFY1 involvement in CRB1 protein trafficking (modified from Aguilar-Aragon et al., 2020).

Scale bars, 20 μm. WT, wild type; TGN, *trans*-Golgi network; MVB, multivesicular body. See also Figures S1 and S2.

retinal function in adult mice. It has been speculated that CRB1 and CRB2 suppress RPC proliferation by regulating mitogenic signaling pathways such as NOTCH, YAP, and Sonic Hedgehog (Pellissier et al., 2013). We also found upregulation of phosphohistone H3+ (pHH3+) mitotic cells on post-natal day 1 (P1) and P5 (Figure 1F), indicative of increased cell proliferation in the 100% C57BL/6JOlaHsd background.

We therefore performed RNA-seq on mouse retina to gain insights into these developmental changes at the transcriptional level. A comparison between *Crb1*^{KO}*Crb2*$^{\Delta RPC}$ and *Crb1*^{KO} retina, on 100% C57BL/6JOlaHsd, yielded only subtle persistent changes at the transcriptional level over time (Figures 1G–1I, respectively), despite significant differences in morphology (Alves et al., 2013a, 2013b). As an internal positive control, we used ATP-binding cassette, subfamily D (ALD), member 4 (*Abcd4*), which is substantially upregulated in mouse retina expressing the *Cre* recombinase fused to the *Chx10* promoter, with the *Abcd4* gene immediately adjacent to the *Chx10Cre* locus in the mouse genome. *Abcd4* expression and other genes were also validated by qPCR on P1 (Figure S2L).

To further examine potential transcriptional changes on embryonic day 15.5, we compared *Crb1*^{KO}*Crb2*$^{\Delta RPC}$ mice in a 100% C57BL/6JOlaHsd genetic background against wild-type C57BL/6JRccHsd mice. In this setting, we observed 40 differentially expressed genes (DEGs) compared with the previously 0 DEGs (adjusted p value < 0.01, log2(Fold Change) > 1.5). The *Crb1*^{KO}*Crb2*$^{\Delta RPC}$ mice on the C57BL/6JOlaHsd genetic background, because of mutations in the synuclein alpha (*Snca*), multimerin-1 (*Mmrn1*), and *Crb1* genes, do not express *Snca*, *Mmrn1*, or *Crb1* gene transcripts and express high levels of *Abcd4* because of the adjacent *Chx10Cre* transgene on chromosome 6. We used all 4 genes as negative and positive controls in gene expression profiling because the mice on the C57BL/6JRccHsd genetic background have no mutations in *Snca*, *Mmrn1*, or *Crb1* and express low levels of *Abcd4* (Gajovic et al., 2006). Analysis of the revealed upregulated expression of the WD repeat and FYVE domain-containing protein 1 (*Wdfy1*) gene, also known as FYVE domain-containing protein localized to endosomes (*Fens-1*), which encodes a phosphatidylinositol 3-phosphate binding protein that contains a FYVE zinc-finger domain and multiple WD-40 repeat domains (Figure 1J). The misregulation in EEs by WDFY1 is in line with the data from *Crb* fruit fly genetics (Kraut and Knust, 2019; Lattner et al., 2019; Pocha et al., 2011), which show protein aggregation and expression of immature apical surface markers linked to the endolysosomal system. The *CRB1* RP-like morphologic phenotype in the mouse, the surprisingly subtle differences on transcript levels (*Crb1*^{KO}*Crb2*$^{\Delta RPC}$ against wild-type mice) with *Wdfy1* being upregulated and potentially localizing on EEs, and single cell RNA sequencing (scRNA-seq) transcription profile studies on *CRB1* RP retinal organoids that suggested involvement of the endosomal system (Boon et al., 2023) prompted us to investigate whether variant CRB1 proteins affect the early endosomal system in *CRB1* RP-like human patient-derived retinal organoids (Figure 1K).

CRB1 patient retinal organoids bearing missense variations show reduced levels of CRB1 protein

To investigate the significantly reduced levels of variant CRB1 protein in *CRB1* patient retinal organoids, we generated isogenic controls (Boon et al., 2023). Isogenic controls were generated by homology-directed repair (HDR) of the *CRB1*^{M1041T} variant using CRISPR-Cas9 (Figure S3; Table S1). Wild-type CRB1 and variant CRB1 proteins (encoded by *CRB1*$^{M1041T/M1041T}$, *CRB1*$^{M1041T/C948Y}$, or *CRB1*$^{Y631C/E995*}$) were monitored by fluorescence over time using two different antibodies recognizing either an epitope on the short intracellular domain (ICD) of CRB1 or the first epidermal growth factor (EGF)-like domains of the extracellular domain (ECD). The CRB1-ECD/CRB1-ICD antibodies have a high CRB1 antigen specificity with little background observed in immuno-electron microscopy (EM) and immunohistochemistry on human donor cadaveric retinas (Pellissier et al., 2014). Both antibodies indicated clear CRB1 expression in the isogenic and control organoids on DD180 (Figures S4A–S4J). No CRB1-ECD/ICD overlapping signal was found on DD90/DD120 (Figures S4F and S4G), which is at the onset of CRB1 expression (DD120) found previously (Quinn et al., 2019a). As reported recently (Boon et al., 2023), a very weak variant CRB1 protein signal was observed at the OLM in the *CRB1* patient lines compared with the isogenic or control retinal organoids on DD180 (Figures S4A, S4B, and S5A–S5H).

The lower levels at the OLM of *CRB1*^{M1041T} (patient LUMC0116iCRB09), *CRB1*$^{M1041T/C948Y}$ (patient LUMC0128iCRB01), or *CRB1*$^{Y631C/E995*}$ (patient LUMC0117iCRB01) proteins in *CRB1* RP retinal organoids compared with control organoids are not due to changes in the *CRB1* transcripts. scRNA-seq on *CRB1* patients LUMC0116iCRB09 and LUMC0128iCRB01 versus their isogenic control retinal organoids on DD230 showed no statistically significant difference in *CRB1* variant versus *CRB1* wild-type mRNA expression, respectively (Boon et al., 2023). Unfortunately, lysates of wild-type DD180 or DD210 retinal organoids showed no specific CRB1 or CRB2 bands on western blots using anti-CRB1 or anti-CRB2, respectively. The reduction of CRB1 variant protein expression at the OLM suggests that patient-derived missense gene variations may alter the trafficking of CRB1-containing vesicles to the OLM or CRB1 turnover at the OLM.

CRB1/NOTCH1-ECD interact, and variant CRB1 reduces apical NOTCH1 expression

We found a strong reduction of the adhesion markers β-catenin, p120-catenin, N-cadherin, PAR3, MUPP1, and PALS1 at the OLM on DD180 (Quinn et al., 2019a). NOTCH signaling is involved in cell proliferation and cell adhesion. For example, NOTCH signals from the mouse retinal pigment epithelium contributed to NOTCH activation in adjacent RPCs (Ha et al., 2017). *Notch1* is essential for maintaining the RPC population (cycling cells) in the developing retina. Conditional knockdown of *Notch1* restricts the RPC subpopulation and confines the differentiation capacity of RPCs mainly to cone photoreceptors (Chen and Emerson, 2021). Also, the fruit fly CRB mutant protein (CRB^{P13A9}) decreases NOTCH receptor localization (Kraut and Knust, 2019).

NOTCH1 was expressed in the retinal pigment epithelium (RPE) at all time points, serving as a positive control (Figure 2A). At DD180, NOTCH1 expression was almost exclusively limited to Müller glial-villi at the OLM, co-labeled with CD44-Müller glial-villi marker and not with the photoreceptor/on-bipolar marker recoverin (Figures 2B and 2C). *CRB1* patient retinal organoids expressed lower levels of NOTCH1 at the OLM (Figures 2D–2F) and increased levels of NOTCH1 in the inner retina. Recently, a NOTCH-CRB interaction on the ECD was found by proximity ligation assay (PLA) in the fruit fly (Lattner et al., 2019). We observed an interaction of CRB1/NOTCH1 ECDs by PLA on human retinal organoids in patient and control retinal organoids (Figures 2G–2J). The interaction signal in patient retinal organoids was reduced at the OLM and increased in the ONL (Figures 2G–2J). The close proximity between NOTCH1 and CRB proteins is in accordance with the literature (Laprise, 2011; Nemetschke and Knust, 2016; Ohata et al., 2011; Zou et al., 2012).

Increased autophagy-lysosomal compartments in *CRB1* retinal organoids

Loss of CRB increased NOTCH and DELTA protein endocytosis in the fruit fly (Richardson and Pichaud, 2010), which increases ARL8+ lysosomes in rhabdomeres compared with controls in a pre-disease state (Kraut and Knust, 2019; Lattner et al., 2019). We next sought to determine whether endolysosomal vesicles (size, morphology) are affected by loss of apical CRB1 using transmission electron microscopy (TEM; Figure 3) and then identified and quantified them using light microscopy (Figure 4) on DD180.

Using TEM, we found few electron-dense degradative compartments/vacuoles in control organoids (Figures 3A–3D, few arrowheads) compared with many in patient organoids (Figures 3E–3G, arrowheads) and, surprisingly, some in gene-corrected *CRB1* heterozygous organoids (Figures 3A and 3B, arrowheads). Additionally, the electron-dense adherens junction/subapical region appeared to be elongated in some patient organoids (Figures 3E–3G, red arrows indicate adherens junction/subapical regions). Furthermore, some cell disruptions were detected in $CRB1^{M1041T/C948Y}$ organoids (Figure 3F, asterisks indicate nuclei above adherens junctions). Interestingly, we did not find definitive multivesicular bodies in any of the these specimens, suggesting that photoreceptors and MGCs may not utilize them during endosomal maturation.

The increase in electron-dense degradative compartments/vacuoles, seen using TEM, in patient retinal organoids was then quantified in DD180 retinal organoids using immunofluorescence microscopy. We hypothesized that the CRB1 variant is less efficiently shuttled to the OLM or, alternatively, that the turnover of CRB1 variant protein at the OLM is higher. However, we found no increase in the CRB1 variant in the inner/ONL, indicating little overall aggregation. Also, we observed minimal co-localization of the CRB1 variant with the general endolysosomal marker LAMP1 in the ONL/OLM (Figures S5A′–S5H′). We found more lysosomal LAMP1+ vesicles in the ONL, but not close to the OLM, in patient retinal organoids (Figures S5I and S5J). Then, we assessed the co-labeling and expression of the small GTPase lysosome kinesin adaptor ARL8A/B with CRB1 (Figure 4). ARL8A/B is found primarily on late lysosomes (Rosa-Ferreira and Munro, 2011). Also, ARL8A/B takes part in anterograde transport of mature lysosomes to the cell and co-mediates transport of endocytic cargo to lysosomes (Farfel-Becker et al., 2019; Rosa-Ferreira and Munro, 2011). ARL8+ lysosomes have been found previously to accumulate CRB variant proteins in the fruit fly (Kraut and Knust, 2019). We, however, also found little co-labeling of variant CRB1 with ARL8A/B (Figures 4A–4H). Similar to LAMP1, the area of total ARL8A/B (lysosome/late endosome) puncta was increased in the ONL of patient organoids (Figures 4K and 4L), implying accumulation of degradative compartments but not accumulation of CRB1 variant protein.

We also examined CRB1 variant protein being co-labeled with classic autophagy markers, such as the ubiquitin-binding protein adapter p62 and the microtubule-associated proteins 1A/1B light chain 3B (LC3B) in the ONL/OLM. The autophagy cargo adapter p62 can transport substrates to the lysosome that have been tagged; e.g., by LIR (LC3-interacting motif for degradation) (Overhoff et al., 2021). p62 has an important role in selective autophagy of organelles and ubiquitinated misfolded/damaged proteins (here potentially the CRB1 variant protein). Further, p62 has been linked to selective stress-response-induced autophagy and proteostasis and is extensively implicated in selective autophagy (Overhoff et al., 2021; Pradhan et al., 2019; Wooten et al., 2008). We found a strong increase in LC3B and p62 presence in *CRB1* patient retinal organoids in the ONL and at the OLM (Figures 5 and S6). The increase may be

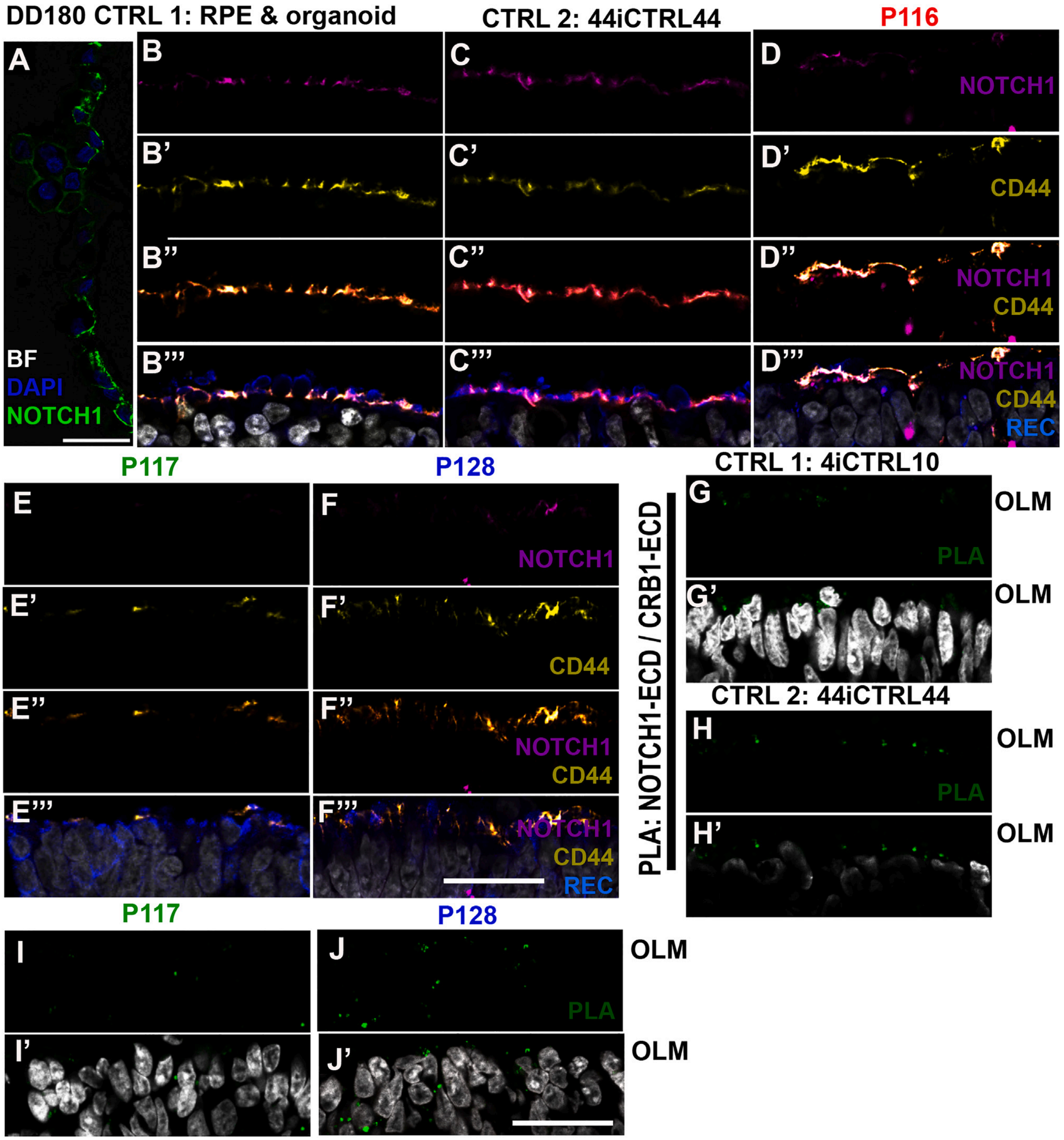

Figure 2. Apical NOTCH1 is lost in *CRB1* patient retinal organoids

(A) CTRL1 (LUMC004iCTRL10) RPE expresses NOTCH1 on DD180 (BF, bright field; to indicate pigments in the RPE).

(B–F) CTRL1 (B), CTRL2 (LUMC044iCTRL44) (C), patient P116 (LUMC0116iCRB09) (D), patient P117 (LUMC0117iCRB01) (E), and patient P128 (LUMC0128iCRB01) (F). (A–F) NOTCH1 is expressed specifically in DD180 Müller glial apical villi (magenta, NOTCH1; yellow, CD44; blue, recoverin; gray, DAPI).

(G–J) PLA of NOTCH1-ECD and CRB1-ECD (green signal) shows interaction at CTRL OLM but reduced interaction at patient OLM with increased localization in patient ONL.

Scale bars, 25 μm.

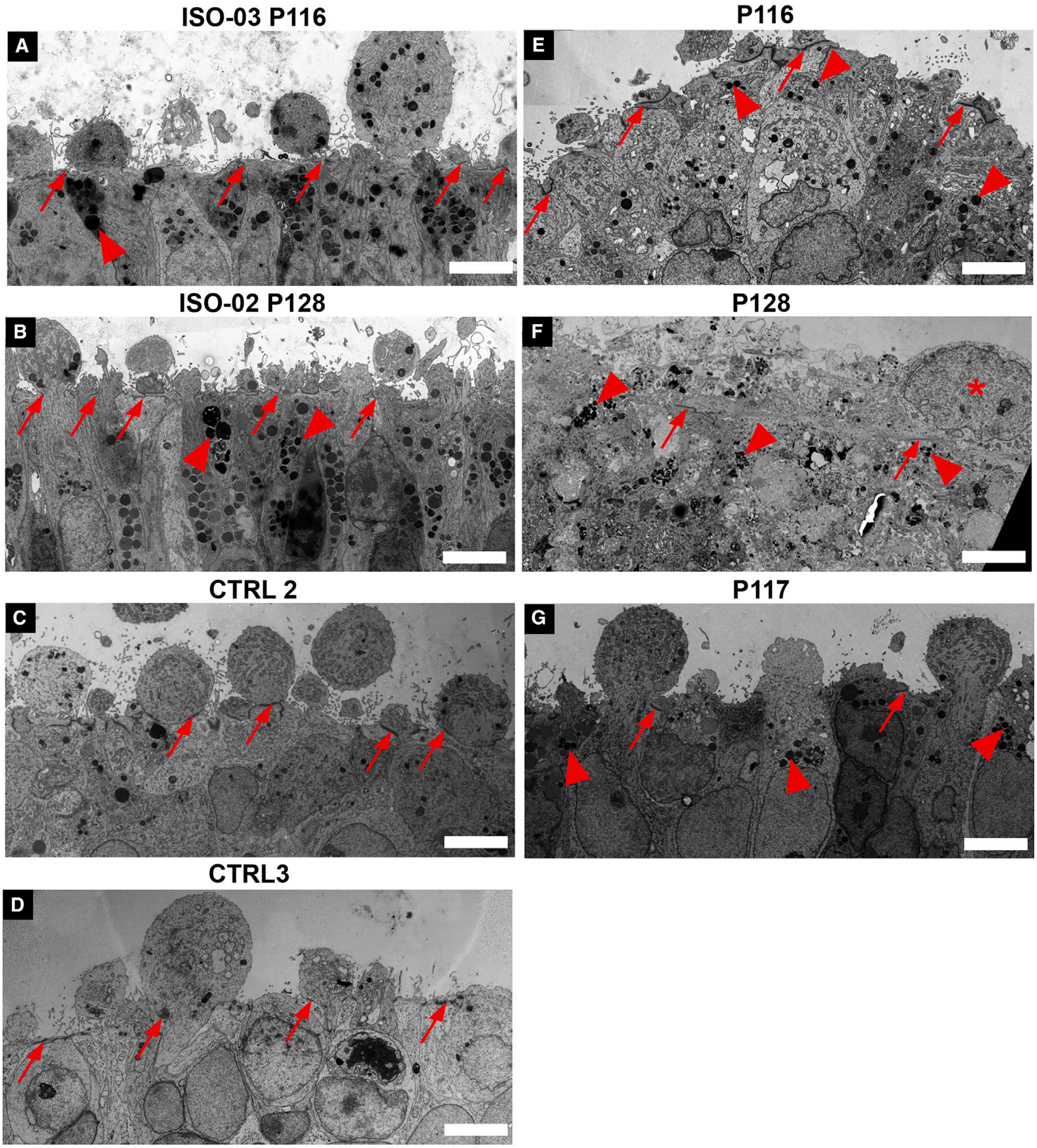

Figure 3. High-resolution TEM imaging of retinal organoids on DD180

Shown are electron-dense OLM (red arrows), electron-dense degradative compartments/vacuoles (arrowheads), and nuclei above the OLM (asterisks).

(A and B) Gene-corrected lines (ISO-03 P116, iso3LUMC0116iCRB09; ISO-02-P128, iso2LUMC0128iCRB01).

(C and D) CTRL lines (CTRL2, LUMC0044iCTRL44; CTLR3, LUMC080iCTRL12).

(E–G) Patient lines P116, P128, and P117 (LUMC0116iCRB09, LUMC0128iCRB01, and LUMC0117iCRB01, respectively).

Scale bars, 5 μm. Related to Figure S3.

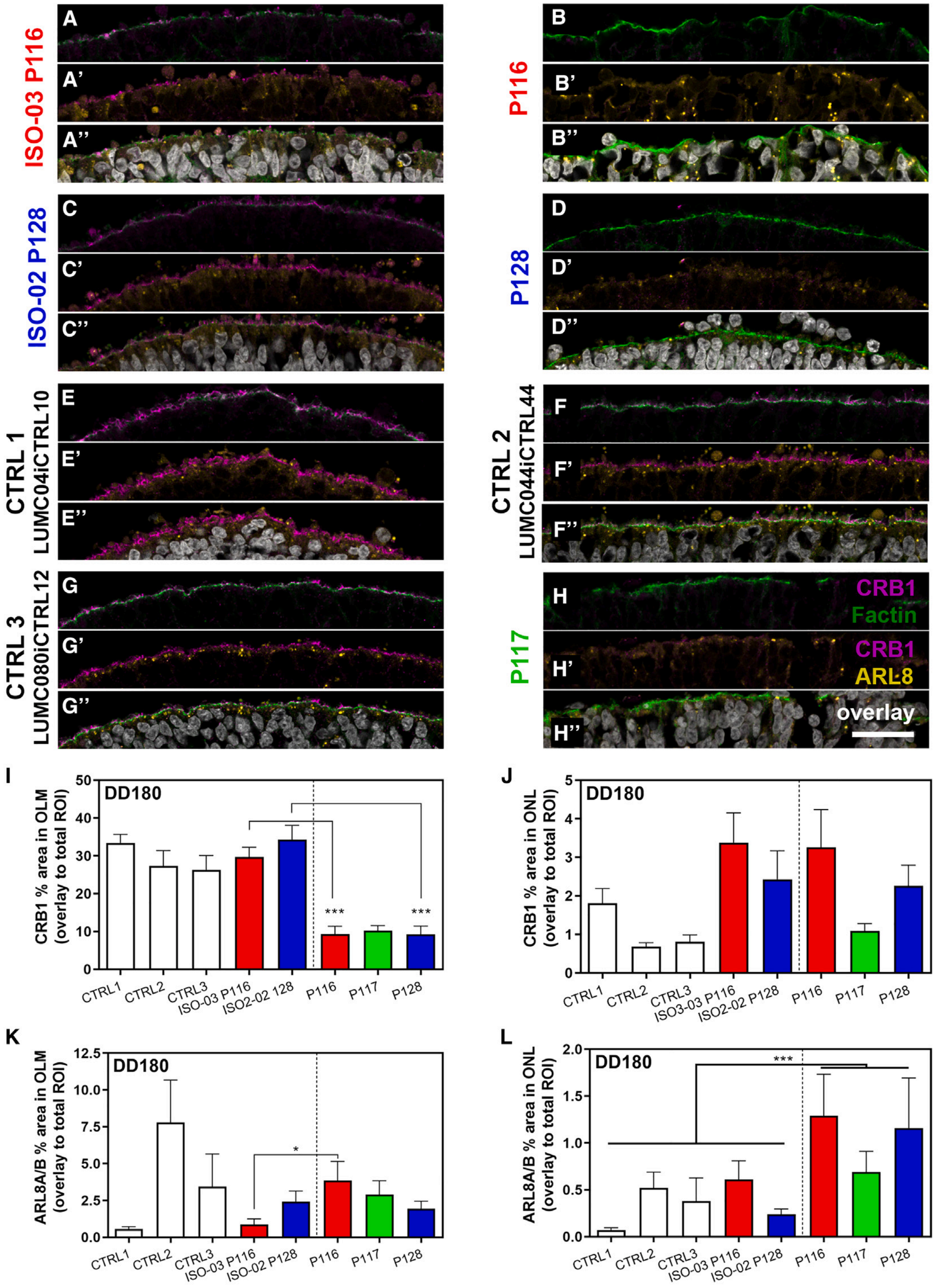

Figure 4. The immunofluorescence signal of CRB1 labeling is reduced in *CRB1* patient retinal organoids at the OLM

(A–H) Triple staining of CRB1-ICD (AK2 antibody, magenta), phalloidin (filamentous actin [F-actin], green), and ARL8A/B (yellow).

(I and J) CRB1 fluorescence signal measured by puncta in the OLM and ONL.

(K and L) CRB1 fluorescence signal measured by puncta in the OLM and ONL.

(legend continued on next page)

related to changes in autophagic flux. We added BafA1 to inhibit acidification of endolysosomes by blocking the vacuole-ATPase (H^+/Ca^{2+} antiporter), which is required for fusion of lysosomes with autophagosomes to form auto-lysosomes and, hence, for degradation of proteins targeted to lysosomes. We incubated retinal organoids with BafA1 (500 nM) for 6 h and extracted the lysates. BafA1 induced a robust block of lysosome-autophagosome fusion on individual organoids (Figures 5I–5K, S6C, and S6D). We found more autophagosome markers (LC3-II) in *CRB1* patient retinal organoids and decreased autophagic flux (Figures 5J, 5K, S6G, and S6H), pointing toward a blockage of autophagic vesicle removal. We also found little variant CRB1 expression and co-labeling of CRB1 with p62, LC3B, or ARL8A/B. Accordingly, CRB1 variant protein might be either expressed at low levels (Figure S4), unstable, degraded in the inner/ONL with little variant CRB1 reaching the OLM, or reach the OLM but show high turnover because of abnormal vesicle sorting. Taken together, we show by immunohistochemistry that CRB1 variant proteins are expressed at more than 4-fold lower levels than CRB1 protein in isogenic controls (Figure 4I). The PLA between CRB1 and NOTCH1 shows some remaining CRB1 variant signal at the OLM and mislocalization within the ONL (Figures 2I and 2J), suggesting that the CRB1 variant protein is translated and transported by the endosomal system to the OLM.

Decreased levels of CRB1 reduce the pool of recycling endosomes

To determine where the decrease in autophagic flux originates, we investigated the expression of RAB11A. RAB11A is a small GTPase regulating formation of transport vesicles, recycling endosomes, and overall intracellular membrane trafficking (Kakar-Bhanot et al., 2019). The turnover of CRB and CRB membrane integration in the fruit fly is also determined by RAB11 (Aguilar-Aragon et al., 2020; Figure 1K). We found a strong signal of RAB11A co-labeling with F-actin in control organoids at the OLM (Figures 6A, 6C, and S7A–S7C). RAB11A/F-actin co-labeling was strongly reduced in *CRB1* RP patient retinal organoids (Figures 6B, 6D, and S7D), with RAB11A yielding 50% less signal at the OLM (Figure 6P). We further investigated the early endosomal population (EEA1+), late endolysosomal population (RAB7+), retromer-positive recycling compartments (RAB7+, VPS35+), and late endolysosomal population (RAB7+, ARL8A/B+). We found little difference in RAB7 expression between patient and control retinal organoids (Figures 6E–6H, 6I–6L, 6M, and S7E–S7H). However, less RAB7 co-labeled with VPS35 (Figures 6E‴–6H‴, and S7E‴–S7H‴), while co-labeling with ARL8A/B increased in patient retinal organoids (Figures 6 E⁗–6H⁗, and S7E⁗–S7H⁗). Furthermore, the EE population was increased in patient retinal organoids (Figures 6I′–6L′, 6N, and S7I). Finally, we investigated WDFY1 protein expression in retinal organoids, which we found to be upregulated at the transcript level in $Crb1^{KO}Crb2^{\Delta RPC}$ compared with wild-type mouse retina (Figure 1J). Recently, WDFY1 was identified as part of the CUL4A-DDB1-WDFY1 E3 ubiquitin ligase complex involved in selective autophagy, called lysophagy (Teranishi et al., 2022). We found little co-labeling with EEs in control organoids (Figure 7). Moreover, WDFY1 does not necessarily have to reside on EEs but could instead be located in the cytosol, from where it is most likely scrapped by the phospholipid PtdIns(3)P, which is actively recruited by EEA1 (Gaullier et al., 1998; Ridley et al., 2001). We noted that EEs in patient *CRB1* organoids expressed more EEA1 (Figures 7B, 7D, and 7F) and partially co-localized with WDFY1/EEA1 (Figures 7B″, 7D″, and 7F″), whereas some of the WDFY1 co-localized with the lysosomal marker Cathepsin D (Figures 7B‴, 7D‴, and 7F‴), indicating that dysregulated endosomes may recruit more WDFY1. An increase in WDFY1 fluorescence signal was found in the ONL and OLM (Figures 7G and 7H). The statistical analysis of WDFY1 protein levels suggested no statistical significance when comparing the two *CRB1* patient retinal organoids combined with their corresponding isogenic controls. Interestingly, when comparing all of the controls (control-1, control-2, control-3, ISO-03-P116, and ISO-02-P128) with all *CRB1* retinal organoids (P116, P117, and P128), we measured a statistically significant difference ($p < 0.001$), suggesting an increase in levels of WDFY1 protein at the OLM and ONL.

DISCUSSION

It is still challenging to predict the progression of the spectrum of *CRB1*-associated retinal dystrophy phenotypes in *CRB1* patients based on the genotype variants. A thorough knowledge of the clinical spectrum, acquired through detailed phenotyping and natural history studies (Nguyen et al., 2022), is important for optimal counseling and patient selection for interventional studies. Here we address three common RP *CRB1*-associated variants on (1) their protein

Scale bars, 25 μm. Each data point in the graphs represents individual organoids, of which an average was taken of 3 representative images. The SEM is derived from these averages. The numbers of individual organoids per condition (CTRL1, CTRL2, CTRL3, ISO-03 P116, ISO-02 P128, P116, P117, and P128) in (I) and (J) are 9, 9, 9, 10, 10, 12, 9, and 13, respectively, and for (K) and (L) 7, 8, 3, 8, 8, 12, 6, and 8, respectively (from at least two independent organoid batches). Statistical analysis: *$p < 0.05$, ***$p < 0.001$. Related to Figures S4 and S5.

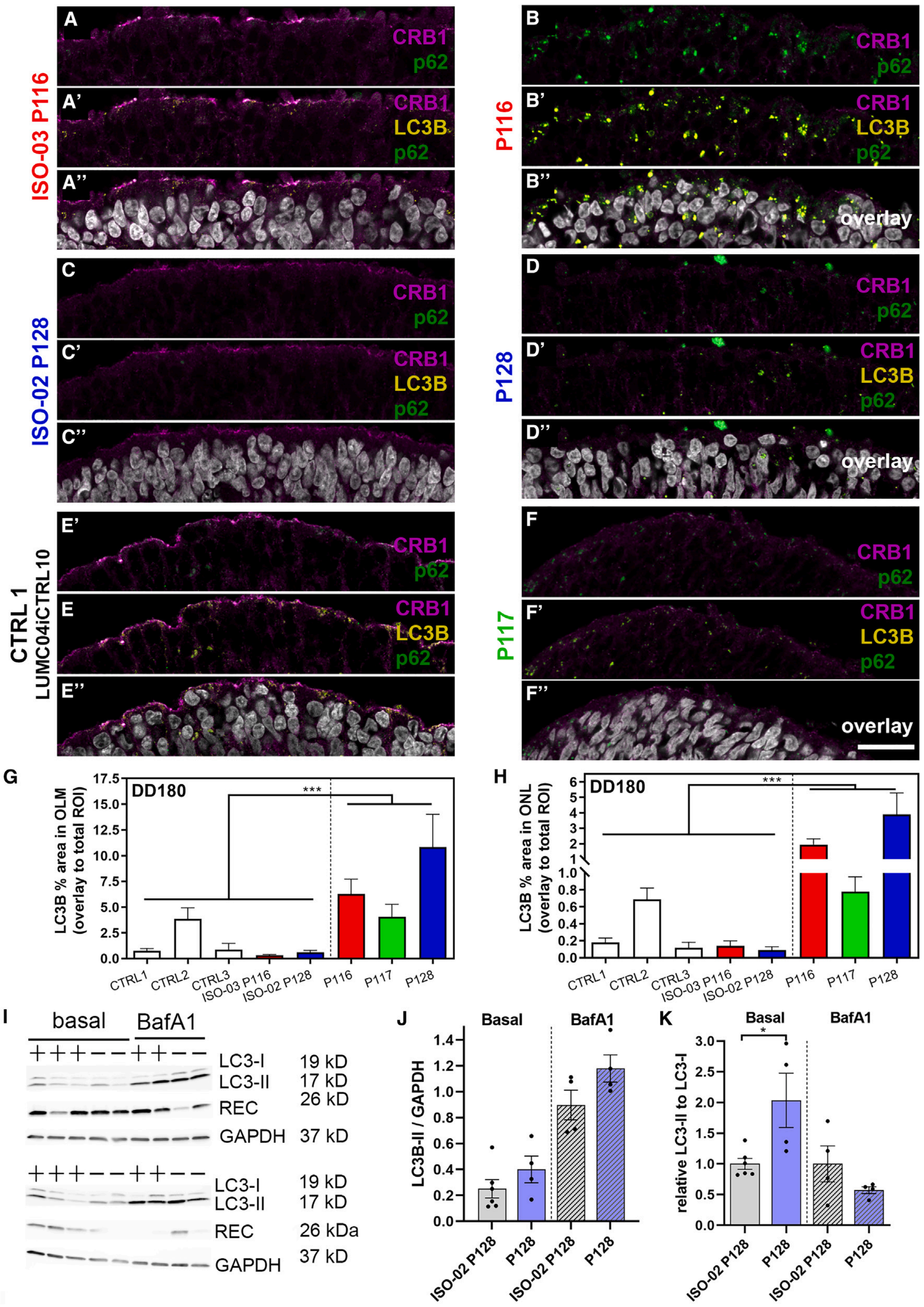

(legend on next page)

expression profile in the retina (significantly lowered levels of variant CRB1) and (2) the effects of the three CRB1 variants on apical EE maturation. Both results have implications for treatment (e.g., supporting EE maturation or *CRB* gene supplementation) and provide information for patients with one of the *CRB1* variants, resulting in a better understanding of the etiology.

We do find strong upregulation of *Wdfy1* in $Crb1^{KO}$ $Crb2^{\Delta RPC}$ compared with wild-type mice. The function of *Wdfy1* is still elusive in many cell types but has been linked to EEs (Ridley et al., 2001) involved in selective autophagy, termed lysophagy, recognizing and removing damaged lysosomes (Teranishi et al., 2022). Of interest is that the previously described *Crb1* KO mice as well as the *CRB1* patient retinal organoids described here have significantly reduced levels of but do not completely lack CRB proteins in MGCs. *Crb1* KO mice still have substantial levels of CRB2 in MGCs and photoreceptors, whereas *CRB1* patient retinal organoids still have substantial levels of CRB1 variant proteins plus CRB2 in MGCs and photoreceptors. In the scRNA-seq dataset on *CRB1* patient retinal organoids, *WDFY1* was not among the DEGs in MGCs or photoreceptors on DD230. However, significant changes in the endolysosomal system were detected that could be restored to isogenic control levels by overexpressing recombinant human CRB1 or CRB2 protein (Boon et al., 2023). Interestingly, we do detect increased levels of the early endosomal WDFY1 protein in *CRB1* patient retinal organoids compared with five independent control organoids. Future studies are needed to show the relevance of changed intracellular localization of WDFY1 in *CRB1* retinal organoids. Because WDFY1 has similar protein domains as those present in the endosomal protein EEA1, it is of interest to further study the potential link between decreased levels of variant CRB1 at the OLM and reductions in the levels of RAB11A and VPS35 as well as the increase in early endosomal WDFY1.

Retinal organoids express CRB1 and CRB2 protein at the OLM (Quinn et al., 2019a). The at least 4-fold reduced levels of CRB1 variant proteins in *CRB1* patient retinal organoids carrying missense mutations are not associated with reduced levels of *CRB1* mRNA transcript (Boon et al., 2023). In the scRNA-seq analysis, we find no differences in the level of expression of *CRB1* patient mRNA transcripts in MGCs or photoreceptors compared with the levels of *CRB1* mRNA transcript in corresponding isogenic controls. However, levels of *CRB2* transcripts in isogenic controls were lower in MGCs than in photoreceptors. Interestingly, scRNA-seq studies on cadaver human retina also reported a significantly (~20-fold) lower level of *CRB2* mRNA transcripts in MGCs than in photoreceptors (https://www.proteinatlas.org/ENSG00000148204-CRB2/single+cell+type). In several mouse studies, it has been shown that CRB2 and CRB1 proteins have overlapping physiological functions in MGCs and that their loss in the retina results in similar phenotypes (Alves et al., 2013a; van de Pavert et al., 2004, 2007). We hypothesize that low levels of CRB2 in combination with low levels of variant CRB1 at the OLM of MGCs cause disruption of the endolysosomal system. There is an urgent need for a better understanding of the retinal phenotype of human retina with mutations in the *CRB1* gene, especially because variations in *CRB1* cause variable retinal phenotypes, including RP in young children as well as LCA in newborns. Interestingly, on diagnostic spectral-domain optical coherence tomography (SD-OCT) imaging of retinas of *CRB1* patients with LCA, the retina appears to be thickened. Such thickened retina is not observed in *CRB1* patients with late onset of RP or in our RP *CRB1* patient retinal organoids. Surprisingly, in patients, the same set of mutations can result in RP or LCA. In mice, onset of LCA or RP is dependent on the levels of CRB1 or CRB2 proteins in MGCs and photoreceptors (Boon et al., 2020; Buck et al., 2021). In mice, the levels of CRB1 and CRB2 proteins in MGCs were determined to be of near-equal functional relevance (Buck et al., 2021; Quinn et al., 2019b). The situation in the adult human retina might be different because the levels of *CRB2* mRNA transcripts in MGCs are ~20-fold lower than in photoreceptors. In analogy to our findings in *Crb* mice, we hypothesize that the levels of human CRB2 functional protein units in MGCs might be substantially lower than the CRB1 functional protein units in MGCs and therefore cannot sufficiently compensate when CRB1 variant protein levels are reduced in *CRB1* retina. Lowering the levels of CRB1 variant proteins in MGCs of *CRB1* patients might cause a shortage of CRB proteins at

Figure 5. More degradative vesicles/compartments are present in *CRB1* patient retinal organoids

(A–F) Immunofluorescence triple staining of CRB1-ICD (AK2 antibody, magenta), p62 (green), and LC3B (yellow).

(G and H) LC3B localized more in the ONL and OLM layers in *CRB1* RP retinal organoids.

(I–K) Western blots of individual organoid lysates (plus symbol, isogenic 2 line (iso02-128iCRB01); minus symbol, patient 3 (line LUMC0128CRB01) stained for LC3B (LC3-I and LC3-II; 19/17 kD; recoverin for photoreceptors (26 kD), and GAPDH (housekeeping CTRL, 37 kD). Autophagic flux was decreased in *CRB1* patient retinal organoids.

Scale bars, 25 μm. Each data point in the graphs in (J) and (K) represents individual organoids, of which an average was taken. The SEM is derived from these averages. The numbers of individual organoids in (G) and (H) per condition (CTRL1, CTRL2, CTRL3, ISO-03 P116, ISO-02 P128, P116, P117, and P128) are 7, 6, 9, 8, 9, 10, 11, and 10, respectively (from at least two independent organoid batches). Statistical analysis: *$p < 0.05$, ***$p < 0.001$. Related to Figures S5, S6, and S8.

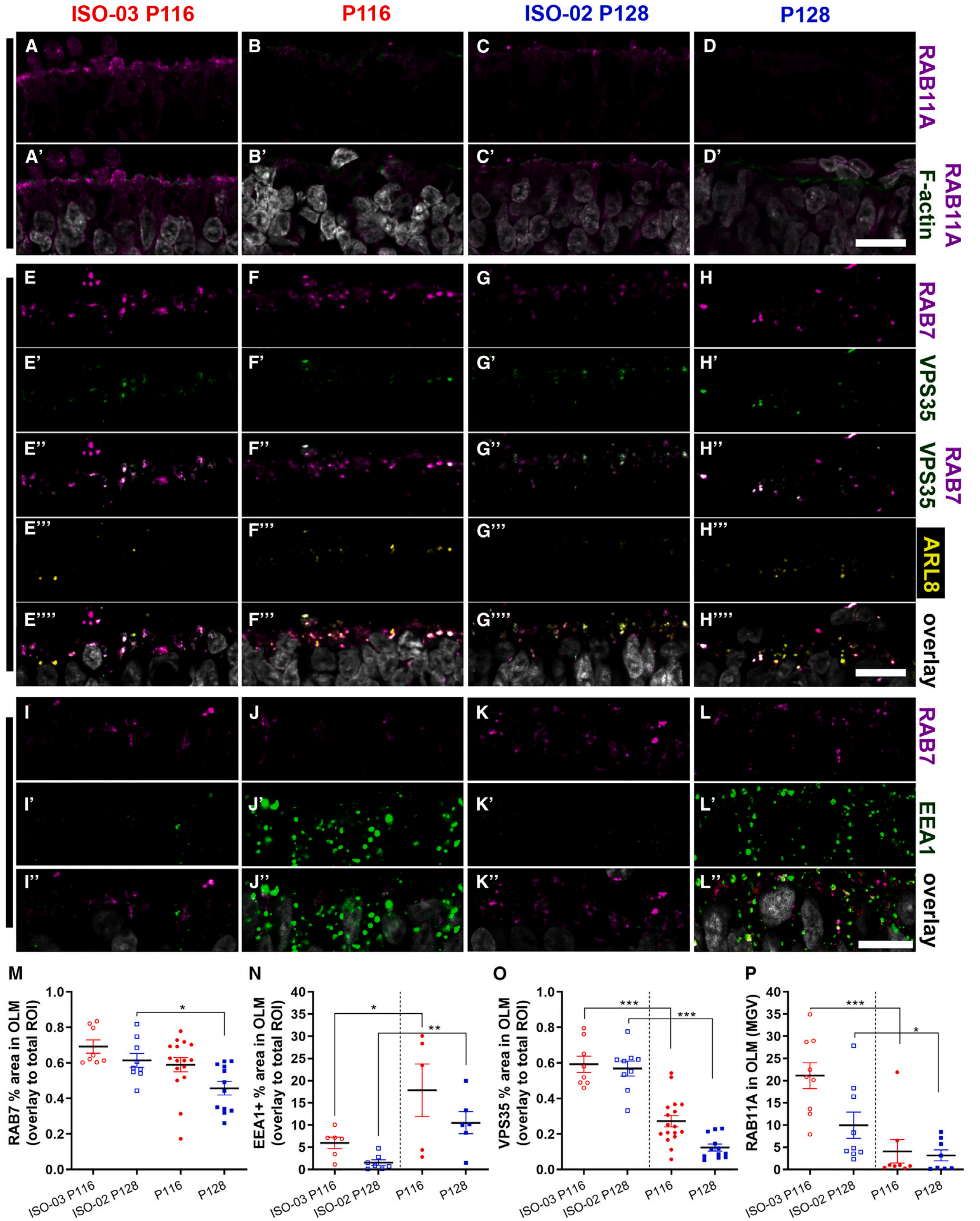

(legend on next page)

the OLM in MGCs that cannot be compensated for by the relatively low levels of CRB2 proteins in MGCs. One result of lowered CRB proteins might be disturbances in the RAB11A recycling of several apical plasma membrane proteins close to the OLM, here exemplified by lower levels of NOTCH1 concomitant with the lower levels of variant CRB1.

It remains to be determined what controls CRB1 protein expression, localization, and turnover rate at the OLM to determine the progression of retinal degeneration in *CRB1*-associated retinal dystrophy phenotypes in *CRB1* patients. In the present study, we focused on the endolysosomal system because we found an increase in endosome-associated *Wdfy1* gene expression in $Crb1^{KO}Crb2^{\Delta RPC}$ mice and degradation-associated proteins on endolysosomes (ARL8/LAMP1/p62/LC3 puncta) in patient retinal organoids. Besides western blotting for LC3-I and LC3-II, we also tried western blotting for anti-CRB1 and anti-CRB2 and against endosomal proteins. Unfortunately, whereas the total retinal organoid lysates showed LC3B and recoverin protein bands on western blots, we could not purify MGCs or photoreceptors from the limited number of isogenic and *CRB1* retinal organoids to detect CRB1, CRB2, NOTCH1, or endosomal proteins on western blots with the available antibodies.

We paid particular attention to the retromer-positive and early recycling compartments. The retromer complex (SNX1/2, SNX5/6, VPS26, VPS29, and VPS35) on early/late endosomes and the budded off vesicles/recycling endosomes (e.g., RAB11A, TfR1) work in synergy for efficient protein recycling in cells (Trousdale and Kim, 2015) and are thus potentially essential parameters controlling CRB1 protein turnover. What is more, the retromer proteins VPS26/VPS35 localize on early and late endosomes in fruit fly photoreceptors, and loss of VPS26 or VPS35 considerably increases degradative compartments (Wang et al., 2014). Recycling of CRB2 at the apical membrane is determined by binding of its ICD to VPS35 of the retromer complex on EEs (Pocha et al., 2011). CRB protein recycling also depends on RAB11 (Aguilar-Aragon et al., 2020). The ICD of CRB2A on RAB11A+ recycling endosomes takes part in regulating the cell cycle exit of RPCs and maintaining NOTCH1 at the OLM (Clark et al., 2020). RAB11 is known to mediate release of the early endosomal cargo (e.g., NOTCH1, CRB1) to the endocytic recycling compartment when peripheral EEs experience a temporary peak in PtdIns(3)P integration on their vesicular membrane (Campa et al., 2018; Gaullier et al., 1998). Potentially poor RAB11A-initiated release in *CRB1* patient retinal organoids may also amass WDFY1 mediated by its FYVE domain binding to PtdIns(3)P (Ridley et al., 2001). The presence of PtdIns(3)P in the membrane is vital for shifting WDFY1 from a soluble and cytosolic form to an EE or vesicle membrane-bound form. The actual function and normal equilibrium of cytosolic versus membrane-bound WDFY1 are not known. In line with this, our data on *CRB1* variant patient retinal organoids show reduced retromer-associated late endosomes (VSP35+/RAB7+), an increase in EEs destined for degradation (EEA1+/WDFY1+), a decrease in recycling endosomes (RAB11A+), and concomitant expansion of degradative endolysosomal compartments (ARL8/LAMP1/LC3-II/p62+). We propose that, in *CRB1* patient retinal organoids, disturbed EEs are trafficked to degradative compartments, lowering CRB1 protein expression at the OLM.

EXPERIMENTAL PROCEDURES

Resource availability

Corresponding author

Further information and requests for resources and reagents should be directed to and will be fulfilled by the corresponding author, Jan Wijnholds (j.wijnholds@lumc.nl).

Figure 6. Dysregulation of the endolysosomal system in *CRB1* patient organoids

(A–L) Immunofluorescence stainings, with the antibodies indicated on the right sides of the panels, on isogenic CTRL organoid lines 1 and 2 (A, C, E, G, I, and K) related to patient organoid lines 1 (LUMC0116iCRB09) and 3 (LUMC0128iCRB01) and lines 1 and 3 (B, D, F, H, and J–L).

(A–D) Recycling endosomes (RAB11A, magenta) and phalloidin (F-actin, OLM, green). The OLM in retinal organoids is stained by F-actin.

(E–H) Late endosomes (RAB7, magenta), retromer complex (VPS35, green), and endolysosomes (ARL8A/B, yellow).

(I–L) Late endosomes (RAB7, magenta) and EEs (EEA1, green).

(M–P) Quantification of the fluorescence signal of EEs (EEA1), late endosomes (RAB7), retromer (VSP35), and recycling endosomes (RAB11A) in the OLM.

(M) RAB7 in the OLM.

(N) EEA1 in the OLM.

(O) VPS35 in the OLM.

(P) RAB11A in the OLM.

Scale bars, 10 μm. Each data point in the graphs represents individual organoids, of which an average was taken of 3 representative images. The SEM is derived from these averages. The numbers of individual organoids per condition (ISO-03 P116, ISO-02 P128, P116, and P117) for (M) and (O) are 8, 9, 16, and 12, respectively; for (N) 6, 7, 5, and 6, respectively; and for (P) 9, 9, 8, and 8, respectively (from at least two independent organoid batches). Statistical analysis: *$p < 0.05$, **$p < 0.01$, and ***$p < 0.001$. Related to Figure S7.

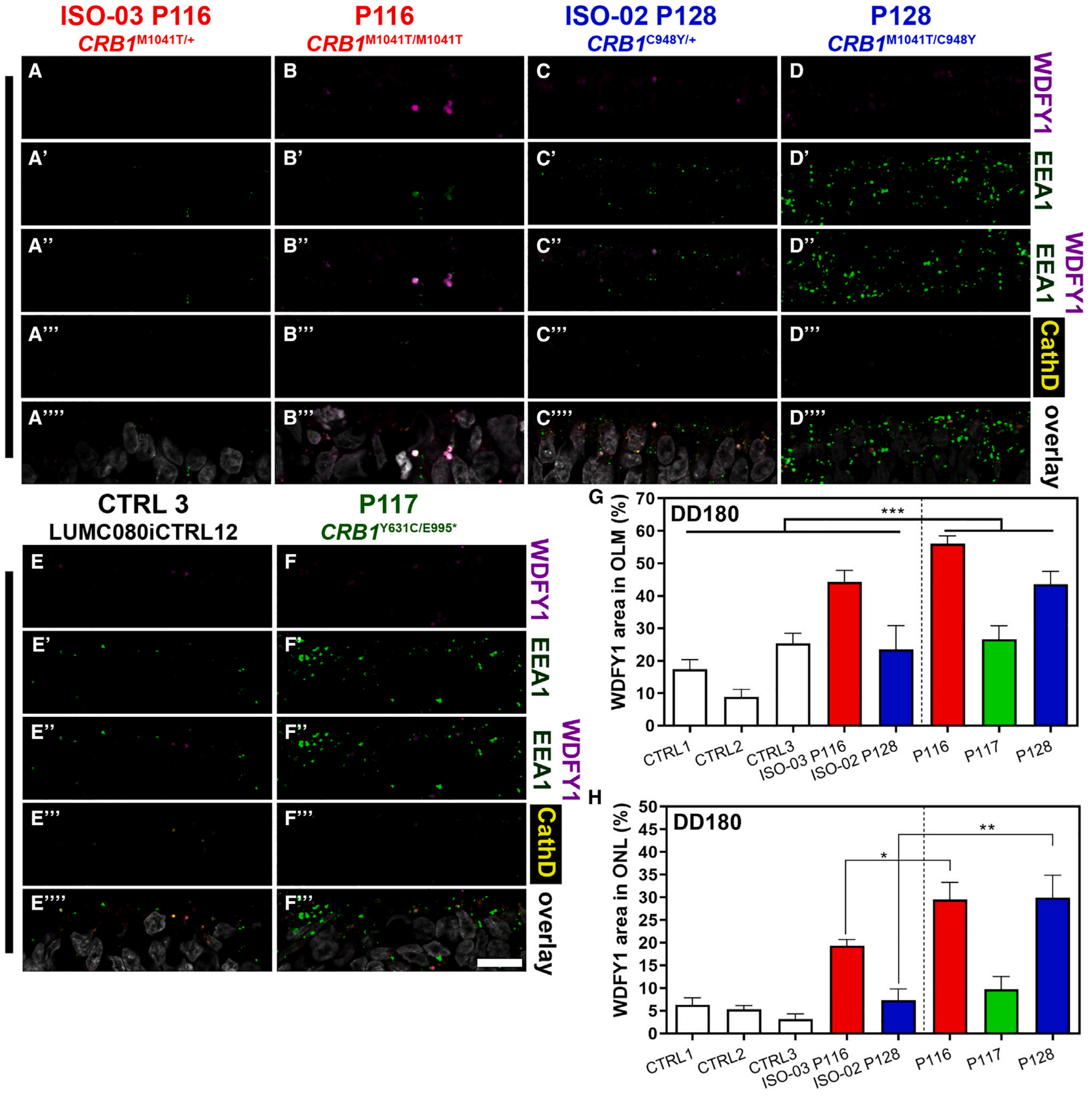

Figure 7. Increased levels of WDFY1 in EE s of patient *CRB1* patient retinal organoidsAll organoids are shown at DD180

(A–F) Immunofluorescence labeling of WDFY1 (magenta), EEA1 (green), and Cathepsin D (yellow). The overlay is shown in white (A''''–F'''').

(G and H) Semiquantification of the fluorescence signal of WDFY1.

(G) WDFY1 particle area/total OLM area in percent.

(H) WDFY1 particle area/total ONL area in percent.

Scale bars, 10 μm. Each data point in the graph represents individual organoids, of which an average was taken of 3 representative images. The SEM is derived from these averages. The numbers of individual organoids per condition (CTRL1, CTRL2, CTRL3, ISO-03 P116, ISO-02 P128, P116, P117, and P128) in (G) and (H) are 8, 9, 9, 10, 8, 10, 11, and 14, respectively (from at least two independent organoid batches). Statistical analysis: $^{*}p < 0.05$, $^{**}p < 0.01$, and $^{***}p < 0.001$.

Materials availability

Materials and additional details can be made available by the corresponding author upon reasonable request.

Data and code availability

RNA-seq data are available at the NCBI Gene Expression Omnibus database (GEO: GSE239456).

Animals

Procedures concerning animals were performed with permission of the ethics committee of Leiden University Medical Center and the animal experimentation committee of the Royal Netherlands Academy of Arts and Sciences (KNAW) under permit NIN 12.105.

RNA-seq

Sequencing was performed using Life Technologies SOLiD5500 with single-end, 50-bp reads. Two separate runs were performed. Run 1 contained 30 mice; n = 5 *Crb1*^{KO}*Crb2*$^{F/F}$ (*Crb1*^{KO}) and n = 5 *Crb1*^{KO}*Crb2*$^{\Delta RPC}$ (Tg) per development time point of the mouse embryo at embryonic day 15.5 (E15.5), E17.5, and P1. Run 2 contained 9 mice; n = 4 *Crb1*^{KO}*Crb2*$^{\Delta RPC}$ mice (C57BL/6JOlaHsd) and n = 5 healthy mice (C57BL/6JRccHsd = wild type [WT]) at E15.5. Reads were aligned against mm10 using the "whole.transcriptome.frag" workflow with the bamgen.mqv.threshold set to 20 (Lifescope v.2.5). Counts were obtained using the gene transfer format (GTF) output format as supplied by Lifescope (transformation of refGene.txt downloaded from the University of California Santa Cruz [UCSC] Table Browser on June 25, 2014) within this workflow.

Differential expression analysis

The analysis was performed as described previously (Pellissier et al., 2013).

Cell culture

We previously described the three male *CRB1* RP-patient human iPSC (hiPSC) lines (patient lines 1–3: LUMC0116iCRB09 or P116, LUMC 0117iCRB01 or P117, LUMC0128iCRB01 or P128) and three control lines (CTRL 1–3: LUMC0004iCTRL10 (hPSC^{reg} name: LUMCi029-B), LUMC0044iCTRL44, LUMC0080iCTRL12; Table S1; (Quinn et al., 2019a). Testing for pluripotency of hiPSC lines was performed for at least 3 clones per mutated line or isogenic CTRLs (Figures S3C, S3F, and S3G; see Table S2 for antibody concentrations).

CRISPR-Cas9-based gene repair of the hiPSC lines LUMC0116iCRB09 and LUMC0128iCRB01

The *CRB1* variant c.3122T>C (exon 9) was repaired by CRISPR-Cas9 ribonucleoprotein (RNP)-mediated homologous recombination. Sequences of the single guide RNA (sgRNA) and the repair template single-stranded oligodeoxynucleotides (ssODN) are provided in Figures S3D and S3E.

Retinal organoid differentiation

The differentiation of the hiPSCs is described in the supplemental information.

Fluorescence quantification in regions of interest (ROIs)

All organoids (7 μm sectioned) imaged for fluorescence semiquantification were stained (Table S2) with the same antibody mix at the same time, imaged in one confocal microscopy session, and included a negative CTRL (no primary antibody added; see supplemental information).

Conjugation of NOTCH1 and CRB1 antibody to plus and minus oligonucleotide probes

We used the Duolink Probemaker Set (Sigma-Aldrich) to conjugate two same-species antibodies (mouse anti-NOTCH1 and mouse anti-CRB1 ECD antibodies) to the plus or minus oligonucleotide probes as described in the protocol.

PLA

For the PLA, the Duolink *In Situ* Detection Reagents Kit Green (Sigma-Aldrich) was used. Slides with sliced organoids were washed, and the tissue slices were circled with a hydrophobic pen. Slides were blocked with one drop of blocking solution from the Duolink PLA probe set per tissue slice and incubated for 1 h at 37°C in a humidity chamber. Blocking buffer was tapped of, and the conjugated antibodies diluted in PLA probe diluent from the PLA probe kit were added for overnight incubation at 4°C and processed as described in the supplemental information.

Statistical analysis

The organoids were acquired from two or more differentiations, and we aimed to obtain 3 images per organoid. Each image was analyzed for retinal length, thickness, total cells, and total positive retinal cell population markers with ImageJ. Data were normalized per 100 μm of retinal length. For statistical analysis, GraphPad Prism v.8 was used. Shown values are expressed as mean ± standard error of the mean (SEM). Quantifications were tested for normality, and when distributed normally, unpaired t tests assuming equal variance were used to compare patient and CTRL lines. Measurements that did not show a normal distribution were tested with a Mann-Whitney test. Significance is indicated in graphs as $*p < 0.05$, $**p < 0.01$, and $***p < 0.001$.

SUPPLEMENTAL INFORMATION

Supplemental information can be found online at https://doi.org/10.1016/j.stemcr.2023.07.001.

AUTHOR CONTRIBUTIONS

Conceptualization, T.M.B., P.M.J.Q., L.P.P., I.B. and J.W.; methodology, retinal organoids and mouse morphology, T.M.B., P.M.J.Q., L.P.P., A.A.M., and D.K.; patient hiPSC line repair (isogenic controls) and characterization, C.H.A., C.F., H.M.M.M., N.B., and X.L.; formal analysis/data curation of the RNA-seq dataset, A.J., A.J.K., F.B., P.M.J.Q., and J.W.; formal analysis, retinal organoids and mouse morphology, T.M.B., P.M.J.Q., L.P.P., X.L., and N.B.; investigation, T.M.B., P.M.J.Q., L.P.P., X.L., N.B., and J.W.; writing – original draft, T.M.B. and J.W.; writing – review & editing, all authors;

visualization, T.M.B., P.M.J.Q., L.P.P., and J.W.; resources, I.B. and J.N.; supervision, J.W.; funding acquisition, T.M.B. and J.W.

ACKNOWLEDGMENTS

We thank Marco Heuvelman and Yacintha van Doorn for experimental work and the Wijnholds lab for advice on experiments and reviewing the manuscript. We thank Annelies Boonzaier-van der Laan and Lennard M. Voortman for technical assistance with microscopy and image analysis. Funding was provided by The Netherlands Organization for Health Research and Development (ZonMw grant 43200004 to J.W.), research grant MDBR-20-112-CRB1 from the University of Pennsylvania Orphan Disease Center in partnership with the Curing Retinal Blindness Foundation (to J.W.), research grant CRBF 2016-Aug-02 (to J.W.), LUMC Research Foundation Bontius Stichting 32072-8231, and the Dutch Blindness Funds (Uitzicht 2015-22 to J.W., Uitzicht 2020-01 to T.M.B. and J.W.): Rotterdamse Stichting Blindenbelangen, OogFonds, Stichting Blindenhulp, Stichting Retina Fonds, and Landelijke Stichting voor Blinden en Slechtzienden.

CONFLICT OF INTERESTS

The authors declare no competing interests. The LUMC is holder of patent number PCT/NL2014/050549, which describes the potential clinical use of CRB2; J.W. and L.P.P. are listed as co-inventor of this patent, and J.W. is an employee of the LUMC.

Received: December 22, 2021
Revised: July 3, 2023
Accepted: July 4, 2023
Published: August 3, 2023

REFERENCES

Aguilar-Aragon, M., Fletcher, G., and Thompson, B.J. (2020). The cytoskeletal motor proteins Dynein and MyoV direct apical transport of Crumbs. Dev. Biol. *459*, 126–137. https://doi.org/10.1016/j.ydbio.2019.12.009.

Alves, C.H., Sanz, A.S., Park, B., Pellissier, L.P., Tanimoto, N., Beck, S.C., Huber, G., Murtaza, M., Richard, F., Sridevi Gurubaran, I., et al. (2013a). Loss of CRB2 in the mouse retina mimics human retinitis pigmentosa due to mutations in the CRB1 gene. Hum. Mol. Genet. *22*, 35–50. https://doi.org/10.1093/hmg/dds398.

Alves, C.H., Bossers, K., Vos, R.M., Essing, A.H.W., Swagemakers, S., van der Spek, P.J., Verhaagen, J., and Wijnholds, J. (2013b). Microarray and morphological analysis of early postnatal CRB2 mutant retinas on a pure C57BL/6J genetic background. PLoS One *8*, e82532. https://doi.org/10.1371/journal.pone.0082532.

Boon, N., Lu, X., Andriessen, C.A., Moustakas, I., Buck, T.M., Freund, C., Arendzen, C.H., Böhringer, S., Mei, H., and Wijnholds, J. (2023). AAV-mediated gene augmentation therapy of CRB1 patient-derived retinal organoids restores the histological and transcriptional retinal phenotype. Stem Cell Rep. *18*, 1123–1137. https://doi.org/10.1016/j.stemcr.2023.03.014.

Boon, N., Wijnholds, J., and Pellissier, L.P. (2020). Research models and gene augmentation therapy for CRB1 retinal dystrophies. Front. Neurosci. *14*, 860. 00860. https://doi.org/10.3389/fnins.2020.

Buck, T.M., Vos, R.M., Alves, C.H., and Wijnholds, J. (2021). AAV-CRB2 protects against vision loss in an inducible CRB1 retinitis pigmentosa mouse model. Mol. Ther. Methods Clin. Dev. *20*, 423–441. https://doi.org/10.1016/j.omtm.2020.12.012.

Campa, C.C., Margaria, J.P., Derle, A., Del Giudice, M., De Santis, M.C., Gozzelino, L., Copperi, F., Bosia, C., and Hirsch, E. (2018). Rab11 activity and PtdIns(3)P turnover removes recycling cargo from endosomes. Nat. Chem. Biol. *14*, 801–810. https://doi.org/10.1038/s41589-018-0086-4.

Chen, X., and Emerson, M.M. (2021). Notch signaling represses cone photoreceptor formation through the regulation of retinal progenitor cell states. Sci. Rep. *11*, 14525. https://doi.org/10.1038/s41598-021-93692-w.

Clark, B.S., Miesfeld, J.B., Flinn, M.A., Collery, R.F., and Link, B.A. (2020). Dynamic polarization of Rab11a modulates Crb2a localization and impacts signaling to regulate retinal neurogenesis. Front. Cell Dev. Biol. *8*, 608112. https://doi.org/10.3389/fcell.2020.608112.

Farfel-Becker, T., Roney, J.C., Cheng, X.T., Li, S., Cuddy, S.R., and Sheng, Z.H. (2019). Neuronal soma-derived degradative lysosomes are continuously delivered to distal axons to maintain local degradation capacity. Cell Rep. *28*, 51–64.e4. https://doi.org/10.1016/j.celrep.2019.06.013.

Gajovic, S., Mitrecic, D., Augustincic, L., Iaconcig, A., and Muro, A.F. (2006). Unexpected rescue of alpha-synuclein and multimerin1 deletion in C57BL/6JOlaHsd mice by beta-adducin knockout. Transgenic Res. *15*, 255–259. https://doi.org/10.1007/s11248-006-0003-6.

Gaullier, J.M., Simonsen, A., D'Arrigo, A., Bremnes, B., Stenmark, H., and Aasland, R. (1998). FYVE fingers bind PtdIns(3)P. Nature *394*, 432–433. https://doi.org/10.1038/28767.

Ha, T., Moon, K.H., Dai, L., Hatakeyama, J., Yoon, K., Park, H.S., Kong, Y.Y., Shimamura, K., and Kim, J.W. (2017). The retinal pigment epithelium is a notch signaling niche in the mouse retina. Cell Rep. *19*, 351–363. https://doi.org/10.1016/j.celrep.2017.03.040.

Herranz, H., Stamataki, E., Feiguin, F., and Milán, M. (2006). Self-refinement of Notch activity through the transmembrane protein Crumbs: modulation of gamma-secretase activity. EMBO Rep. *7*, 297–302. https://doi.org/10.1038/sj.embor.7400617.

Kakar-Bhanot, R., Brahmbhatt, K., Chauhan, B., Katkam, R.R., Bashir, T., Gawde, H., Mayadeo, N., Chaudhari, U.K., and Sachdeva, G. (2019). Rab11a drives adhesion molecules to the surface of endometrial epithelial cells. Hum. Reprod. *34*, 519–529. https://doi.org/10.1093/humrep/dey365.

Kraut, R.S., and Knust, E. (2019). Changes in endolysosomal organization define a pre-degenerative state in the crumbs mutant Drosophila retina. PLoS One *14*, e0220220. https://doi.org/10.1371/journal.pone.0220220.

Laprise, P. (2011). Emerging role for epithelial polarity proteins of the Crumbs family as potential tumor suppressors. J. Biomed. Biotechnol. *2011*, 868217. https://doi.org/10.1155/2011/868217.

Lattner, J., Leng, W., Knust, E., Brankatschk, M., and Flores-Benitez, D. (2019). Crumbs organizes the transport machinery by regulating apical levels of PI(4,5)P(2) in Drosophila. Elife *8*, e50900. https://doi.org/10.7554/eLife.50900.

Nemetschke, L., and Knust, E. (2016). Drosophila Crumbs prevents ectopic Notch activation in developing wings by inhibiting ligand-independent endocytosis. Development *143*, 4543–4553. https://doi.org/10.1242/dev.141762.

Nguyen, X.T.A., Talib, M., van Schooneveld, M.J., Wijnholds, J., van Genderen, M.M., Schalij-Delfos, N.E., Klaver, C.C.W., Talsma, H.E., Fiocco, M., Florijn, R.J., et al. (2022). CRB1-associated retinal dystrophies: a prospective natural history study in anticipation of future clinical trials. Am. J. Ophthalmol. *234*, 37–48. https://doi.org/10.1016/j.ajo.2021.07.021.

Ohata, S., Aoki, R., Kinoshita, S., Yamaguchi, M., Tsuruoka-Kinoshita, S., Tanaka, H., Wada, H., Watabe, S., Tsuboi, T., Masai, I., and Okamoto, H. (2011). Dual roles of Notch in regulation of apically restricted mitosis and apicobasal polarity of neuroepithelial cells. Neuron *69*, 215–230. https://doi.org/10.1016/j.neuron.2010.12.026.

Overhoff, M., De Bruyckere, E., and Kononenko, N.L. (2021). Mechanisms of neuronal survival safeguarded by endocytosis and autophagy. J. Neurochem. *157*, 263–296. https://doi.org/10.1111/jnc.15194.

Pellissier, L.P., Alves, C.H., Quinn, P.M., Vos, R.M., Tanimoto, N., Lundvig, D.M.S., Dudok, J.J., Hooibrink, B., Richard, F., Beck, S.C., et al. (2013). Targeted ablation of CRB1 and CRB2 in retinal progenitor cells mimics Leber congenital amaurosis. PLoS Genet. *9*, e1003976. https://doi.org/10.1371/journal.pgen.1003976.

Pellissier, L.P., Lundvig, D.M.S., Tanimoto, N., Klooster, J., Vos, R.M., Richard, F., Sothilingam, V., Garcia Garrido, M., Le Bivic, A., Seeliger, M.W., and Wijnholds, J. (2014). CRB2 acts as a modifying factor of CRB1-related retinal dystrophies in mice. Hum. Mol. Genet. *23*, 3759–3771. https://doi.org/10.1093/hmg/ddu089.

Pellissier, L.P., Quinn, P.M., Alves, C.H., Vos, R.M., Klooster, J., Flannery, J.G., Heimel, J.A., and Wijnholds, J. (2015). Gene therapy into photoreceptors and Müller glial cells restores retinal structure and function in CRB1 retinitis pigmentosa mouse models. Hum. Mol. Genet. *24*, 3104–3118. https://doi.org/10.1093/hmg/ddv062.

Pocha, S.M., Wassmer, T., Niehage, C., Hoflack, B., and Knust, E. (2011). Retromer controls epithelial cell polarity by trafficking the apical determinant Crumbs. Curr. Biol. *21*, 1111–1117. https://doi.org/10.1016/j.cub.2011.05.007.

Pradhan, J., Noakes, P.G., and Bellingham, M.C. (2019). The Role of altered BDNF/TrkB signaling in amyotrophic lateral sclerosis. Front. Cell. Neurosci. *13*, 368. https://doi.org/10.3389/fncel.2019.00368.

Quinn, P.M., Alves, C.H., Klooster, J., and Wijnholds, J. (2018). CRB2 in immature photoreceptors determines the superior-inferior symmetry of the developing retina to maintain retinal structure and function. Hum. Mol. Genet. *27*, 3137–3153. https://doi.org/10.1093/hmg/ddy194.

Quinn, P.M., Buck, T.M., Mulder, A.A., Ohonin, C., Alves, C.H., Vos, R.M., Bialecka, M., van Herwaarden, T., van Dijk, E.H.C., Talib, M., et al. (2019a). Human iPSC-derived retinas recapitulate the fetal CRB1 CRB2 complex formation and demonstrate that photoreceptors and muller glia are targets of AAV5. Stem Cell Rep. *12*, 906–919. https://doi.org/10.1016/j.stemcr.2019.03.002.

Quinn, P.M., Mulder, A.A., Henrique Alves, C., Desrosiers, M., de Vries, S.I., Klooster, J., Dalkara, D., Koster, A.J., Jost, C.R., and Wijnholds, J. (2019b). Loss of CRB2 in Muller glial cells modifies a CRB1-associated retinitis pigmentosa phenotype into a Leber congenital amaurosis phenotype. Hum. Mol. Genet. *28*, 105–123. https://doi.org/10.1093/hmg/ddy337.

Richardson, E.C.N., and Pichaud, F. (2010). Crumbs is required to achieve proper organ size control during Drosophila head development. Development *137*, 641–650. https://doi.org/10.1242/dev.041913.

Ridley, S.H., Ktistakis, N., Davidson, K., Anderson, K.E., Manifava, M., Ellson, C.D., Lipp, P., Bootman, M., Coadwell, J., Nazarian, A., et al. (2001). FENS-1 and DFCP1 are FYVE domain-containing proteins with distinct functions in the endosomal and Golgi compartments. J. Cell Sci. *114*, 3991–4000. https://doi.org/10.1242/jcs.114.22.3991.

Rosa-Ferreira, C., and Munro, S. (2011). Arl8 and SKIP act together to link lysosomes to kinesin-1. Dev. Cell *21*, 1171–1178. https://doi.org/10.1016/j.devcel.2011.10.007.

Teranishi, H., Tabata, K., Saeki, M., Umemoto, T., Hatta, T., Otomo, T., Yamamoto, K., Natsume, T., Yoshimori, T., and Hamasaki, M. (2022). Identification of CUL4A-DDB1-WDFY1 as an E3 ubiquitin ligase complex involved in initiation of lysophagy. Cell Rep. *40*, 111349. https://doi.org/10.1016/j.celrep.2022.111349.

Thompson, B.J., Pichaud, F., and Röper, K. (2013). Sticking together the Crumbs - an unexpected function for an old friend. Nat. Rev. Mol. Cell Biol. *14*, 307–314. https://doi.org/10.1038/nrm3568.

Trousdale, C., and Kim, K. (2015). Retromer: structure, function, and roles in mammalian disease. Eur. J. Cell Biol. *94*, 513–521. https://doi.org/10.1016/j.ejcb.2015.07.002.

van de Pavert, S.A., Kantardzhieva, A., Malysheva, A., Meuleman, J., Versteeg, I., Levelt, C., Klooster, J., Geiger, S., Seeliger, M.W., Rashbass, P., et al. (2004). Crumbs homologue 1 is required for maintenance of photoreceptor cell polarization and adhesion during light exposure. J. Cell Sci. *117*, 4169–4177. https://doi.org/10.1242/jcs.01301.

van de Pavert, S.A., Sanz, A.S., Aartsen, W.M., Vos, R.M., Versteeg, I., Beck, S.C., Klooster, J., Seeliger, M.W., and Wijnholds, J. (2007). Crb1 is a determinant of retinal apical Muller glia cell features. Glia *55*, 1486–1497. https://doi.org/10.1002/glia.20561.

van Rossum, A.G., Aartsen, W.M., Meuleman, J., Klooster, J., Malysheva, A., Versteeg, I., Arsanto, J.P., Le Bivic, A., and Wijnholds, J. (2006). Pals1/Mpp5 is required for correct localization of Crb1 at the subapical region in polarized Mueller glia cells. Hum. Mol. Genet. *15*, 2659–2672. https://doi.org/10.1093/hmg/ddl194.

Wang, S., Tan, K.L., Agosto, M.A., Xiong, B., Yamamoto, S., Sandoval, H., Jaiswal, M., Bayat, V., Zhang, K., Charng, W.L., et al.

(2014). The retromer complex is required for rhodopsin recycling and its loss leads to photoreceptor degeneration. PLoS Biol. *12*, e1001847. https://doi.org/10.1371/journal.pbio.1001847.

Wooten, M.W., Geetha, T., Babu, J.R., Seibenhener, M.L., Peng, J., Cox, N., Diaz-Meco, M.T., and Moscat, J. (2008). Essential role of sequestosome 1/p62 in regulating accumulation of Lys63-ubiquitinated proteins. J. Biol. Chem. *283*, 6783–6789. https://doi.org/10.1074/jbc.M709496200.

Zhou, B., Wu, Y., and Lin, X. (2011). Retromer regulates apical-basal polarity through recycling Crumbs. Dev. Biol. *360*, 87–95. https://doi.org/10.1016/j.ydbio.2011.09.009.

Zou, J., Wang, X., and Wei, X. (2012). Crb apical polarity proteins maintain zebrafish retinal cone mosaics via intercellular binding of their extracellular domains. Dev. Cell *22*, 1261–1274. https://doi.org/10.1016/j.devcel.2012.03.007.

Stem Cell Reports, Volume *18*

Supplemental Information

CRB1 is required for recycling by RAB11A+ vesicles in human retinal organoids

Thilo M. Buck, Peter M.J. Quinn, Lucie P. Pellissier, Aat A. Mulder, Aldo Jongejan, Xuefei Lu, Nanda Boon, Daniëlle Koot, Hind Almushattat, Christiaan H. Arendzen, Rogier M. Vos, Edward J. Bradley, Christian Freund, Harald M.M. Mikkers, Camiel J.F. Boon, Perry D. Moerland, Frank Baas, Abraham J. Koster, Jacques Neefjes, Ilana Berlin, Carolina R. Jost, and Jan Wijnholds

Supplementary data related to manuscript:
CRB1 is required for recycling by RAB11A+ vesicles in human retinal organoids.
Buck TM *et al.*, 2023

Overview

Supplemental Figures and Legends

Figure S1. The retinal morphological phenotype is milder in *Crb1*^{KO}*Crb2*$^{\Delta RPC}$ on 100% C57/B6 genetic background than on 50% mixed genetic background. Related to Figure 1.
Figure S2. The *Crb1*^{KO}*Crb2*$^{\Delta RPC}$ mouse displays an LCA-like *CRB1* phenotype. Related to Figure 1.
Figure S3. Validation of the isogenic control iPS cell lines. Related to Figure 3-7.
Figure S4. The onset of CRB1 expression in control organoids. Related to Figure 4.
Figure S5. More lysosomes are present in *CRB1* patient retinal organoids. Related to Figure 4-5.
Figure S6. Increased number of degradative vesicles/compartments in *CRB1* patient retinal organoids. Related to Figure 5.
Figure S7. Dysregulation of the endolysosomal system in *CRB1* patient retinal organoids. Related to Figure 6.
Table S1. Information on hiPSC lines. Related to Figure 2-6.
Table S2: Antibody list and dilution used for immunohistochemistry and western blots. Related to all figures.
Table S3. ISO-02-P116 top 10 off-target candidates. Related to figures 3-7.
Table S4. ISO-03-P116 top 10 off-target candidates. Related to figures 3-7.
Table S5. ISO-02 P128 top 10 off-target candidates. Related to figures 3-7.
Table S6. Karyotyping of hiPS cell lines. Related to figures 2-7.

Supplemental Experimental Procedures

Supplemental Information

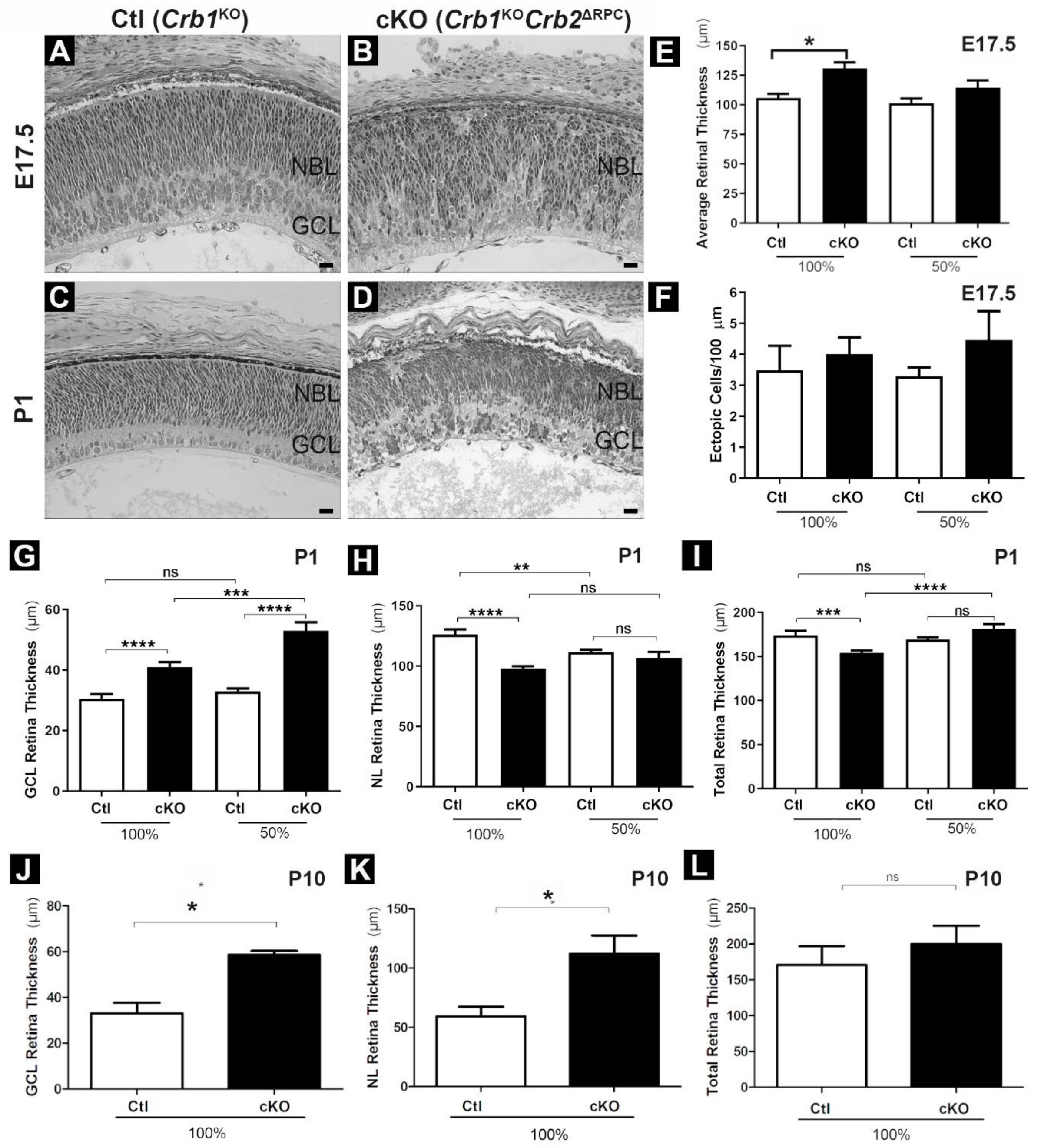

Figure S1. The retinal morphological phenotype is milder in *Crb1*^{KO}*Crb2*^{ΔRPC} on 100% C57/B6 genetic background than on 50% mixed genetic background. (A-D) Retinal morphology on plastic sections of Ctl (*Crb1*^{KO}) and cKO (*Crb1*^{KO}*Crb2*^{ΔRPC}) mice on 100% C57/B6 genetic background at E17.5 and P1. (E-F) Ctl (*Crb1*^{KO}) and cKO (*Crb1*^{KO}*Crb2*^{ΔRPC}) mice on 50% and 100% C57/B6 genetic background. (E-F) Average retinal thickness and ectopic nuclei per 100 µm retinal length at E17.5. (G-I) Ganglion cell layer (GCL) thickness, neuroretina layer (NL) thickness, and retinal thickness (OLM-ILM) at P1. (J-L) GCL thickness, NL thickness, and retinal thickness in 100% C57/B6 genetic background at P10. Scale bar, 20 µm. $p<0.05$ (*), $p<0.01$ (**), and $p<0.001$ (***). Related to Figure 1.

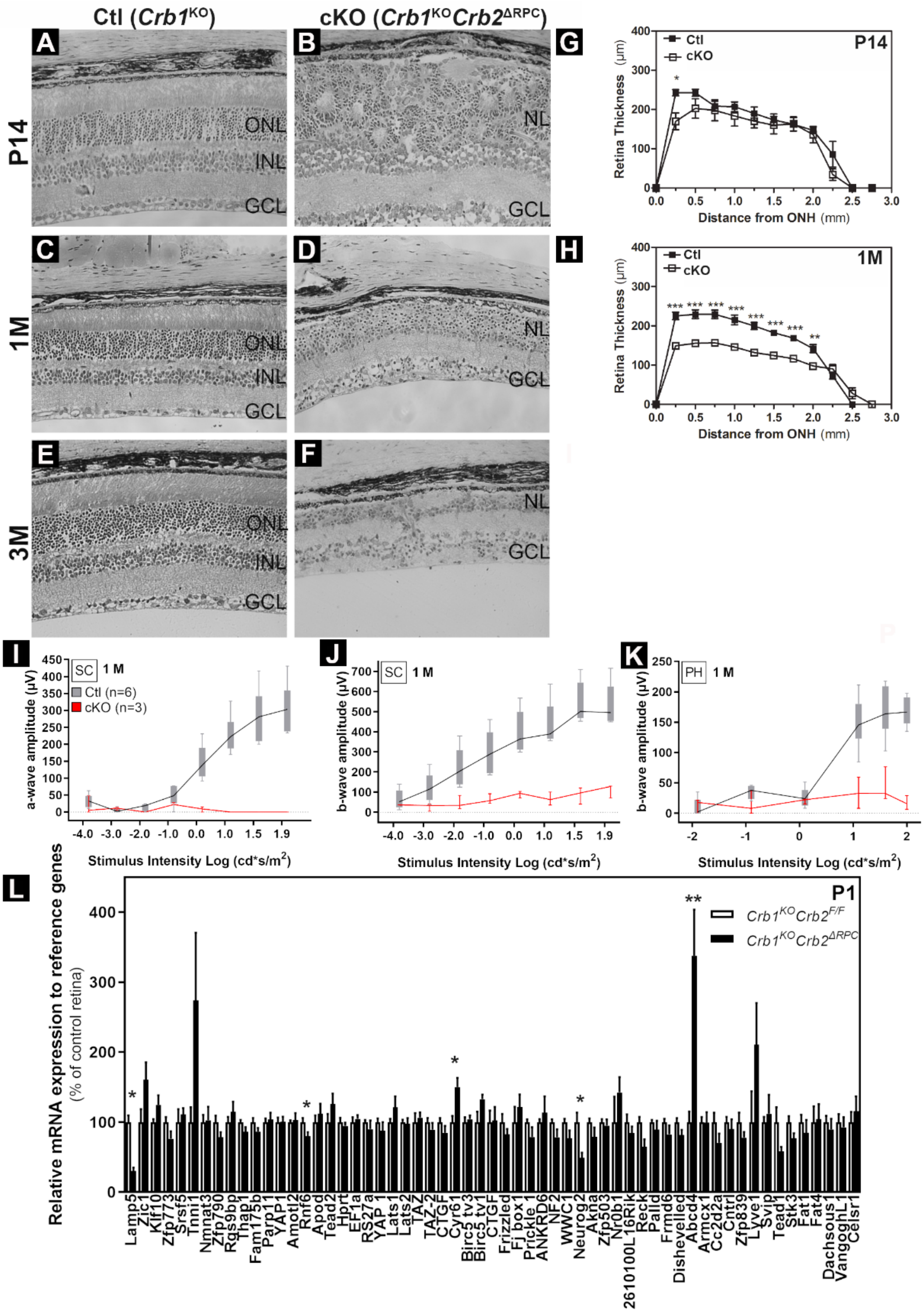

Figure S2. The $Crb1^{KO}Crb2^{\Delta RPC}$ mouse displays an LCA-like *CRB1* phenotype. (A-F) Retinal morphology on plastic sections of Ctl ($Crb1^{KO}$) and cKO ($Crb1^{KO}Crb2^{\Delta RPC}$) mice on 100% C57/B6 genetic background at P14, 1M, and 3M. (G-H) Retinal thickness (OLM-ILM) at P14, 1M, and 3M. (I-K) Electroretinographic (ERG) analysis of the retinal function at 1M. Conditional KOs are in red and Ctl age-matched littermates in gray: (L-M) Scotopic (SC) single-flash intensity series (-4, -3 , -2, -1, 0, 1, 1.5, 1.9 log cd s/m² light intensity). (I) scotopic a-wave amplitudes. (J) scotopic b-wave amplitudes. (K) Photopic (PH) single-flash ERG at different light intensities (-2, -1, 0, 1, 1.5, 1.9 log cd s/m² light intensity at 30 cd/m² background light). Boxes indicate the 25 and 75% quantile range, whiskers indicate the 5 and 95% quantiles, and the intersection of line and error bar indicates the median of the data. (L) $Crb1^{KO}Crb2^{\Delta RPC}$ vs $Crb1^{KO}$ retina qPCR validation (n=5 retinas / group) of transcript changes at P1. Statistical significance calculated by a two-sided T-Test ($p<0.05$; 0.01; 0.001 shown as *, **, and ***). Scale bar, 20 µm. Related to Figure 1.

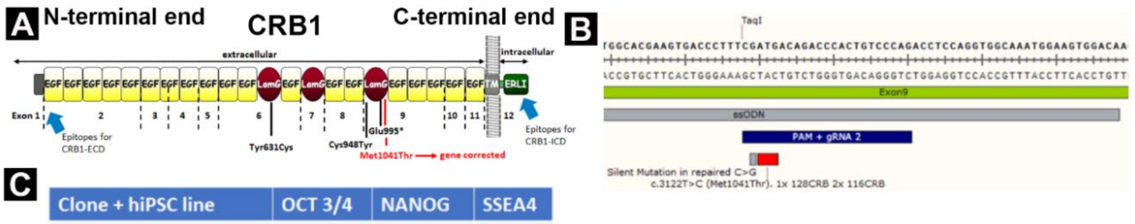

C

Clone + hiPSC line	OCT 3/4	NANOG	SSEA4
iso02LUMC0116iCRB09	90.5%	93.4%	89.9%
iso03LUMC0116iCRB09	88.0%	91.6%	95.5%
iso04LUMC0116iCRB09	99.5%	97.3%	86.9%
iso01LUMC0128iCRB01	96.5%	94.0%	99.2%
iso02LUMC0128iCRB01	82.3%	83.5%	85.8%
iso05LUMC0128iCRB01	98.7%	97.6%	99.3%

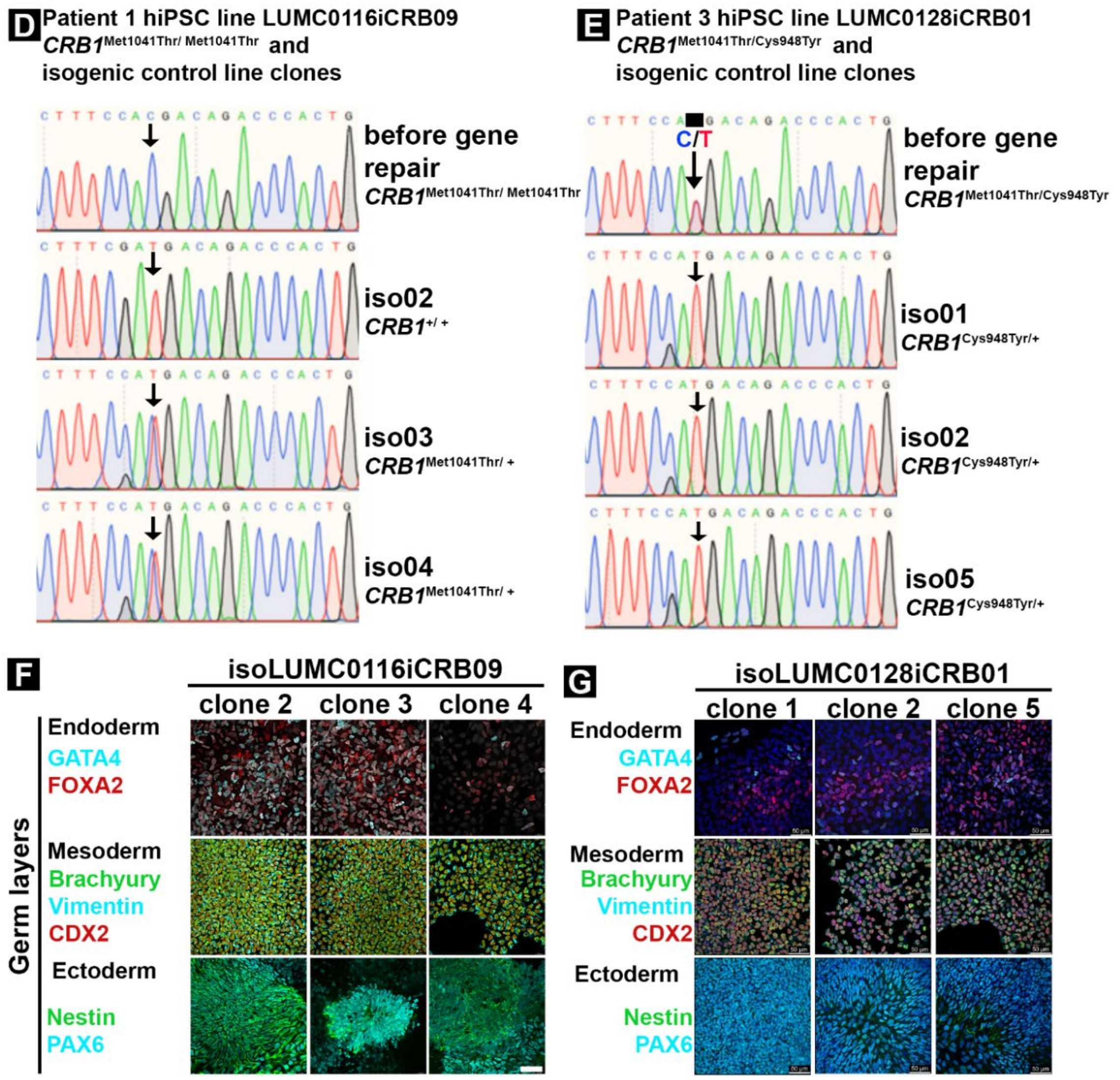

Figure S3. Validation of the isogenic control iPS cell lines. (A) CRB1 protein domains and the corresponding exons, CRB1 patient variants, and the epitope of the CRB1-ICD and CRB1-ECD antibodies. (B) Gene correction by single-stranded oligodeoxynucleotides (ssODN), gRNA, and nuclease Cas9 on locus c.3122T>C. (C) Flow cytometry sorted human iPSC clones indicating expression of pluripotency markers SSEA4, OCT3/4, and NANOG. (D-E) Sanger sequence validation of 3 clones per patient line. (F-G) human iPSC clones can be differentiated in to the three germ layers. Scale bar, 50 µm. Related to Figure 3-7.

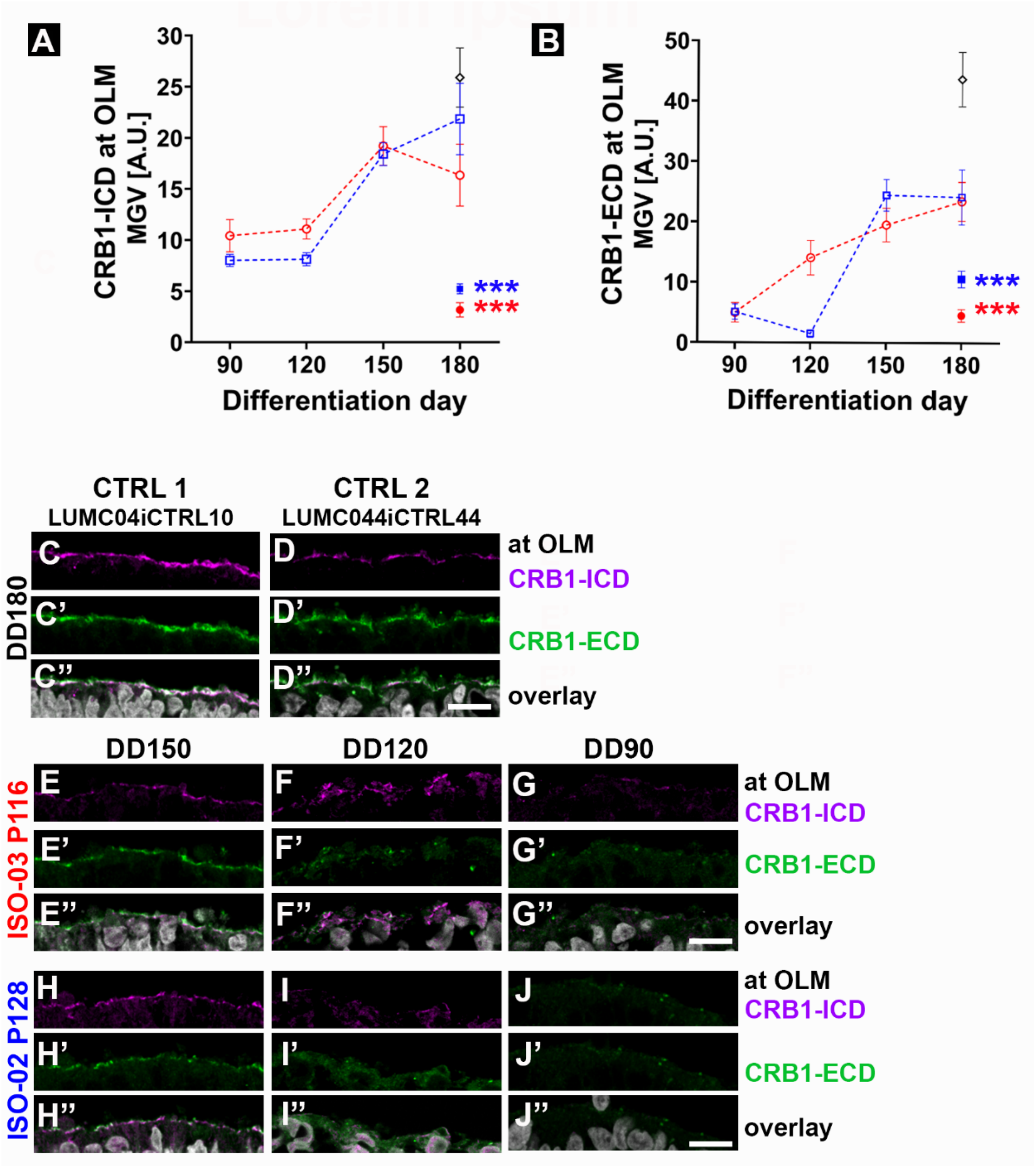

Figure S4. The onset of CRB1 expression in control organoids. (A-B) CRB1-Alexa conjugated fluorescence signal detected over time on ICD-(A) and ECD-(B) CRB1 antibodies (empty circles/dashed lines, isogenic controls; filled circles, patient lines; black circle, CTRL's; DD180, n=8; DD90-150, n=4-6 organoids per line). (C-G) CRB1 stained with an (C-J) intracellular (ICD) or (C'-G') extracellular domain (ECD) epitope antibodies at the OLM, and (C''-G'') overlay (scale bar, 10 µm) at DD180 (C-D; CTRL 1+2), DD150 (E+H; isogenic ISO-03 P116 and ISO-P128), DD120 (F+I; isogenic ISO-03 P116 and ISO-P128), and DD90 (G+J; isogenic ISO-03 P116 and ISO-P128). OLM, outer limiting membrane; ICD, intracellular domain; ECD, extracellular domain; DD, differentiation day. Each datapoint in the graphs A and B represents individual organoids, of which an average has been taken of 3 representative images. The standard error of mean (SEM) is derived from these averages. The number of individual organoids per time point (DD90; DD120; DD150; DD180) for condition ISO-03 P116 is n=6; 5; 5; 8; for ISO-02 P128 is n= 6; 6; 6; 6; for P116 (DD180) n=8; for P128 (DD180) n=8; and for CTRL1 (DD180) n=8. From at least two independent organoid batches. Statistical analysis: $p < 0.001$(***). Related to Figure 4.

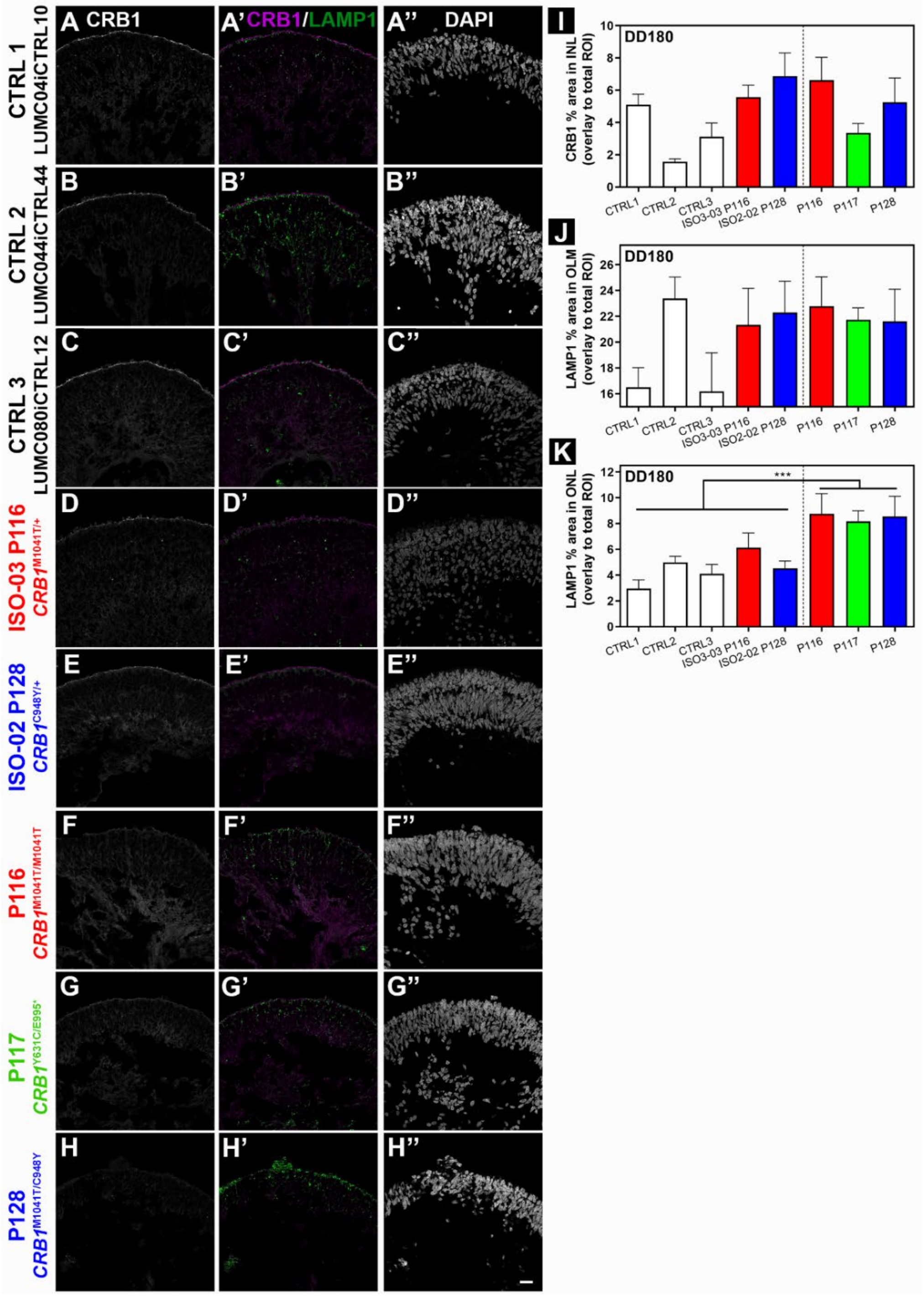

Figure S5. More lysosomes are present in CRB1 patient retinal organoids. Immunofluorescence CRB1 puncta (A-H; grayscale), co-labelling CRB1/LAMP1 (green/magenta in A'-H'), and nuclei (grayscale in A''-H'') in *CRB1* patient retinal organoids at DD180. (A-H) patient CRB1 retinal organoids express little CRB1 protein throughout the retina. (A'-H') Little co-labelling of CRB1 (magenta) and lysosomes (LAMP1+, green) were found. (A'-H') Patient CRB1 retinal organoids have more lysosomes at the ONL. (A''-H'') Nuclei staining (DAPI, grayscale) showing overall morphology. (I) CRB1-fluorescence signal measured by puncta in the INL. (J-K) LAMP1+ puncta measured in the OLM and ONL. Scale bar, 50 µm. Each datapoint in the graph represents individual organoids, of which an average has been taken of 3 representative images. The standard error of mean (SEM) is derived from these averages. The number of individual organoids per condition (CTRL1; CTRL2; CTRL3; ISO-03 P116; ISO-02 P128; P116; P117; P128) in (I-K) is n=9; 9; 9; 10; 10; 12; 9; 13. From at least two independent organoid batches. Statistical analysis: $p < 0.001$(***). Related to Figure 4-5.

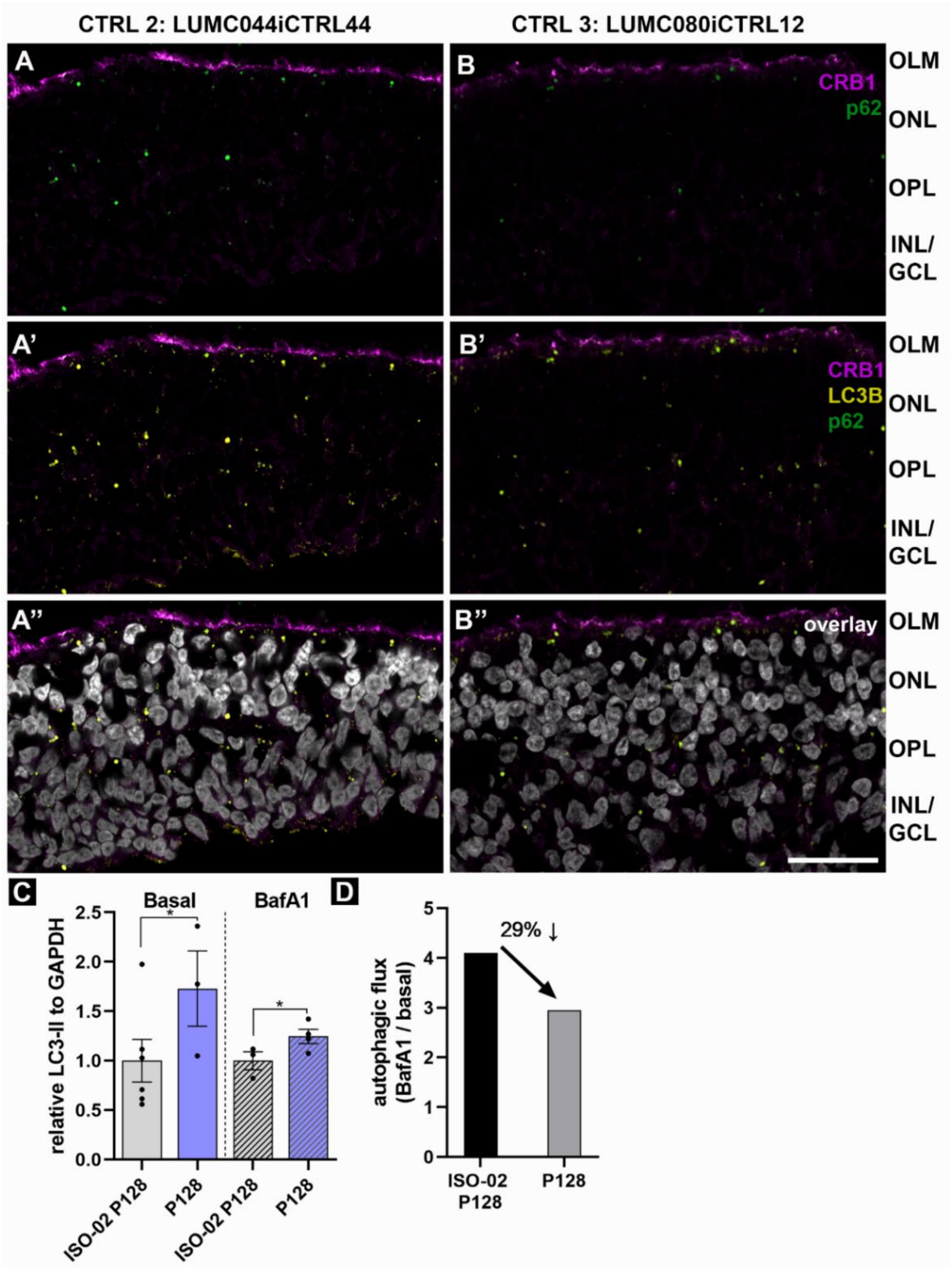

Figure S6. Increased number of degradative vesicles/compartments in *CRB1* patient retinal organoids. (A-B) Immunofluorescence triple staining CRB1 (magenta), p62 (green), and LC3B (yellow). (A-B) LC3B and p62 localized overall more in OLM layer, OPL layer and INL layer in control retinal organoids. Scale bar, 50 µm. (C-D) westernblot related to Figure 5 and Figure S6. (C) Analysis of the protein band intensities of the western blot shown in Figure 5I of individual lysed organoids (depicted as dots) stained for LC3B (LC3-I and LC3-II; 19/17 kD), recoverin for photoreceptors (26 kD), and GAPDH (housekeeping control, 37 kD) but organoids lacking recoverin expression were removed from the analysis. (D) Western blot protein band intensities of the BafA1 condition divided by the basal condition showing a decrease in the autophagic flux in *CRB1* patient retinal organoids. Each datapoint in the graphs J and K represents individual organoids, of which an average has been taken. The standard error of mean (SEM) is derived from these averages. Number of individual organoids per condition and differentiation round is n=3-6 organoids per line from two independent organoid batches. Statistical analysis: $p<0.05$ (*), and $p<0.001$ (***). Related to Figure 5.

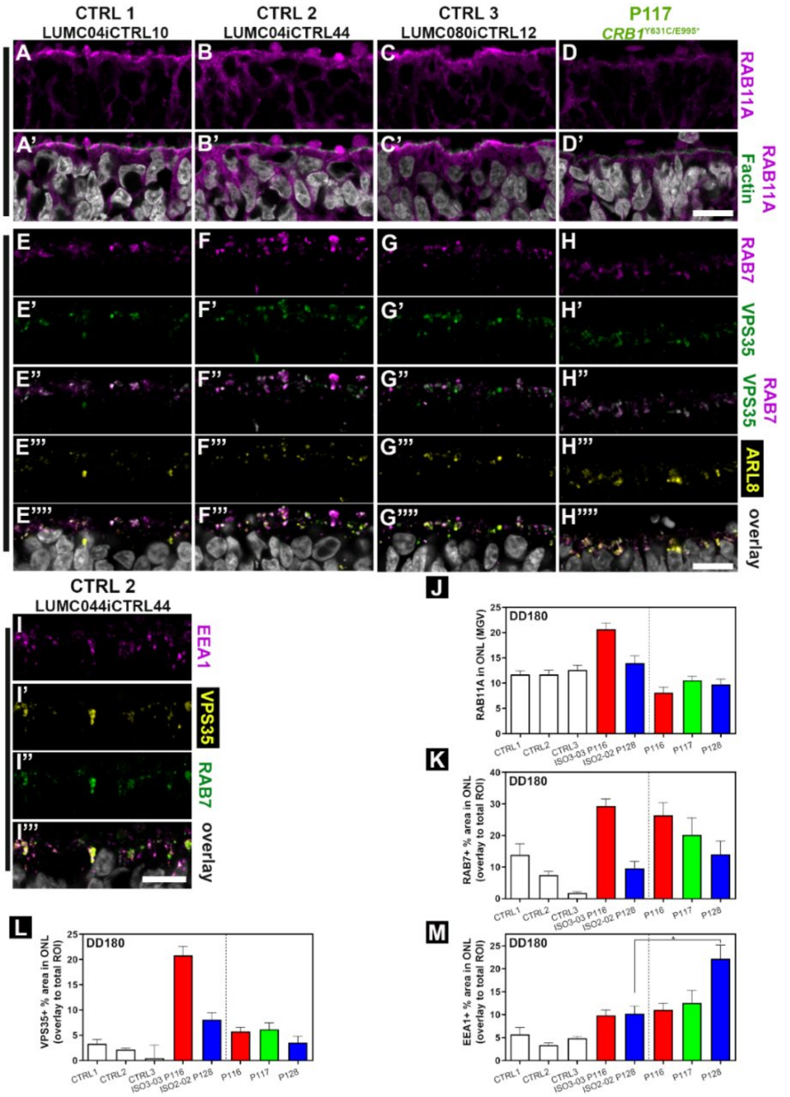

Figure S7. Dysregulation of the endolysosomal system in patient *CRB1* patient retinal organoids. (A+E) CTRL 1 (LUMC04CTRL10) retinal organoids, (B+F+I) CTRL 2 (LUMC044iCTRL44) retinal organoids, (C+G) CTRL3 (LUMC080iCTRL12) retinal organoids, and (D+H) patient 2 (LUMC0117iCRB01) retinal organoids. (A-D) Immunofluorescence labelling of RAB11A (magenta), and phalloidin (F-actin; green). (E-H) Immunofluorescence labeling of RAB7 (magenta), VPS35 (green), and ARL8A/B (yellow). (E-H) Immunofluorescence labeling of EEA1 (magenta), RAB7 (green), and ARL8A/B (yellow). (I) Immunofluorescence labeling of EEA1 (magenta), RAB7 (green), and VPS35 (yellow). Overlay shown in white (E-I). (J-M) Semi-quantification of fluorescence signal. (J) RAB11A mean gray value (MVG) in ONL. (K) Total RAB7 particle area / total ONL area in %. (L) RAB11A mean gray value (MVG) in ONL. (K) Total VPS35 particle area / total ONL area in %. (M) RAB11A mean gray value (MVG) in ONL. (K) Total EEA1 particle area / total ONL area in %. All retinal organoids at DD180 collected. Scale bar, 10 µm. Each datapoint in the graph represents individual organoids, of which an average has been taken of 3 representative images. The standard error of mean (SEM) is derived from these averages. Number of individual organoids per condition and differentiation round is n=6-14 organoids per line from two independent organoid batches; Statistical analysis: *p < 0.05. Related to Figure 6.

Table S1. Information on human iPSC lines. Related to Figure S3 and Figure 2-6.

Line code	Description	Gender
Control-derived and gene corrected (isogenic, iso) human iPSC line names		
LUMC0004iCTRL10	Control line 1 (CTRL 1)	male
LUMC0044iCTRL44	Control line 2 (CTRL 2)	female
LUMC0080iCTRL12	Control line 3 (CTRL 3)	male
iso01LUMC0116iCRB09	isogenic: Allele 1: c.3122T>C gene corrected to c.3120C>G. Allele 2: c.3122T>C. p.(Met1041Thr)	male
iso02LUMC0116iCRB09	isogenic: Allele 1: c.3122T>C gene corrected to c.3120C>G. Allele 2: c.3122T>C. p.(Met1041Thr)	male
iso03LUMC0116iCRB09	isogenic: Allele 1 and 2: Homozygous c.3122T>C gene corrected to c.3120C>G	male
iso01LUMC0128iCRB01	isogenic: Allele 1: c.2843G>A --> p.(Cys948Tyr). Allele 2: c.3122T>C gene corrected to c.3120C>G	male
iso02LUMC0128iCRB01	Isogenic: Allele 1: c.2843G>A. p.(Cys948Tyr). Allele 2: c.3122T>C gene corrected to c.3120C>G	male
iso03LUMC0128iCRB01	isogenic: Allele 1: c.2843G>A. p.(Cys948Tyr). Allele 2: c.3122T>C gene corrected to c.3120C>G	male
iso05LUMC0128iCRB01	isogenic: Allele 1: c.2843G>A. p.(Cys948Tyr). Allele 2: c.3122T>C gene corrected to c.3120C>G	male
Patient *CRB1* Retinitis pigmentosa-derived human iPSC lines		
LUMC0116iCRB09	Patient 1. Allele 1 and 2: homozygous c.3122T>C--> p.(Met1041Thr)	male
LUMC0117iCRB01	Patient 2. Allele 1: c.1892A>G (p.Tyr631Cys)). Allele 2: c.2911G>T (p.(Glu995*))	male
LUMC0128iCRB01	Patient 3. Allele 1: c.2843G>A --> p.(Cys948Tyr). Allele 2: c.3122T>C --> p.(Met1041Thr)	male

Table S2: Antibody list and dilution used for immunohistochemistry and western blots. Related to all figures.

Host; antibody	Dilution + procedure	Catalogue number
**Primary antibodies**		
Mouse anti-CRB1 (extracellular)	1/200 IF, 1/300 PLA	H00023418-A01
Rabbit anti-CRB1 (AK2; intracellular)	1/100 IF	Home-made
Phalloidin-TRITC (*aka* F-actin)	1/250 IF	r-415
Rabbit anti-OTX2	1/200 IF	13497-1-AP
Mouse anti-BRN3A	1/100 IF	sc-8429
Mouse anti-LC3B	1/100 IF + WB 1/300	0231-100/LC-3-5F10
Rabbit anti-FENS1 (*aka* WDFY1)	1/200 IF	AB125329
Mouse anti-EEA1	1/100 IF	BD-610457
Mouse anti-LAMP1 (aka CD107a)	1/100 IF	sc-20011
Rabbit anti-Cathepsin D (ab-2 clone)	1/200 IF	IM16-100UG
Mouse anti-ARL8A/B	1/100 IF	sc-398635
Mouse anti-NOTCH1 (extracellular)	1/300 IF + PLA	MA5-11961
Mouse anti-PIP2 (*aka* PtdIns(4,5)P_2)	1/200 IF	MA3-500
Rabbit anti-(pan-)cytokeratin	1/200 IF	AB9377
Rabbit anti-RCVRN	1/600 IF, 1/5000 WB	AB5585
Mouse anti-GAPDH	1/5000 WB	MAB374
Rabbit anti-RAB7	1/100 IF	CST9367S
Goat anti-VPS35 (retromer)	1/100 IF	Ab10099
Rabbit anti-VPS26 (retromer)	1/250 IF	Ab181352
Rabbit anti-RAB11a	1/100 IF	71-5300
Rat anti-CD44	1/100 IF	553132
Mouse anti-Oct3/4-BV421	1/25 FACS	565644
Mouse anti-NANOG-PE	1/5 FACS	560483
anti-SSEA4-FITC	1/25 FACS	130-098-371
Mouse anti-Nestin-Alexa488 CC	1/200 IF	CST; clone 10C2
Rabbit PAX6-Alexa647 c CC	1/200 IF	CST; clone D3A9V
Rabbit FOXA2-Alexa555 CC	1/500 IF	CST; clone D56D6
Rabbit GATA4-Alexa647 CC	1/200 IF	CST; clone D3A3M
Rabbit Vimentin-Alexa647 CC	1/400 IF	CST; clone D21H3
Rabbit CDX2-Alexa555 CC	1/500 IF	CST; clone D11D10
Rabbit Brachyury-Alexa488 CC	1/200 IF	CST; clone D2Z3J
**Secondary antibodies**		
Anti-rabbit-IgG-HRP	1/5000 WB	sc-2357
Anti-mouse-IgGκ BP-HRP	1/5000 WB	sc-516102
Anti-rabbit Alexa647	1/1000 IF	Ab150083
Anti-mouse Alexa647	1/1000 IF	Ab150119
Anti-mouse Alexa488	1/1000 IF	Ab150113
Anti-chicken Alexa555	1/1000 IF	Ab150169
Anti-rabbit Alexa555	1/1000 IF	Ab150086
Anti-rat cy3 (50% glycerol added)	1/500 IF	712-165-153
Anti-rabbit cy3 (50% glycerol added)	1/500 IF	111-165-045

*IF, immunofluorescence; WB, western blot; PLA, Proximity ligation assay; CC, custom made conjugated

**Table S3: ISO-02-P116 top 10 off-target candidates. Related to figures 3-7.**			
**Gene Locus**	FW primer sequence (5’ to 3’)<br>RV primer sequence (5’ to 3’)	Target Sequence (5’ to 3’)	Off target mutation
Intron:RP11-132N15.3<br>**chr3:187689636-187689658:+**	FW 5’- TTGGGGCATACAAAGGAGGG - 3’<br>RV 5’- TTTCTGAATAGGCTGCCCGT - 3’	AGCCCGTGTT**CTGGAACAGAGAATCTGTCA**GGGGTGTG	None
Intron:RP1-91J24.3<br>**chr6:144636458-144636480:+**	FW 5’ - ACATAAGCCAGCCTGCAGAA - 3’<br>RV 5’ - GGCCCTGTCCAACTAGTGTC - 3’	GGATGGCTGG**CAGGGGCAGTAGGTCAGTCA**GGGCACTG	None
Intergenic: NARS-RP11-35G9.3<br>**chr18:55296831-55296853:-**	FW 5’- TCCTAAGGACGCCCTACTCT - 3’<br>RV 5’ – TCCCAAACTCAGAGGCGTAAC - 3’	CTCAGCTTCC**CTGGACCAGTGGGTCAGTCA**GGGCGTGT	None
Intergenic: HNRNPA 1P57-AC010739.1<br>**chr2:41,415,579-41,415,601:-**	FW 5’- TGGAGCCAAACTCTCTGGGTT - 3’<br>RV 5’- AATTAGGTTCCTTCCTGGCCT - 3’	TGCCATATTC**CTAGGACACTGGATCAGTCA**AGGCCTAG	None
Intron: LINC01088<br>**chr4:79946400-79946422:+**	FW 5’- CACAGCAGCAACAGTCATGG - 3’<br>RV 5’- TTGTGGAGCACTCACCGTAG - 3’	GAGCTCTCAC**CTGGGAGAGAGGATCAGTCA**AGGTCTCT	None
Intergenic: SRSF1-RP11-159D12.10<br>**chr17:56132133-56132155:+**	FW 5’ - AGACCCTGTTCTTCAGCCAC - 3’<br>RV 5’- GGAATCTGGTCAGTGCTCCC - 3’	TGTGGAAGTC**CTGGGGCACTGGATCAGTCA**AGGTCCTG	None
Intergenic:<br>RP1-124C6.1-AL513123.1<br>**chr6:113798294-113798316:-**	FW 5’ - CTTCTGCCCACAAGGACCAT - 3’<br>RV 5’- AGTTGTAATGGCAGCCCACA 3’	ATGGACTCAT**CTAGGCCAGTGAGTCAGTCA**AGGCATTT	None
Intron: LHX6<br>**chr9:124983136-124983158:-**	FW 5’- TGGGGAGAGGTGTTACTGCT -3’<br>RV 5’- CCTTGTTCGCCTCACAGGAT -3’	GCATAGTAGG**CTGGGAAAGTGAGTCTGACT**TGGTCAAG	None
Intergenic: C10orf91-RP11-432J24.5<br>**chr10:134274249-134274271:+**	FW 5’ - CTGCTCCTTGAGGGACAGTG - 3’<br>RV 5’- CACTGGCCTGGGGAAAGTAG - 3’	GTGGGATCCC**CTGGGGAAGAGGGTCTGTCT**GGGCTCGG	None
Intergenic: AL512655.1-MIR5007<br>**chr13:55536216-55536238:+**	FW 5’- GAGCAGGGCTTCCATCTTGA - 3’<br>RV 5’- TGGAAGCACTTAATGTAAACAGTG - 3’	TGTACTGCTG**TTGAGTCAGTGTGTCTGTCA**GGGAAGAA	None

Table S4: ISO-03-P116 top 10 off-target candidates. Related to figures 3-7.

**Gene Locus**	FW primer sequence (5' to 3')<br>RV primer sequence (5' to 3')	Target Sequence (5' to 3')	Off target mutation
Intron:RP11-132N15.3<br>**chr3:187689636-187689658:+**	FW 5'- TTGGGGCATACAAAGGAGGG - 3'<br>RV 5'- TTTCTGAATAGGCTGCCCGT - 3'	AGCCCGTGTT**CTGGAACAGAGAATCTGTCA**GGGGTGTG	None
Intron:RP1-91J24.3<br>**chr6:144636458-144636480:+**	FW 5' - ACATAAGCCAGCCTGCAGAA - 3'<br>RV 5' - GGCCCTGTCCAACTAGTGTC - 3'	GGATGGCTGG**CAGGGGCAGTAGGTCAGTCA**GGGCACTG	None
Intergenic: NARS-RP11-35G9.3<br>**chr18:55296831-55296853:-**	FW 5'- TCCTAAGGACGCCCTACTCT - 3'<br>RV 5' – TCCCAAACTCAGAGGCGTAAC - 3'	CTCAGCTTCC**CTGGACCAGTGGGTCAGTCA**GGGCGTGT	None
Intergenic: HNRNPA 1P57-AC010739.1<br>**chr2:41,415,579-41,415,601:-**	FW 5'- TGGAGCCAAACTCTCTGGGTT - 3'<br>RV 5'- AATTAGGTTCCTTCCTGGCCT - 3'	TGCCATATTC**CTAGGACACTGGATCAGTCA**AGGCCTAG	None
Intron: LINC01088<br>**chr4:79946400-79946422:+**	FW 5'- CACAGCAGCAACAGTCATGG - 3'<br>RV 5'- TTGTGGAGCACTCACCGTAG - 3'	GAGCTCTCAC**CTGGGAGAGAGGATCAGTCA**AGGTCTCT	None
Intergenic: SRSF1-RP11-159D12.10<br>**chr17:56132133-56132155:+**	FW 5' - AGACCCTGTTCTTCAGCCAC - 3'<br>RV 5'- GGAATCTGGTCAGTGCTCCC - 3'	TGTGGAAGTC**CTGGGGCACTGGATCAGTCA**AGGTCCTG	None
Intergenic:<br>RP1-124C6.1-AL513123.1<br>**chr6:113798294-113798316:-**	FW 5' - CTTCTGCCCACAAGGACCAT - 3'<br>RV 5'- AGTTGTAATGGCAGCCCACA 3'	ATGGACTCAT**CTAGGCCAGTGAGTCAGTCA**AGGCATTT	None
Intron: LHX6<br>**chr9:124983136-124983158:-**	FW 5'- TGGGGAGAGGTGTTACTGCT -3'<br>RV 5'- CCTTGTTCGCCTCACAGGAT -3'	GCATAGTAGG**CTGGGAAAGTGAGTCTGACT**TGGTCAAG	None
Intergenic: C10orf91-RP11-432J24.5<br>**chr10:134274249-134274271:+**	FW 5' - CTGCTCCTTGAGGGACAGTG - 3'<br>RV 5'- CACTGGCCTGGGGAAAGTAG - 3'	GTGGGATCCC**CTGGGGAAGAGGGTCTGTCT**GGGCTCGG	None
Intergenic: AL512655.1-MIR5007<br>**chr13:55536216-55536238:+**	FW 5'- GAGCAGGGCTTCCATCTTGA - 3'<br>RV 5'- TGGAAGCACTTAATGTAAACAGTG - 3'	TGTACTGCTG**TTGAGTCAGTGTGTCTGTCA**GGGAAGAA	None

**Table S5: ISO-02 P128 top 10 off-target candidates. Related to figures 3-7.**			
**Gene Locus**	FW primer sequence (5’ to 3’)<br>RV primer sequence (5’ to 3’)	Target Sequence (5’ to 3’)	Off target mutation
Intron:RP11-132N15.3<br>**chr3:187689636-187689658:+**	FW 5’- TTGGGGCATACAAAGGAGGG - 3’<br>RV 5’- TTTCTGAATAGGCTGCCCGT - 3’	AGCCCGTGTT**CTGGAACAGAGAATCTGTCA**GGGGTGTG	None
Intron:RP1-91J24.3<br>**chr6:144636458-144636480:+**	FW 5’ - ACATAAGCCAGCCTGCAGAA - 3’<br>RV 5’ - GGCCCTGTCCAACTAGTGTC - 3’	GGATGGCTGG**CAGGGGCAGTAGGTCAGTCA**GGGCACTG	None
Intergenic: NARS-RP11-35G9.3<br>**chr18:55296831-55296853:-**	FW 5’- TCCTAAGGACGCCCTACTCT - 3’<br>RV 5’ – TCCCAAACTCAGAGGCGTAAC - 3’	CTCAGCTTCC**CTGGACCAGTGGGTCAGTCA**GGGCGTGT	None
Intergenic: HNRNPA 1P57-AC010739.1<br>**chr2:41,415,579-41,415,601:-**	FW 5’- AGTCTGGAGCCAAACTCTCTG - 3’<br>RV 5’- TTCCTTCCTGGCCTTTGCTTA - 3’	TGCCATATTC**CTAGGACACTGGATCAGTCA**AGGCCTAG	None
Intron: LINC01088<br>**chr4:79946400-79946422:+**	FW 5’- CACAGCAGCAACAGTCATGG - 3’<br>RV 5’- TTGTGGAGCACTCACCGTAG - 3’	GAGCTCTCAC**CTGGGAGAGAGGATCAGTCA**AGGTCTCT	None
Intergenic: SRSF1-RP11-159D12.10<br>**chr17:56132133-56132155:+**	FW 5’ - AGACCCTGTTCTTCAGCCAC - 3’<br>RV 5’- GGAATCTGGTCAGTGCTCCC - 3’	TGTGGAAGTC**CTGGGGCACTGGATCAGTCA**AGGTCCTG	None
Intergenic:<br>RP1-124C6.1-AL513123.1<br>**chr6:113798294-113798316:-**	FW 5’ - CTTCTGCCCACAAGGACCAT - 3’<br>RV 5’- AGTTGTAATGGCAGCCCACA 3’	ATGGACTCAT**CTAGGCCAGTGAGTCAGTCA**AGGCATTT	None
Intron: LHX6<br>**chr9:124983136-124983158:-**	FW 5’- TGGGGAGAGGTGTTACTGCT -3’<br>RV 5’- CCTTGTTCGCCTCACAGGAT -3’	GCATAGTAGG**CTGGGAAAGTGAGTCTGACT**TGGTCAAG	None
Intergenic: C10orf91-RP11-432J24.5<br>**chr10:134274249-134274271:+**	FW 5’ - CTGCTCCTTGAGGGACAGTG - 3’<br>RV 5’- CACTGGCCTGGGGAAAGTAG - 3’	GTGGGATCCC**CTGGGGAAGAGGGTCTGTCT**GGGCTCGG	None
Intergenic: AL512655.1-MIR5007<br>**chr13:55536216-55536238:+**	FW 5’- GAGCAGGGCTTCCATCTTGA - 3’<br>RV 5’- TGGAAGCACTTAATGTAAACAGTG - 3’	TGTACTGCTG**TTGAGTCAGTGTGTCTGTCA**GGGAAGAA	None

Table S6: Karyotyping of hiPS cell lines. Related to figures 2-7.				
CTRL 1 LUMC04iCTRL10	male	P20	46,XY[20]	18 normal metaphases; 2 non-clonal aberration metaphases
CTRL 2 LUMC044iCTRL44	female	P18	46,XX,t(1;9)(p34;p13)[15]/46,XX[5]	5 normal metaphases; 15 metaphases with translocation of which 3 out of 15 in addition non-clonal aberration metaphases 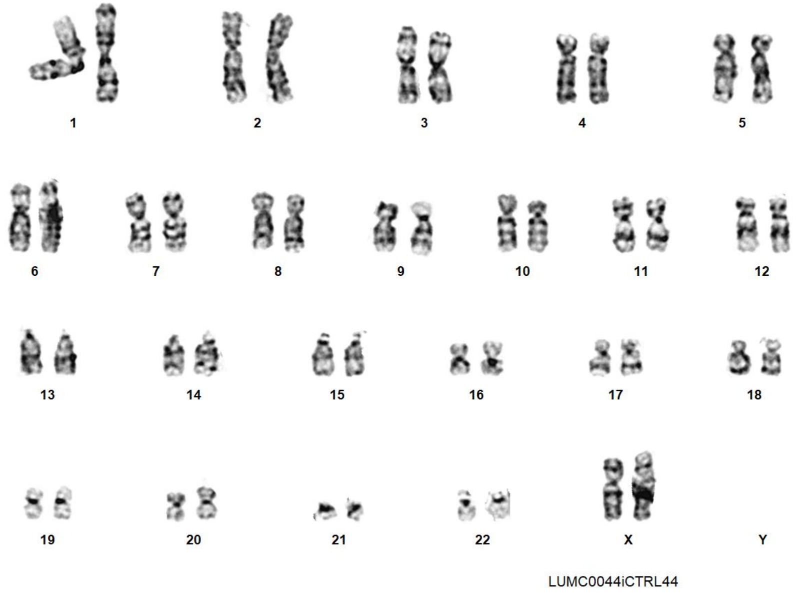
CTRL 3 LUMC080iCTRL12	male	P25	46,XY[20]	20 normal metaphases

				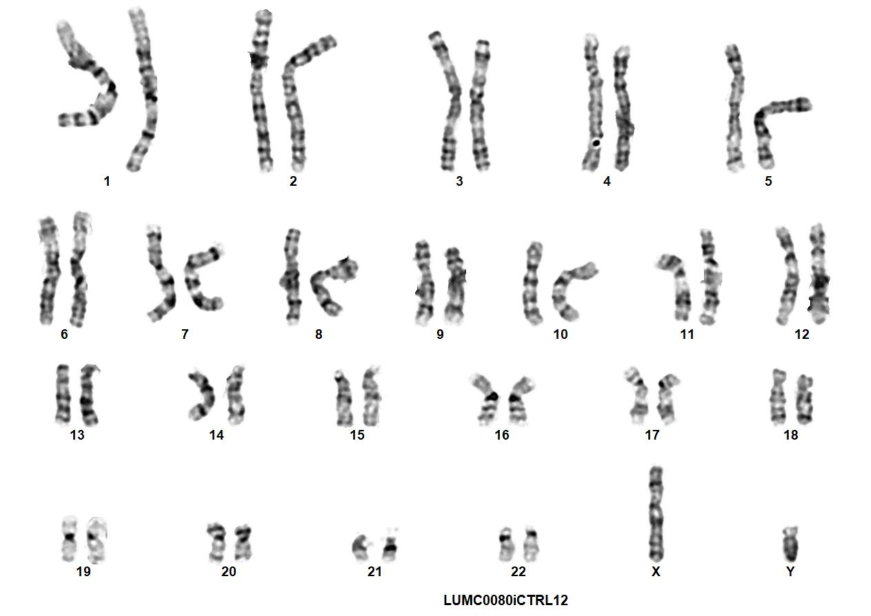
iso02LUMC0116iCRB09	male	P28	46,XY[20]	20 normal metaphases
iso03LUMC0116iCRB09	male	P27	46,XY[20]	20 normal metaphases
iso01LUMC0128iCRB01	male	P25	46,XY[20]	18 normal metaphases; 2 non-clonal aberration metaphases
iso02LUMC0128iCRB01	male	P25	46,XY[20]	19 normal metaphases; 1 non-clonal aberration metaphases
LUMC0116iCRB09	male	P13	46,XY[20]	20 normal metaphases
LUMC0117iCRB01	male	P9	46,XY[20]	19 normal metaphases; 1 non-clonal aberration metaphases
LUMC0128iCRB01	male	P10	46,XY[20]	19 normal metaphases; 1 non-clonal aberration metaphases

Supplemental EXPERIMENTAL PROCEDURES

Animals

Procedures concerning animals were performed with permission of the ethical committee of the Leiden University Medical Center and the animal experimentation committee (DEC) of the Royal Netherlands Academy of Arts and Sciences (KNAW) under permit number NIN 12.105. All mice used were maintained on a 99.9% C57BL/6JOlaHsd genetic background with a 12 h day-night cycle and supplied with food and water *ad libitum*. $Crb1^{KO}$ ($Crb1^{-/-}$) mice (van de Pavert et al., 2004) were crossed with a retinal $Crb2^{\Delta RPC}$ ($Crb2^{F/F}$Chx10$Cre^{Tg/+}$ clone P1E9) mouse strain (Alves et al., 2013b; Rowan and Cepko, 2004) to ablate both *Crb1* and *Crb2* during retinal development from retinal progenitor cells. *Cre* starts to be expressed around E9.5 in the retina (Rowan and Cepko, 2004). The previously made Chx10*Cre* mice were generated by knocking in *GFP-Cre*-IRES-*AP* cDNA in exon-1 of the *Chx10* gene on a Bacterial Artificial Chromosomes (BAC; -55 kb *Chx10* of ATG to +22 kb off of the polyadenylation sequence) (Rowan and Cepko, 2004). Interestingly, the last exon of the *Abcd4* gene is only ~7000 bp from the last exon of the *Chx10* gene thus was on the BAC. Others have shown that large BACS containing several genes can lead to overexpression of flanking genes (Kolisnyk et al., 2013; Ting and Feng, 2014). We believe the integration of the cDNA in close proximity to the *Abcd4* gene and being both on the BAC may have led to the overexpression of the *Abcd4* gene in $Crb1^{KO}Crb2^{\Delta RPC}$ mice. $Crb1^{KO}Crb2^{\Delta RPC}$ mice were compared to littermate $Crb1^{KO}Crb2^{F/F}$ mice not expressing *Cre*. C57BL/6JOlaHsd mice have a 365 kb deletion ablating the *Mmrn1* and *Snca* and express low levels of *Abcd4* (Gajovic et al., 2006; Specht and Schoepfer, 2004). The C57BL/6J substrain (from the Jackson Laboratory) carries a 5-exon-spanning deletion in the *Nnt* gene but C57BL/6JOlaHsd have a wildtype *Nnt* gene (Huang et al., 2006). Analysis of the $Crb1^{KO}Crb2^{\Delta RPC}$ and $Crb1^{KO}Crb2^{F/F}$ mice was carried out on males and females. Chromosomal DNA isolation and genotyping were performed as previously described (Alves et al., 2013a; van de Pavert *et al.*, 2004).

Morphology and retinal thickness measurements on plastic mouse eyes

Eyes were collected of double-knockout and control littermate mice. After enucleation, the eyes were fixed in 4% paraformaldehyde in PBS (25 min) and dehydrated in an ethanol series (30 min each step; 30 % EtOH , 50 % EtOH, 70 % EtOH, 2x 90 % EtOH, 2x 100% EtOH). Then, the eyes were hardened in Technovit, sectioned (3 µm), stained (0.5% Toluidine blue), and mounted (Entellan) as previously described [(Alves and Wijnholds, 2018)]. Consecutive

brightfield images were taken. We generated spidergrams of the retinal thickness (ILM-to-OLM) for P14, 1 M and 3M-old-mice by measuring at 0.25, 0.5, 0.75, 1.0, 1.25, 1.5, 1.75, 2.0, 2.25, 2.5, and 2.75 mm distance to the optic nerve head (ONH) as previously described (Alves and Wijnholds, 2018).

Electroretinography

Electroretinography's (ERGs) were done in dim light on an Espion E2 (Diagnosys, LLC, MA). 1-month-old (1M) mice were dark-adapted (>12 hours). The mice were anesthetized (100 mg/kg ketamine, 10 mg/kg xylazine, intraperitoneal injection) and the pupils were dilated (atropine drops, 5 mg/mL). Scotopic flash series: -4, -3 , -2, -1, 0, 1, 1.5, 1.9 log cd s/m² light intensity. Photopic flash series at 30 cd/m² background light: -2, -1, 0, 1, 1.5, 1.9 log cd s/m² light intensity. We first recorded the scotopic ERG, followed by a 10 min light exposure (30 cd s/m² light intensity) and then the photopic ERG.

RNA sequencing

Sequencing was performed using Life Technologies SOLiD5500 with single-end, 50 bp reads. Two separate runs were performed. Run1 contained n=30 mice; n=5 *Crb1*^{KO}*Crb2*$^{F/F}$ (*Crb1*^{KO}) and n=5 *Crb1*^{KO}*Crb2*$^{\Delta RPC}$ (Tg) per development timepoint of the mouse embryo at E15.5, E17.5 and P1. Run2 contained n=9 mice; n=4 *Crb1*^{KO}*Crb2*$^{\Delta RPC}$ mice (C57BL/6JOlaHsd) and n=5 healthy mice (C57BL/6JRccHsd = wildtype, WT) at E15.5. Reads were aligned against mm10, using the 'whole.transcriptome.frag' workflow with bamgen.mqv.threshold set to 20 (Lifescope v2.5). Counts were obtained using the GTF as supplied by Lifescope (transformation of refGene.txt downloaded from UCSC at 25-06-2014) within this workflow.

Differential expression analysis

The analysis was performed as previously (Pellissier et al., 2013). The two runs were analysed separately. Statistical analyses were performed using the edgeR (Robinson and Oshlack, 2010) and limma/voom (Ritchie et al., 2015) R/Bioconductor packages. One of the *Crb1*^{KO}*Crb2*$^{\Delta RPCC}$ E15.5 samples in the first run was identified as an outlier and removed from the analysis. Genes with more than one count in five or more samples were retained. Count data were transformed to log2-counts per million (logCPM), normalized by applying the trimmed mean of M-values method (Robinson and Oshlack, 2010) and precision weighted using voom (Law et al., 2014). Differential expression was assessed using an empirical Bayes moderated t-test within limma's linear model framework

including the precision weights estimated by voom. Resulting p-values were corrected for multiple testing using the Benjamini-Hochberg false discovery rate. Additional gene annotation was retrieved from Ensembl (release 90) using the biomaRt R/Bioconductor package. Analyses were performed using R v3.4.1 and Bioconductor v3.5.

Real-time quantitative PCR

RNA was isolated from 5 controls (*Crb1*^{KO}) and *Crb1*^{KO}*Crb2*$^{\Delta RPC}$ retinas using TRIZOL reagent (Gibco life technologies), according to the manufacturer manual, and after the final precipitation dissolved in RNase-free water. After genomic DNA degradation with RNase- free DNase I (New England Biolabs), 1 µg of total RNA was reverse transcribed into first-strand cDNA with Superscript III Plus RNase H-Reverse Transcriptase (Invitrogen) and 50 ng random hexamer primers, during 50 min at 50°C in a total volume of 20 µl. To the resulting cDNA sample, 14 µL of 10 mM Tris, 1 mM EDTA was added. From all samples, a 1:20 dilution was made and used for qPCR analysis. For this analysis, primer pairs were designed with a melting temperature of 60–62°C, giving rise to an amplicon of 80–110 bp. Real-time qPCR was based on the real-time monitoring of SYBR Green I dye fluorescence on a ABI Prism 7300 Sequence Detection System (Applied Biosystems, Nieuwekerk a/d IJssel, The Netherlands). The PCR conditions were as follows: 12.5 µL SYBR Green PCR 26mastermix (Applied Biosystems), 20 pmol of primers, and 2 µl of the diluted cDNA (ca 3 ng total RNA input). An initial step of 50°C for 2 min was used for AmpErase incubation followed by 15 min at 95°C to inactivate AmpErase and to activate the AmpliTaq. Cycling conditions were as follows: melting step at 95°C for 1 min, annealing at 58°C for 1 min and elongation at 72°C, for 40 cycles. At the end of the PCR run, a dissociation curve was determined by ramping the temperature of the sample from 60 to 95°C while continuously collecting fluorescence data. Non template controls were included for each primer pair to check for any significant levels of contaminants. Values were normalized by the geometric mean of the 3 reference genes hypoxanthine-guanine phosphoribosyltransferase, elongation factor 1-alpha and ribosomal protein S27a.

Cell culture

We previously described the three male *CRB1* RP-patient human induced pluripotent stem cell (hiPSC) lines (Patient line 1-3: LUMC0116iCRB09, LUMC0117iCRB01, LUMC0128iCRB01) and three control lines (CTRL 1-3: LUMC0004iCTRL10 (hPSC^{reg} name: LUMCi029-B), LUMC0044iCTRL44,

LUMC0080iCTRL12; see also Table S1; (Quinn et al., 2019). Human iPSCs were maintained on mTeSR1 or mTeSR Plus on matrigel-coated plates and stored in mFreSR (STEMCELL Techologies) or CryoStor CS10 in liquid nitrogen for long-term storage. The pluripotency status and functional pluripotency was performed for at least 3 clones per mutated line or isogenic controls (**Figure S3 C**). hiPSC clones were characterized on pluripotency by triple-staining dissociated single cells with OCT3/4, NANOG, and SSEA4 ($1x10^5$ cells/sample; 1 hr at RT) and analyzed by FACS (Flow cytometer, Miltenyi-Vyb). hiPSCs were also differentiated into the three germlayers (STEMdiff Trilineage differentiation kit, Stem Cell Technologies) and stained on coverslips with the conjugated antibodies Nestin (ectoderm), PAX6 (ectoderm), FOXA2 (endoderm), GATA4 (endoderm), vimentin (mesoderm), CDX2 (mesoderm), Brachyury (mesoderm; blocking solution: 4% NSS/PBS with 0.1% Triton X-100; cells incubated with primary antibodies in 4%NSS/PBS for 1 hr at RT). **Figure S3 F-G** and see **Table S2** antibody concentrations).

CRISPR/Cas9-based gene-repair of the hiPSC lines LUMC0116iCRB09 and LUMC0128iCRB01

The *CRB1* variant c.3122T>C (exon 9) was repaired by CRISPR/Cas9 ribonucleoprotein (RNP) mediated homologous recombination. Sequences of the sgRNA and the repair template (ssODN) are provided below. $1x10^5$ single cells were transfected using electroporation (Neon System). The CRISPR/Cas9 RNP complex (crRNA:tracrRNA duplex (IDT); 10 pmol of crRNA/tracrRNA pre-annealed (IDT)) and the Cas9 V3 (1 µg; IDT) was transfected. Additionally, the homology directed (HRD) repair template (ssODN; 40 pmol) was added to correct the mutation. For screening of targeted clones, a restriction site is introduced via a silent mutation to avoid re-cutting after HDR (Figure S3 B). $3.6x10^5$ cells in 27 µL were mixed with 6 µL ssODN (13.3 µM) and 3 µL Cas9/RNP complex (3.6 µg Cas9, 36 pmol pre-annealed crRNA:tracrRNA). Then, three times 10 µL ($1.0x10^5$ cells per tube. 30 µL and $3.0x10^5$ cells in total per line) were electroporated (Neon System; Thermo Fisher Scientific; 1100V, 20 ms, 2 pulses), then plated on mTeSR-E8 + CloneR supplement (STEMCELL Technologies) into 12-well plates coated with Synthemax® II-SC substrate (Corning) and placed into an incubator (37°C, 5% CO_2, 20% O_2). Human iPSCs were seeded as single cells 5-7 days post-electroporation (1000 cells / 10-cm plate). Colonies were picked and gDNA isolated (QuickExtract) 8 days after seeding. Genomic DNA covering the variant region was amplified and targeting was analyzed using Taq1 (NEB; R0149) restriction of the PCR fragment. No off-target integrations were observed (Tables S3-5). All but control clone LUMC0044iCTRL44 showed a normal karyotype (Table S6). Successful targeting was confirmed by Sanger sequencing (*CRB1* gene exon 9. **Figure S3 D-E**).

Cas9 nuclease V3 (IDT #1081058); tracrRNA (IDT #1072532); crRNA (IDT, custom-made)

ssODN: ACAAGTTTGCAGTCAGTGAATGATGGCACATGGCACGAAGTGACCCTTTCGATGACAGACCCACTGTCCCAGACCTCCAGGTGGCAAATGGAAGTGGACA

Sequence of crRNA(s) used: CTGGGACAGTGGGTCTGTCG

Sanger sequencing primers:

Fwd: ACCAGAGAACTCACCAATATCAC

Rev: CAATAGCTCTGTCTCCCACATAA

Retinal organoid differentiation

The differentiation of the hiPSCs started at differentiation day (DD) 0 with floating single-cell hiPSCs that were self-aggregated to embryoid bodies in 10 µM (±)blebbistatin in mTeSR1 or mTeSR Plus medium (STEMCELL Technologies) on 1% agarose-coated (non-adhesive) plates overnight. The media was gradually changed from mTeSR to the Neural Induction Medium (NIM-1) over three days (mTeSR1/NIM-1: day 0, 1:0, day 1, 3:1; day 2, 1:1, day 3, 0:1). NIM-1 consists of DMEM/F12 supplemented with 1xN2 supplement, 1x Minimum Essential Media Non-Essential Amino Acids (MEM NEAAs) and 2 µg/mL Heparin (Sigma. See also (Quinn *et al.*, 2019)). The medium was changed every day until DD7. The embryoid bodies were plated at an approximate density of 20 aggregates per cm^2 onto Matrigel-coated wells on DD7 and lifted on DD22/28. NIM-1 was replaced every other day from DD7 to DD16, from DD16 medium was replaced each day with Neural induction medium 2 (NIM-2). NIM-2: 1x B27 or NeuroCult SM1 without Vitamin A, 1x NEAA and 1x antibiotic-antimycotic in DMEM/F12(3:1). Subsequently, the lifted organoids were dislodged and kept in floating culture with NIM-2 on agarose-coated plates. At DD33 forebrain organoids were removed with a truncated P200 pipet tip. Medium was replaced three times a week from DD41 onwards. The medium was changed to Retinal Lamination Medium 1 (RLM-1) at DD42. RLM-1: 10% FBS, 1x B27 without Vitamin A (Gibco) or NeuroCult™ SM1 without Vitamin A (Stem Cell Technologies), 1x NEAA, 1x antibiotic-antimycotic, and 100 µM taurine in DMEM/F12(3:1). The RLM-1 was supplemented with 1 µM retinoic acid from DD49 to DD98. The long-term culture medium from DD98 onwards was Retinal Lamination Medium-2 (RLM-2) with low amounts of growth factors (N2 supplement instead of B27). RLM-2: 10% FBS, 1x N2, 1x NEAA, 1x antibiotic-antimycotic, and 100 µM taurine in DMEM/F12(3:1) 0.5 µM retinoic acid.

Fixation, sectioning and immunohistochemical staining

Retinal organoids were collected at the time points of differentiation day(DD)90, 120, 150, and 180. Prior to fixation, the organoids were washed shortly in PBS and then incubated for 30 minutes in 4% paraformaldehyde in PBS. Subsequently, the organoids were dehydrated in 15% sucrose in PBS (30 min) and 30% sucrose in PBS (30 min). After that, the retinal organoids were orientated and embedded in OCT Tissue-Tek cryo-embedding media. Finally, they were frozen on dry-ice and stored at -20°C. The retinal organoids were sectioned at 7 µm using a Leica CM1900 cryostat (Leica Microsystems) and after 1 hour of airdrying stored at -20°C. The slides were rehydrated in PBS, blocked (1 hour. 0.1% BSA 0.04% Triton-X in PBS), stained with the primary antibody (in 0.1% BSA 0.04% Triton-X in PBS) overnight at 4°C or 3 hours at room temperature, washed three times in PBS (3x10 min), stained with the secondary antibody (1 hour), washed three times in PBS (3x10 min), and mounted with Vectashield® Vibrance® HardSet antifade mounting media containing DAPI (Vector Laboratories). For antibodies and dilutions see Table S2. A Leica TCS SP8 confocal microscope was used for image acquisition. Image analysis was done in Leica Application Suite X (Leica, LAS X), Fiji ImageJ, and Adobe Photoshop CC2018.

Fluorescence quantification in ROIs

All organoids (7-µm sectioned) imaged for fluorescence semi-quantification were stained (see Table S2) with the same antibody mix at the same time, imaged in one confocal microscopy session, and included a negative control (no primary antibody added). At least six different organoids (generally 10-12) per line on generally three glass slides were analyzed per immunofluorescence staining. Several experiments were stained twice and analyzed twice showing overall high robustness of outcome parameters. The semi-quantification of CRB1 protein expression was done in triplicates with different secondary antibodies and incubation times (overnight at $+4^{o}$C or 3 hours at RT) showing a strong reduction in patient organoids in all conditions. The analysis was done blinded. A 8-bit gray-scale raw image file was loaded into ImageJ. We manually defined three ROIs in the retina: ROI1, OLM (±2.5 µm of OLM); ROI2, outer nuclear layer (dense bright DAPI+ nuclei layer or OTX2+ nuclei layer below the ROI 1), ROI3, OPL/INL (nuclei layer below the ROI2 of spanning the retina maximally 50 µm below ROI2). First, the mean gray value (MGV) per ROI was analysed. Then, we measured on each ROI the average particle size (area), the number of particles (counts), and the average intensity per spot (value between 0-255) using the ImageJ function “Analyze Particles”. We normalized the values to: counts per 100 μm^2 and the area of all spot sizes per ROI area (%).

Isolation of protein lysates and immunoblotting

Retinal organoids were collected at DD180. The RPE (dark parts) were dissected off with surgical scissors. Single retinas were washed in cold 1xPBS and then lysed in cold RIPA buffer (#R0278, Sigma) including the protease inhibitor cocktail (cOmplete, #11836153001, Roche). The single retinas were mechanical disrupted by pipetting and incubated on ice for 2 hours. Then, the retinas were vortexed and then sonicated in a pre-cooled +4^{0}C water bath (Diagenode Biorupter Pico; Program: 15 sec ON, 30 sec OFF. 4 cycles; vortexed between 1st and 2nd cycle). The samples were then spun down (16000xg, 10 min, +4°C) and the supernatant was collected in a new pre-cooled tube. The protein concentration was measured by the Bradford assay (BCA Protein Assay Kit; ThermoFisher, #23227). The protein was then diluted to 1x Laemmli buffer with DTT (Bio-Rad, #1610737. Buffer: 0.12 M Tris-HCl, pH 6.8, 4% SDS, 20% glycerol, 10 mM DTT), boiled for 5 min (+95°C), and stored at -80°C. The protein lysates (20 µg per organoid per lane) were separated on a 4-20% SDS gel (Bio-Rad, #4561094) and transferred on PVDF (Bio-Rad Turbo transfer system. Protocol: mixed-molecular weight). The blots were blocked in 5% non-fatty milk in 1xPBS 0.1% Tween-20 (PBST; RT, 1 hour), incubated with the primary antibody (see Table S2 for concentrations; 5% milk in PBST), washed, incubated with the HPR-conjugated-antibody (RT, 1 hour 5% milk in PBST), and washed again. The signal was generated in ECL substrate (Bio-Rad, #1705061) and the chemiluminescence was imaged at three different exposure times (ChemiDoc MP Imaging System, Bio-Rad). The band intensity was measured (ImageJ, Gel analyzer) and averaged from three exposure times. Quantification was relative to GAPDH.

Transmission electron microscopy

Samples were washed in 1xPBS and then fixed in 1.5% glutaraldehyde in 0.1M cacodylate buffer at room temperature for 1 hour, stored in 0.5% PFA in 0.1M PHEM storage buffer until further processing. Samples were rinsed three times in 0.1 M cacodylate buffer and post-fixed in 1%OsO_4/1.5% potassium ferricyanide in 0.1M cacodylate buffer (1 hour on ice). Samples were rinsed again in 0.1M cacodylate buffer three times, and dehydrated in a series of ethanol, followed by a series of propylene oxide with EPON (LX112, Ladd research industries) and finally a step in 100% EPON. Organoids were put in a mould, those were filled up with EPON and polymerized at 70°C over two days. Ultrathin sections (90 nm) were created on a Reichert Ultracut S (Leica Microsystems) and after staining with uranylacetate and lead citrate, examined on an electron microscope (Microscope: FEI Tecnai T12 Twin Fei Company, Eindhoven, The

Netherlands; Camera: OneView, Gatan) operating at 120 kV. Overlapping images were collected and stitched together into separate images as previously described (Faas et al., 2012).

Conjugation of NOTCH1 and CRB1 antibody to plus and minus oligonucleotide probes

We used the Duolink probemaker set (Sigma-Aldrich) to conjugate two same-species antibodies (mouse anti-NOTCH1 and mouse anti-CRB1 extracellular domain antibodies) to the plus or minus oligonucleotide probes as described in the protocol. In short: 20 µL of the primary antibody was mixed with 2 µL of the conjugation buffer. Subsequently, the antibodies were added to the Duolink In Situ Probemaker plus and minus vials for overnight incubation at room temperature. The next day, the stop reagent was added (incubation: 30 minutes, at RT), the storage solution was added and stored at +4°C.

Proximity ligation assay

For the proximity ligation assay the Duolink In Situ Detection Reagents kit green (Sigma-Aldrich) was used. Slides with sliced organoids were washed, and the tissue slices were circled with a hydrophobic pen. Slides were blocked with one drop of blocking solution from the Duolink PLA probe set per tissue slice and incubated for 1 hour at 37 °C in a humidity chamber. Blocking buffer was tapped of and the conjugated antibodies diluted in PLA probe diluent from the PLA probe kit were added for an overnight incubation at 4°C. After that the slides were washed twice in PLA wash buffer A (Sigma-Aldrich) for five minutes. For the ligation step the slides were incubated for 30 minutes at 37°C with ligase enzyme diluted (1:40) in 1x ligation buffer and were washed twice in wash buffer A again. Finally, the polymerase enzyme was diluted (1:80) in 1x amplification buffer and added to the slides and the slides were incubated in a humidity chamber (37 °C, 100 minutes). To one of the slides diluted amplification buffer was added without the polymerase, serving as a negative control. Subsequently, the slides were washed twice for 10 minutes in PLA wash buffer B and after that once for 1 minute in 0.01x wash buffer B. Wash buffer was tapped off and slides were mounted by adding 5 µL of Duolink In Situ Mounting Medium with DAPI with a 20µl pipet with truncated tip. Images were obtained using a Leica TCS SP8 confocal microscope.

REFERENCES

Alves, C.H., Bossers, K., Vos, R.M., Essing, A.H., Swagemakers, S., van der Spek, P.J., Verhaagen, J., and Wijnholds, J. (2013a). Microarray and morphological analysis of early postnatal CRB2 mutant retinas on a pure C57BL/6J genetic background. PLoS One *8*, e82532. 10.1371/journal.pone.0082532.

Alves, C.H., Sanz, A.S., Park, B., Pellissier, L.P., Tanimoto, N., Beck, S.C., Huber, G., Murtaza, M., Richard, F., Sridevi Gurubaran, I., et al. (2013b). Loss of CRB2 in the mouse retina mimics human retinitis pigmentosa due to mutations in the CRB1 gene. Hum Mol Genet *22*, 35-50. 10.1093/hmg/dds398.

Alves, C.H., and Wijnholds, J. (2018). AAV Gene Augmentation Therapy for CRB1-Associated Retinitis Pigmentosa. Methods Mol Biol *1715*, 135-151. 10.1007/978-1-4939-7522-8_10.

Faas, F.G., Avramut, M.C., van den Berg, B.M., Mommaas, A.M., Koster, A.J., and Ravelli, R.B. (2012). Virtual nanoscopy: generation of ultra-large high resolution electron microscopy maps. J Cell Biol *198*, 457-469. 10.1083/jcb.201201140.

Gajovic, S., Mitrecic, D., Augustincic, L., Iaconcig, A., and Muro, A.F. (2006). Unexpected rescue of alpha-synuclein and multimerin1 deletion in C57BL/6JOlaHsd mice by beta-adducin knockout. Transgenic Res *15*, 255-259. 10.1007/s11248-006-0003-6.

Huang, T.T., Naeemuddin, M., Elchuri, S., Yamaguchi, M., Kozy, H.M., Carlson, E.J., and Epstein, C.J. (2006). Genetic modifiers of the phenotype of mice deficient in mitochondrial superoxide dismutase. Hum Mol Genet *15*, 1187-1194. 10.1093/hmg/ddl034.

Kolisnyk, B., Guzman, M.S., Raulic, S., Fan, J., Magalhaes, A.C., Feng, G., Gros, R., Prado, V.F., and Prado, M.A. (2013). ChAT-ChR2-EYFP mice have enhanced motor endurance but show deficits in attention and several additional cognitive domains. J Neurosci *33*, 10427-10438. 10.1523/JNEUROSCI.0395-13.2013.

Law, C.W., Chen, Y., Shi, W., and Smyth, G.K. (2014). voom: Precision weights unlock linear model analysis tools for RNA-seq read counts. Genome Biol *15*, R29. 10.1186/gb-2014-15-2-r29.

Pellissier, L.P., Alves, C.H., Quinn, P.M., Vos, R.M., Tanimoto, N., Lundvig, D.M., Dudok, J.J., Hooibrink, B., Richard, F., Beck, S.C., et al. (2013). Targeted ablation of CRB1 and CRB2 in retinal progenitor cells mimics Leber congenital amaurosis. PLoS Genet *9*, e1003976. 10.1371/journal.pgen.1003976.

Quinn, P.M., Buck, T.M., Mulder, A.A., Ohonin, C., Alves, C.H., Vos, R.M., Bialecka, M., van Herwaarden, T., van Dijk, E.H.C., Talib, M., et al. (2019). Human iPSC-Derived Retinas Recapitulate the Fetal CRB1 CRB2 Complex Formation and Demonstrate that Photoreceptors and Muller Glia Are Targets of AAV5. Stem Cell Reports *12*, 906-919. 10.1016/j.stemcr.2019.03.002.

Ritchie, M.E., Phipson, B., Wu, D., Hu, Y., Law, C.W., Shi, W., and Smyth, G.K. (2015). limma powers differential expression analyses for RNA-sequencing and microarray studies. Nucleic Acids Res *43*, e47. 10.1093/nar/gkv007.

Robinson, M.D., and Oshlack, A. (2010). A scaling normalization method for differential expression analysis of RNA-seq data. Genome Biol *11*, R25. 10.1186/gb-2010-11-3-r25.

Rowan, S., and Cepko, C.L. (2004). Genetic analysis of the homeodomain transcription factor Chx10 in the retina using a novel multifunctional BAC transgenic mouse reporter. Dev Biol *271*, 388-402. 10.1016/j.ydbio.2004.03.039.

Specht, C.G., and Schoepfer, R. (2004). Deletion of multimerin-1 in alpha-synuclein-deficient mice. Genomics *83*, 1176-1178. 10.1016/j.ygeno.2003.12.014.

Ting, J.T., and Feng, G. (2014). Recombineering strategies for developing next generation BAC transgenic tools for optogenetics and beyond. Front Behav Neurosci *8*, 111. 10.3389/fnbeh.2014.00111.

van de Pavert, S.A., Kantardzhieva, A., Malysheva, A., Meuleman, J., Versteeg, I., Levelt, C., Klooster, J., Geiger, S., Seeliger, M.W., Rashbass, P., et al. (2004). Crumbs homologue 1 is required for maintenance of photoreceptor cell polarization and adhesion during light exposure. J Cell Sci *117*, 4169-4177. 10.1242/jcs.01301.
